# Incidence, Risk Factors, and Epidemiological Trends of Head and Neck Cancer: A Global Analysis

**DOI:** 10.1002/mco2.70467

**Published:** 2025-11-14

**Authors:** Junjie Huang, Shui Hang Chow MSocSc, Mingtao Chen MSocSc, Sze Chai Chan, Jinqiu Yuan, Lin Zhang, Claire Chenwen Zhong, Wanghong Xu, Zhi‐Jie Zheng, Zigui Chen, Jason Y. K. Chan, Martin C. S. Wong

**Affiliations:** ^1^ The Jockey Club School of Public Health and Primary Care, Faculty of Medicine Chinese University of Hong Kong Hong Kong China; ^2^ Centre for Health Education and Health Promotion, Faculty of Medicine The Chinese University of Hong Kong Hong Kong China; ^3^ Clinical Research Center, Big Data Center, The Seventh Affiliated Hospital Sun Yat‐Sen University Shenzhen Guangdong China; ^4^ The School of Public Health and Preventive Medicine Monash University Victoria Australia; ^5^ School of Public Health Fudan University Shanghai China; ^6^ Department of Global Health, School of Public Health Peking University Beijing China; ^7^ Department of Microbiology, Faculty of Medicine The Chinese University of Hong Kong Hong Kong China; ^8^ Department of Otorhinolaryngology, Head and Neck Surgery, Faculty of Medicine The Chinese University of Hong Kong Hong Kong China; ^9^ School of Public Health The Chinese Academy of Medical Sciences and Peking Union Medical College Beijing China

**Keywords:** distribution, head and neck cancer, risk factors, temporal trends

## Abstract

Head and neck cancer (HNC) is a type of common cancer accounting for approximately 4.7% of all incident cancer cases in 2022. This study aims to investigate the trend of HNC on global distribution. We collected related data from the Global Cancer Observatory and Cancer Incidence in Five Continents Plus database, risk factor data at the country level, HDI, and GDP. Multivariable linear regression was conducted to assess HNC and risk factors. Joinpoint regression analysis was used to calculate Average Annual Percentage Change (AAPC). The global age‐standardized rate of HNC incidence was 9.8 in 2022. At the sub‐region level, Melanesia (18.5), South Central Asia (16.2), and Eastern Europe (12.7) had the highest incidence of HNC. At the country level, Bangladesh (23.7) and Papua New Guinea (22.2) had the highest incidence. A higher HNC incidence ratio was associated with higher levels of smoking, alcohol drinking, unhealthy dietary behavior, diabetes, and lipid disorders. There was a rising trend in HNC for the female population, which may be attributed to the increase in female smoking. Mixed trends were observed in the male population, with decreasing trends observed in some high‐HDI countries where smoking is better controlled. Future studies are recommended to investigate the epidemiological changes.

## Graphical abstract

Global incidence of head and neck cancer by regions in 2022.

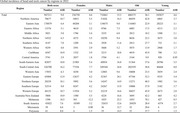

## 1 Introduction

Head and neck cancer (HNC) ranked as the sixth most common type of cancer, with 946,456 incident cases in 2022 as reported by the GLOBOCAN [1]. This cancer comprises a diverse group of malignancies that originate from the upper aerodigestive tract, including cancers of the lip and oral cavity, larynx, nasopharynx, oropharynx, hypopharynx, and salivary glands. Among these cancers, squamous cell carcinoma is the predominant histologic type, representing more than 90% of all HNCs [2]. Its 5‐year (2005–2014) survival rate was 87.4% at the localized stage, but it could also drop to 40.3% if the tumor had been into the distant stage [3]. Some types occur on the overt locations and are typically diagnosed early, such as lip cancers, which usually occur on the lower lip skin, making them easy to diagnose in advance and remove surgically [4, 5]. In contrast, less common types like hypopharyngeal cancer arise from the deep‐seated region of the throat, making them difficult to identify, and their symptoms, such as throat pain or difficulty swallowing, often only manifest at advanced stages [6, 7].

Despite the differences in HNCs, they share numerous risk factors that are clinically significant for their prevention and prognosis. Tobacco and alcohol consumption are acknowledged as the two most important primary risk factors for HNC, especially for cancers of the oral cavity, hypopharynx, and larynx [7, 8, 9, 10, 11, 12]. Also, males are more likely to get HNC compared to females due to their higher prevalence of smoking and drinking behaviors [13]. While not all risk factors contribute to every type of HNC, several have been identified as significant for specific types. For instance, human papillomavirus (HPV) infection is a well‐established risk factor for oropharyngeal cancer, while ultraviolet radiation (UVR) exposure [14] was found to be a risk factor for HNC by some researchers. In addition, Epstein–Barr virus infection is known as a risk factor for nasopharynx and salivary gland cancers [15, 16]. Pottegård et al. conducted a case‐control study and found the association between the diuretic hydrochlorothiazide (HCTZ), a first‐line drug in hypertension treatment, and the risk of HNC [17]. Ramos‐García et al. conducted a meta‐analysis, revealing the association between oral cancer, oral potentially malignant disorders (OPMD), and diabetes mellitus [18]. However, a significant research gap remains, as no systematic analysis has been conducted to evaluate the association between HNC and potential risk factors, accounting for the interplay between different variables. These include smoking, alcohol drinking, physical inactivity, unhealthy diet, obesity, hypertension, lipid disorders, HDI, and GDP.

Meanwhile, many pieces of literature had examined the epidemiological trends of HNC; their discussions were concentrated on specific regions, countries, and types [12, 19, 20, 21, 22, 23, 24, 25]. Moreover, few studies have investigated the relationship between unhealthy lifestyles and HNC. There is a lack of a study comprehensively analyzing the incidence, risk factors, and epidemiology trend of HNC using the latest data. Our study also fills the research gap by comprehensively investigating the global disease burden and trends of HNC by age, sex, and geographical location. The associated risk factors of HNC were examined at the country level.

## 2 Results

### 2.1 Incidence of HNC in 2022

Globally, the ASR of HNC was 9.8 per 100,000 persons, which is a total of 947,211 new cases in 2022 (Table S1). In the variation of sub‐regions, the highest ASR was found in Melanesia (18.5), followed by South Central Asia (16.2), and Eastern Europe (12.7). The lowest ASR was found in Central America (2.9), followed by Western Africa (4.0), and Middle Africa (5.0). Among the countries’ variation, the highest ASR was found in Bangladesh (23.7), followed by Papua New Guinea (22.2), Romania (19.7), and Hungary (19.4). The lowest ASR countries were Sierra Leone (0.04), followed by the Republic of the Gambia (0.99), Sao Tome and Principe (1.6), and Eswatini (1.7).

### 2.2 Incidence of HNC by Subgroup in 2022

In 2022, the male populations reported 710,461 new cases with an ASR of 15.3 worldwide. The new cases of males were larger than females (236,750 new cases, with an ASR of 4.6). For different sub‐regions, Melanesia (25.3 vs. 12.2 in females), South Central Asia (24.8 vs. 7.7), Eastern Europe (24.1 vs. 4.2), and the Caribbean (18.8 vs. 3.9) reported the highest incidence of males. For different countries, Romania (37.0 vs. 4.3 in females) occupied the highest incidence of males, followed by Bangladesh (35.9 vs. 11.0), Moldova (34.5 vs. 1.5), Belarus (34.4 vs. 3.0), Hungary (33.6 vs. 7.5), and Cuba (33.2 vs. 5.8) (Table S1). Regarding the age group, the ASR of the older population (aged 50–74, ASR: 36.1, 606,375 new cases reported) was evidently higher than the younger population (aged 15–49, ASR: 4.6, 185,837 new cases reported) in 2022. Within sub‐regions, the highest ASR of the older population was found in Melanesia (70.1 vs. 8.5 in the younger age group), followed by South Central Asia (58.8 vs. 8.0), Eastern Europe (52.3 vs. 5.4), and Western Europe (45.9 vs. 2.8). Within countries, the highest ASR of the older population was found in Bangladesh (88.3 vs. 10.6 in the younger age group), followed by Papua New Guinea (84.4 vs. 9.7), Hungary (82.6 vs. 7.0), Cuba (79.0 vs. 5.4), Romania (78.0 vs. 9.1), and Slovakia (72.2 vs. 8.6) (Table S2).

### 2.3 Associations of Risk Factors With Incidence of HNC

The overall incidence of HNC showed a significant positive association with prevalence of smoking (*β* = 0.037, 95% CI: 0.020–0.054, *p* < 0.001), alcohol drinking (beta coefficients [*β*] = 0.033, 95% confidence intervals [CI]: 0.014–0.052, *p* < 0.001), unhealthy dietary behavior (*β* = 0.012, 95% CI: 0.003–0.021, *p* = 0.010), diabetes (*β* = 0.020, 95% CI: 0.002–0.039, *p* = 0.032), and lipid disorders (*β* = 0.016, 95% CI: 0.008–0.024, *p* < 0.001) (Table S3).

### 2.4 Associations of Risk Factors With Incidence of HNC by Subgroup

Regarding the male gender group, higher HNC incidence was associated with higher prevalence of smoking (*β* = 0.043, 95% CI: 0.024–0.062, *p* < 0.001), alcohol drinking (*β* = 0.044, 95% CI: 0.023–0.064, *p* < 0.001), diabetes (*β* = 0.023, 95% CI: 0.003–0.044, *p* = 0.027), and lipid disorders (*β* = 0.018, 95% CI: 0.009–0.027, *p* < 0.001).

**FIGURE 1:**
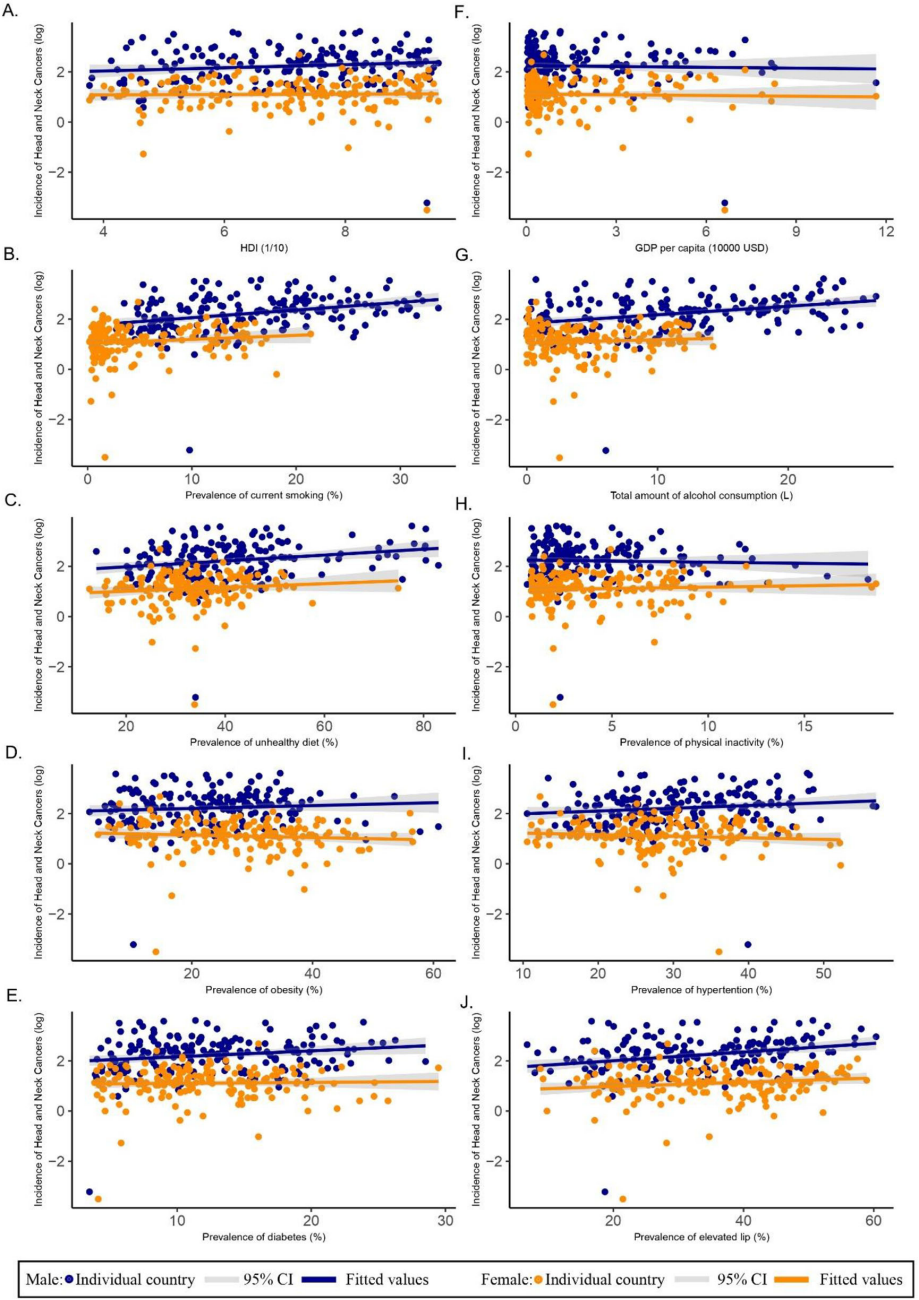
Associations between risk factors and head and neck cancers by sex. (A) Incidence of head and neck cancers versus HDI (Human Development Index). (B) Incidence of head and neck cancers versus prevalence of current smoking (%). (C) Incidence of head and neck cancers versus prevalence of unhealthy diet (%). (D) Incidence of head and neck cancers versus prevalence of obesity (%). (E) Incidence of head and neck cancers versus prevalence of diabetes (%). (F) Incidence of head and neck cancers versus GDP per capita ($10,000). (G) Incidence of head and neck cancers versus total amount of alcohol consumption (L). (H) Incidence of head and neck cancers versus prevalence of physical inactivity (%). (I) Incidence of head and neck cancers versus prevalence of hypertension (%). (J) Incidence of head and neck cancers versus prevalence of elevated lipids (%).

Regarding the female gender group, only the prevalence of smoking (*β* = 0.018, 95% CI: 0.000–0.035, *p* = 0.049) and lipid disorder (*β* = 0.009, 95% CI: 0.001–0.017, *p* = 0.038) were significant risk factors related to higher incidence (Figure 1).

Regarding the younger age group, a higher prevalence of smoking (*β* = 0.025, 95% CI: 0.007–0.044, *p* = 0.008), unhealthy dietary behavior (*β* = 0.012, 95% CI: 0.002–0.021, *p* = 0.015), and lipid disorder (*β* = 0.011, 95% CI: 0.002–0.020, *p* = 0.017) were risk factors that associated with higher prevalence of HNC.

**FIGURE 2:**
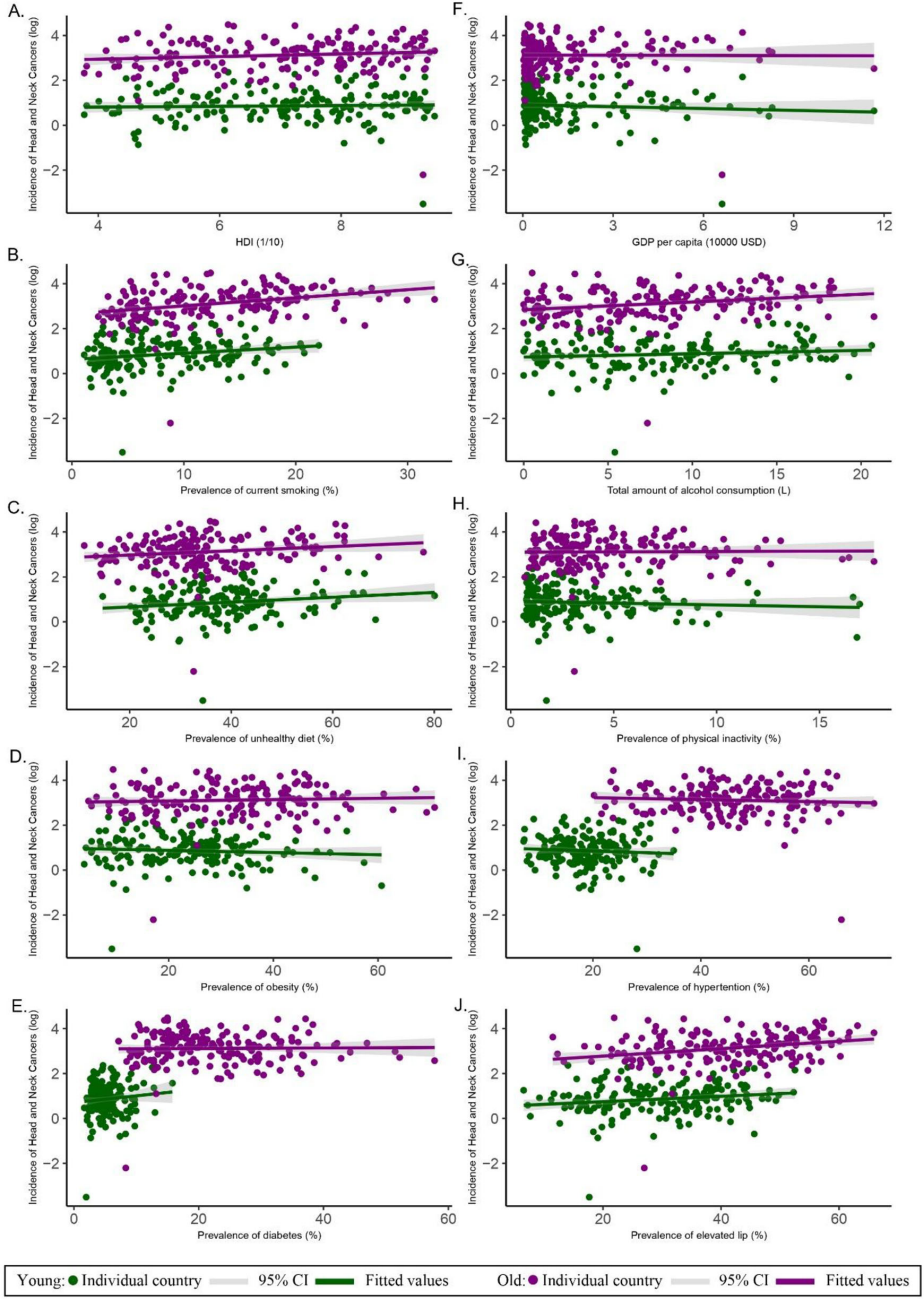
Associations between risk factors and head and neck cancers by age. (A) Incidence of head and neck cancers versus HDI (Human Development Index). (B) Incidence of head and neck cancers versus prevalence of current smoking (%). (C) Incidence of head and neck cancers versus prevalence of unhealthy diet (%). (D) Incidence of head and neck cancers versus prevalence of obesity (%). (E) Incidence of head and neck cancers versus prevalence of diabetes (%). (F) Incidence of head and neck cancers versus GDP per capita (10,000 USD). (G) Incidence of head and neck cancers versus total amount of alcohol consumption (L). (H) Incidence of head and neck cancers versus prevalence of physical inactivity (%). (I) Incidence of head and neck cancers versus prevalence of hypertension (%). (J) Incidence of head and neck cancers versus prevalence of elevated lipids (%).

Meanwhile, higher prevalence of smoking (*β* = 0.043, 95% CI: 0.024–0.061, *p *< 0.001), alcohol drinking (*β* = 0.040, 95% CI: 0.020–0.060, *p* < 0.001), unhealthy dietary behavior (*β* = 0.012, 95% CI: 0.003–0.022, *p* = 0.013), diabetes (*β* = 0.021, 95% CI: 0.001–0.041, *p* = 0.043) and lipid disorders (*β* = 0.017, 95% CI: 0.008–0.026, *p* < 0.001) were the risk factors associated with HNC incidence in the older age group (Figure 2; Table S3).

### 2.5 Sensitivity Analysis on Risk Factor Associations

For multicollinearity assessment, all VIFs ranged between 1 and 5, suggesting a moderate correlation between variables (Table S4). The finding indicated a reliable result, as shown by a relatively acceptable VIF. After adjustment, smoking reminded as a risk factor for HNC (*β* = 0.029, 95% CI: 0.001–0.056, *p* = 0.041). In addition, GDP per capita (*β* = −0.083, 95% CI: −0.157 to −0.010, *p* = 0.026) were found significantly with HNC when excluded countries that used estimates. Whereas the dietary habits, diabetes, and lipid disorders were not significantly associated with HNC.

For sensitivity analysis, the results used GLOBOCAN 2022 outcomes paired with 2012 GBD risk factor data (Table S5). Overall, HDI (OR: 0.149, 95% CI: 0.087–0.211, *p* < 0.001), and GDP per capita (OR: 0.072, 95% CI: 0.020–0.123, *p* = 0.007) were found to be associated with HNC in addition to the original findings of risk factors, such as smoking, alcohol drinking, and lipid disorder. In a separate sensitivity analysis that, the results excluded 38 countries that utilized the estimated number (Table S6). Overall, HDI (*β* = 0.099, 95% CI: 0.038–0.159, *p* = 0.002) and Hypertension (*β* = 0.015, 95% CI: 0.005–0.026, *p* = 0.005) were found to be associated with HNC in addition to the original findings of risk factors, such as smoking, alcohol drinking and dietary habits.

### 2.6 Trend Analysis for Incidence of HNC

In general, the incidence trend of HNC demonstrated mixed trends with 14 countries showing significant increasing trends and 13 countries showing significant decreasing trends (Tables S4 and S7; Figure S1). The country with the largest increasing trend of the HNC incidence was Spain (AAPC: 10.07, 95% CI 3.16–14.29, *p* = 0.002), followed by Italy (AAPC: 8.36, 95% CI 2.49–12.00, *p* = 0.004), India (AAPC: 8.31, 95% CI 4.15–11.07, *p* < 0.001), the United Kingdom (AAPC: 8.25, 95% CI 4.66–11.45, *p* < 0.001), and China (AAPC: 7.12, 95% CI 3.63–9.72, *p* < 0.001). Meanwhile, the country with the greatest downward trend was Turkey (AAPC: −5.94, 95% CI −6.42 to −5.45, *p* < 0.001), followed by Israel (AAPC: −4.10, 95% CI −5.13 to −3.68, *p* < 0.001), Malta (AAPC: −3.28, 95% CI −4.04 to −2.19, *p* < 0.001), Switzerland (AAPC: −3.17, 95% CI −3.97 to −2.38, *p* < 0.001), Denmark (AAPC: −2.73, 95% CI −4.46 to −1.00, *p* = 0.001), and France (AAPC: −2.66, 95% CI −4.68 to −0.61, *p* = 0.014). There is no statistically significant trend observed for the remaining 18 countries.

### 2.7 Age‐ and Sex‐Specific Trend Analysis by Subgroup

**FIGURE 3:**
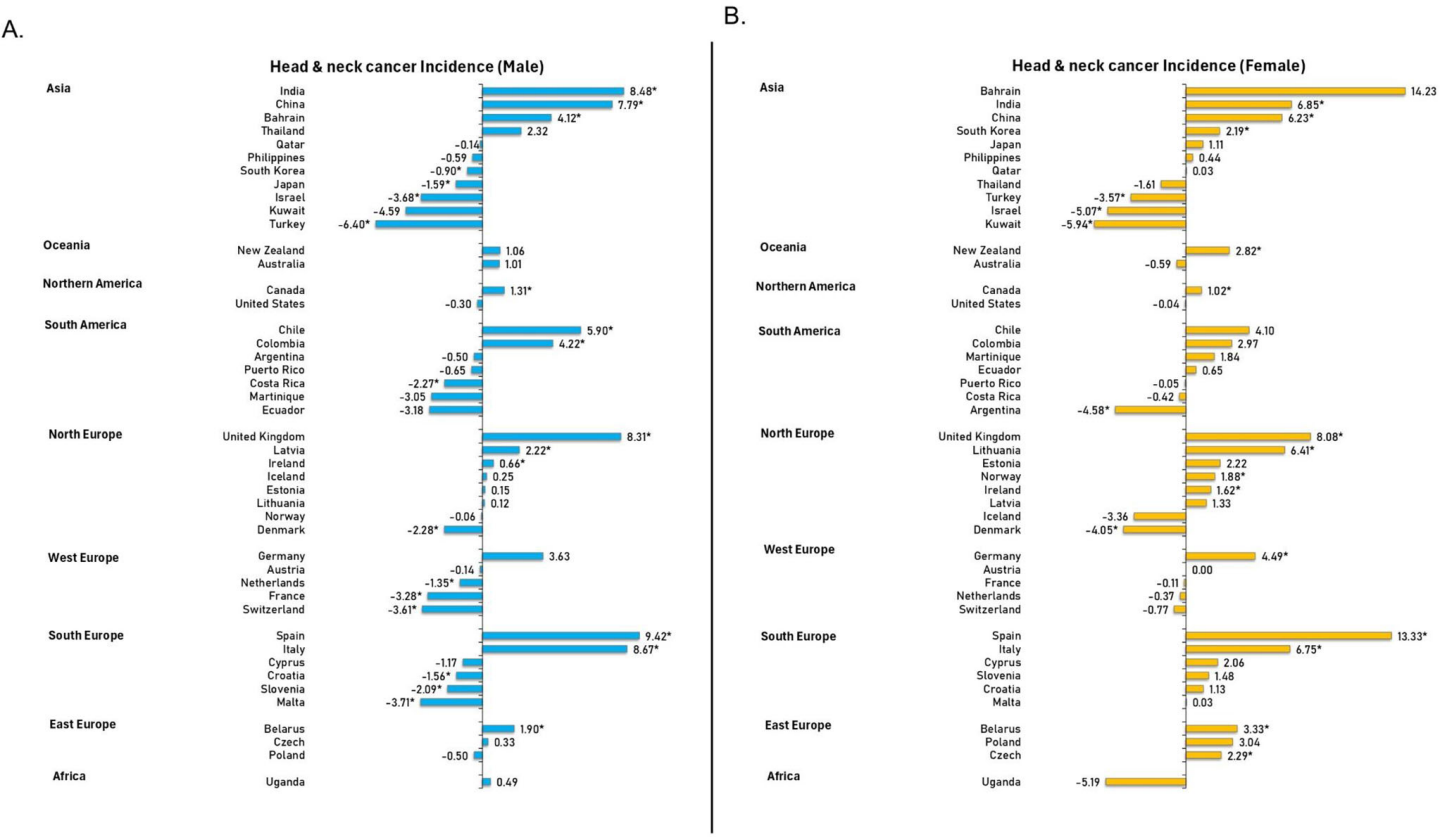
AAPC of head and neck cancers incidence by sex, all ages. (A) Average annual percent change (AAPC) of head and neck cancers incidence in males (all ages). (B) Average annual percent change (AAPC) of head and neck cancers incidence in females (all ages).

There was a mixed trend in HNC incidence among the male population, with the incidence rising in 12 countries and decreasing in 12 other countries (Figure 3). The top five highest rising trend was found in the Spain (AAPC: 9.42, 95% CI 2.66–13.57, *p* = 0.005), Italy (AAPC: 8.67, 95% CI 3.86–11.78, *p* < 0.001), India (AAPC: 8.48, 95% CI 4.53–11.32, *p* < 0.001), the United Kingdom (AAPC: 8.31, 95% CI 4.12–11.77, *p* < 0.001), and China (AAPC: 7.79, 95% CI 4.74–10.5, *p* < 0.001). The top five countries with a significant decreasing incidence trend include Turkey (AAPC: −6.40, 95% CI −6.92 to −5.81, *p* < 0.001), Malta (AAPC: −3.71, 95% CI −5.37 to −2.00, *p* < 0.001), Israel (AAPC: −3.68, 95% CI −4.90 to −3.19, *p* < 0.001), Switzerland (AAPC: −3.61, 95% CI −4.59 to −3.02, *p* < 0.001), and France (AAPC: −3.28, 95% CI −5.48 to −1.09, *p* = 0.006). The other 21 countries did not show a significant increasing or decreasing trend in the specific period. For females, a rising trend was presented in which 14 countries were showing increasing trends, and 5 countries were showing decreasing trends (Figure 3). The top five highest rising trend was observed in Spain (AAPC: 13.33, 95% CI 7.35–17.11, *p* < 0.001), the United Kingdom (AAPC: 8.08, 95% CI 4.68–10.76, *p* < 0.001), India (AAPC: 6.85, 95% CI 2.19–10.76, *p* = 0.002), Italy (AAPC: 6.75, 95% CI 0.27–11.06, *p* = 0.038, and Lithuania (AAPC: 6.41, 95% CI 4.99–7.91, *p* < 0.001). The significant downtrend was observed in Kuwait (AAPC: −5.94, 95% CI −8.86 to −2.98, *p* < 0.001), Israel (AAPC: −5.07, 95% CI −6.40 to −3.80, *p* < 0.001), Argentina (AAPC: −4.58, 95% CI −7.92 to −1.37, *p* = 0.006), Denmark (AAPC: −4.05, 95% CI −5.68 to −2.43, *p* < 0.001), and Turkey (AAPC: −3.57, 95% CI −4.34 to −2.90, *p* < 0.001). Besides, there was no significant increase or decrease incidence trend observed among the other 26 countries during this period.

**FIGURE 4:**
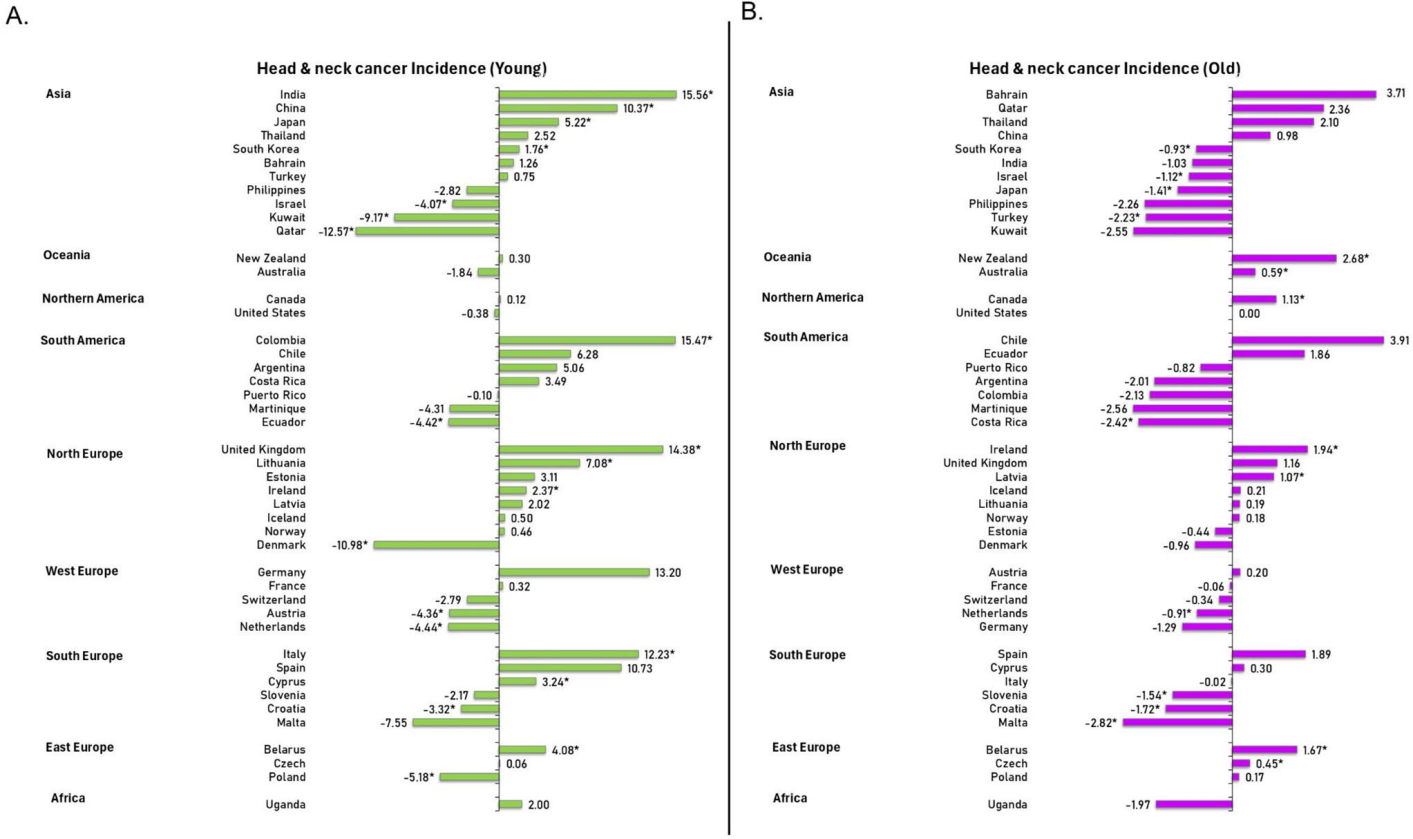
AAPC of head and neck cancers incidence by age, both sexes. (A) Average annual percent change (AAPC) of head and neck cancers incidence in younger age groups (both sexes). (B) Average annual percent change (AAPC) of head and neck cancers incidence in older age groups (both sexes).

Mixed trends were found in both the old population and the young population. For the older age group, seven countries exhibited significant increasing trends in the incidence of HNC, including New Zealand (AAPC: 2.68, 95% CI 1.51–3.64, *p* < 0.001), Ireland (AAPC: 1.94, 95% CI 1.52–2.21, *p* < 0.001), Belarus (AAPC: 1.67, 95% CI 1.06–2.26, *p* < 0.001), Canada (AAPC: 1.13, 95% CI 0.30–1.92, *p* = 0.007), Latvia (AAPC: 1.07, 95% CI 0.14–1.91, *p* = 0.025), Australia (AAPC: 0.59, 95% CI 0.07–1.07, *p* = 0.026), and Czech (AAPC: 0.45, 95% CI 0.07–0.81, *p* = 0.024). Whereas nine countries were found with the downward trends, including Malta (AAPC: −2.82, 95% CI −4.67 to −1.06, *p* = 0.002), Costa Rica (AAPC: −2.42, 95% CI −4.50 to −0.62, *p* = 0.005), Turkey (AAPC: −2.23, 95% CI −3.40 to −1.08, *p* < 0.001), Croatia (AAPC: −1.72, 95% CI −2.10 to −1.26, *p* < 0.001), Slovenia (AAPC: −1.54, 95% CI −2.57 to −0.56, *p* = 0.002), Japan (AAPC: −1.41, 95% CI −2.98 to −0.09, *p* = 0.035), Israel (AAPC: −1.12, 95% CI −2.00 to −0.32, *p* = 0.008), South Korea (AAPC: −0.93, 95% CI −1.29 to −0.59, *p* < 0.001), and Netherland (AAPC: −0.91, 95% CI −1.08 to −0.76, *p* < 0.001). The remaining 29 countries did not show either a significant rising or falling incidence trend of HNC throughout the period (Table S4, Figure 4). On the other hand, in the younger age group, a total of 11 countries showed significantly increasing incidence trend, whereas 9 countries showed significantly declining incidence trend. The top five countries with rising incidence trends include India (AAPC: 15.56, 95% CI 8.06–22.53, *p* < 0.001), Colombia (AAPC: 15.47, 95% CI 3.23–28.67, *p* = 0.013), United Kingdom (AAPC: 14.38, 95% CI 4.50–24.62, *p* = 0.003), Italy (AAPC: 12.23, 95% CI 3.95–20.62, *p* = 0.005), and China (AAPC: 10.37, 95% CI 3.52–17.13, *p* = 0.002). The top five countries with decreasing incidence trends include Qatar (AAPC: −12.57, 95% CI −23.09 to −2.34, *p* = 0.021), Denmark (AAPC: −10.98, 95% CI −19.01 to −2.29, *p* = 0.018), Kuwait (AAPC: −9.17, 95% CI −16.28 to −2.08, *p* = 0.015), Poland (AAPC: −5.18, 95% CI −7.69 to −2.50, *p* = 0.003), and Netherland (AAPC: −4.44, 95% CI −6.01 to −2.52, *p* < 0.001); the other 25 countries did not show significant change in this period of time (Table S4).

## 3 Discussion

In this study, the updated cancer registries were used to comprehensively assess disease burden, risk factors, and trends in different subgroups of HNC. The main findings are (1) there are regional differences of the incidence of HNC. Some countries in Eastern Europe, Oceania, and South‐Central Asia have the highest ASR. (2) Smoking, alcohol drinking, unhealthy dietary behavior, diabetes, and lipid disorders are associated with HNC. (3) The incidence trend shows a mixed trend, with the exception in the female population, which has experienced a rising trend.

The study found significant differences in the regional distribution of HNC. Globally, ASR for HNC is the highest in Oceania and Europe, which is two to six times higher than that in Africa and America. A systematic review reveals the prevalence of tobacco use and exposure remains in a the high level (∼20% smokers among participating males) for the last 20 years despite the implementation of MPOWER measures by WHO for tobacco control [26]. The smoking prevalence was highest in Oceania and Europe [27], which might explain the high ASR for HNC in these regions. At the national level, we found some countries in Oceania, such as Papua New Guinea and Australia, have the highest ASR of this cancer, and in line with previous studies, which have also reported a similar trend [11, 28]. That may relate to the diet habit, such as consumption and use of betel nuts in these areas [29]. A population‐based cohort study of 76,473 patients reported that Native Hawaiian and Other Pacific Islander individuals were likely to get more advanced‐stage HNC and have poorer survival outcomes compared to Asian and non‐Hispanic White individuals [30]. The underlying ethical difference might be the reason. In regard to the higher prevalence of HNC in Europe, a study implied that the higher HNC incidence among European countries was attributed to the increase in oropharyngeal cancer caused by HPV infection [31]. Sierra Leone reported the lowest ASR among countries involved in this study. Studies in the United States and South Africa have shown that the light‐skinned population has a higher incidence and risk of lip cancer compared to the dark‐skinned population, and melanin is more protective against ultraviolet light [32, 33]. Therefore, racial genetic differences and the protective effects of natural pigments may account for the differences. This might explain why regions of Africa have the lowest ASR, despite the poor medical conditions and similarly high smoking prevalence [34].

Smoking and alcohol drinking were identified as the main risk factors for HNC in the overall population in this study, and that result validates the findings of previous research [35, 36, 37, 38]. A study in the United States found that smoking leads to a 4.6 times (95% CI: 1.6–13.4) higher risk for incidence of oral‐related cancer than non‐smokers [39]. Another study from the United States reported that smoking increased the risk of HNC subsites that were close to the lungs, and heavy drinking also increased the risk of HNC [38]. Moreover, several studies conducted in Canada [40], Finland [41], and the Netherlands also found an association of lip cancer and tobacco smoking, which suggested the phenomenon was across different geographical areas and races in a long period of surveillance time [41, 42]. Studies also revealed that smoking and alcohol drinking were associated with the occurrence of HNC in the larynx and hypopharynx [43, 44].

In addition, we have identified other risk factors related to lifestyles and unhealthy conditions, such as unhealthy dietary and lipid disorders, as being associated with the incidence of HNC. Known studies have found that the intake of foods rich in animal fats is associated with the incidence of oral cavity‐related cancers [45]. An epidemiology study showed that high intake of fruits, vegetables, and lean protein was associated with lower risk of HNC, while high intake of fried foods, high‐fat and processed meats, and sweets was associated with higher risk of laryngeal cancer [46]. Therefore, a healthy diet plays important role in preventing the development of HNC. However, we found significant sex differences in risk factors related to lifestyle and behavior in this study. We found alcohol drinking to be only associated with the risk of HNC in men, but not in women. This discrepancy might be attributed to the less frequent alcohol drinking behavior among women compared to men [47]. However, previous studies suggested that alcohol drinking behavior also increased the risk of HNC in women and can impact women more than men [37, 48]. This implied that although alcohol drinking can raise the risk of HNC in women, it was not a significant contributor to HNC in women at the population level. Other risk factors, including smoking and lipid disorders, were the main contributors to HNC in women.

Furthermore, we observed that diabetes is a risk factor for the incidence of HNC across all subgroups, except for females. Previous studies have pointed out similar results, that people with diabetes have a higher incidence of HNC, especially oral‐related cancer [49, 50]. The exact relationship remains unknown due to the influence of cofactors. However, the use of some hypoglycemic drugs may contribute to this increased incidence; a review related to sulfonylureas concludes that the use of sulfonylureas may induce cancer and is associated with the incidence of all types of cancer [51]. Besides, diabetes affects the incidence of HNC in the young age group, which could be explained by the usage of dental services and HCTZ. A report in the United States [52] points out that sugar intake in the young age group (age: 2–49) is far above the recommended intake, which also reflects the global situation. And that high consumption of sugar is directly related to dental care service activity [53]. HCTZ, an agent used in dental services, has been shown to increase the risk of HNC by 1.2 times (95% CI: 0.7–2.2) to 5.5 times (95% CI: 4.2–7.2) depending on cumulative use of HCTZ [17].

Our study identified several risk factors in related to the incidence of HNC. On top of that, we also identified risk factors for subgroups, such as male, female, young and old populations, and so forth. Some risk factors, including smoking and alcohol drinking were previous identified for oral related cancer [54]. While other factors such as physical inactivity may need further studies for identification of potential pathology. The risk factors found in this study provided a correlation with HNC. The causation of HNC across different demographics and geographical areas may need further study to proven.

An overall mixed trend in HNC was observed globally. However, significant downward trend was observed in high HDI and high‐income countries [55, 56], such as the United States, Japan, and Denmark, which may relate to the citizens of high HDI countries more concerned about health‐related quality of life and health education [57, 58]. This may be related to the stricter tobacco control and decreasing smoking prevalence observed in these high HDI and high‐income countries. Meanwhile, significant increasing trend was observed in countries of Oceania and Europe, which are regions with highest smoking prevalence [27]. In addition, a study implied that the rising HNC incidence among European countries was attributed to the increase in oropharyngeal cancer caused by HPV infection [31].

Interestingly, female was the only subgroup with an increasing trend of HNC. Similar findings were reported by a population‐based analysis from Poland, revealing a fast‐rising incidence of oral and oropharyngeal cancers in women [59]. The study attributed the observed rising trend among women to the exposure of HPV [59], a well‐known risk factor for oral and oropharyngeal cancers. In addition, the rising trend may be attributable to the high prevalence of female smoking globally. According to a systematic review, the pooled prevalence of ever and current smoker in female was 28% and 17%, respectively, being high among adolescents, adults, and pregnant women [60]. In the United Kingdom, the country with the highest ASR of HNC among female population, a rising prevalence of smoking among women of reproductive age has also been reported [61]. This further supports the hypothesis that increased smoking rates among women are actively contributing to the rising incidence of HNC in this demographic.

Variations in healthcare systems and cancer reporting practices could have a significant impact on observed cancer trends. First, inconsistent data collection methods may lead to underreporting or misclassification. The cancer data of some low and low‐middle‐income countries may misclassify and underreport due to poor infrastructure, inadequate cancer registry coverage, and insufficient analytical capacity. Similar problems may arise in some information‐closed countries. Moreover, some regions may lack comprehensive cancer registries. Second, a poor healthcare system could affect the early detection and diagnosis of cancer. Therefore, the high incidence rate in developed countries may be due to a more comprehensive screening program. Third, the reporting system could also affect the number of incidences as well. To address the potential bias in the trend analysis, promotion of the adoption of standardized reporting guidelines and diagnostic criteria globally could be addressed. Furthermore, more studies could be conducted comparing regions with different healthcare systems to identify reporting biases in various regions.

Our study focused on the incidence, trends of HNC and its associated risk factors. Our study had not covered the clinical staging, treatment information, or prognostic data of HNC incorporated to the epidemiological research, but the findings of this study could also provide a new scope for development of treatment, drugs and prevention of HNC. First, our study confirmed smoking were associated with the incidence of HNC, highlighting the importance of continued public health efforts in smoking cessation, particularly targeting populations with high smoking rates. Second, our findings provide evidence for recommending regular screenings for high‐risk groups, including older males, to catch HNC early when treatment is more effective. Third, our findings encouraged collaboration among dermatologists, oncologists, and primary care providers to ensure comprehensive care.

Although this study used complete and high‐quality cancer data to derive results on global disease burden, risk factors in different subgroups, and HNC trends, the study still has some limitations in terms of data source and study design. First, the cancer data of some low and low‐middle‐income countries may misclassify and underreport due to poor infrastructure, inadequate cancer registry coverage, and insufficient analytical capacity. Similar problems may arise in some information‐closed countries. Second, the identification of risk factors was only conducted at the country level and may be influenced by the existence of co‐founders. Third, our study generalized the information in countries level which might potentially overlooked variation between regions and community specific factors. Furthermore, the cultural difference and the policy variability across different countries also been overlooked in this study. Fourth, we did not categorize different types of HNC in our study, which hindered our ability to determine which specific type contributed to the observed regional trend in HNC incidence. Fifth, the biological mechanism and the social culture of each population were not interpreted and investigated in this study. The difference may potentially affect the exposures to risk factors which caused bias between countries. Sixth, despite the GBD missioned in providing information on population health, there were still data were not shared in some countries. The year‐to‐year data may not be comparable due to changes in data source and data availability.

## 4 Conclusion

The global disease burden of HNC is significantly different across diverse regions, which can be attributed to the varying levels of emphasis placed on the management of risk factors across different countries. The high burden may persist until the change in consumption and use of betel nuts in Oceania. The management of HPV infection is important to control the burden of HNC in Europe. On the other hand, this research pointed out that lifestyle and unhealthy dietary conditions were associated with HNC. It is suggested that the government should provide a guide and education related to dietary habits.

In addition, the downtrend of HNC observed in some countries may be attributed to the decline in smoking behavior, especially for the male population and the older population, which proves the anti‐smoking is helpful to release the burden of HNC. However, that trend was less pronounced among the female population, which may be related to the stable or rising smoking rate and HPV incidence. Furthermore, downward trends were observed in countries with higher HDI, which may be caused by the improvement on health‐related quality of life and health education in some developed countries. Therefore, further study may focus on the reason for that difference and whether other lifestyle factors affect female HNC incidence.

## 5 Methods and Materials

### 5.1 Data Sources

The Global Cancer Observatory (GCO) was a platform focused on presenting the statistics of global cancer. The GCO mainly collected data from IARC's Cancer Surveillance Branch (CSU) which collects, analyses, interprets, and disseminates data of Cancer from local cancer registry worldwide [62]. The GCO provided databases including GLOBOCAN and Cancer Incidence in Five Continents (CI5) which utilized in this study.

The Global Cancer Observatory (GLOBOCAN) database was utilized to investigate the incidence and age‐standardized rate (ASR) of HNC for 185 countries or territories of the world. Missing data were handled by the use of short‐term predictions and the use of modeled mortality‐to‐incidence ratios [63]. The database, established by the International Association of Cancer Registries in partnership with population‐based cancer registries and the World Health Organization, records a total of 26 cancer types worldwide. Incidence‐to‐mortality ratios estimation, and trends prediction could be achieved through utilizing data from international or national cancer registries [64], as well as approximate nearby countries. The Cancer Today database of the GLOBOCAN was utilized to investigate the incidence and ASR of HNC, including cancers of the lip, oral cavity, salivary gland, pharynx, and larynx. The data source of Cancer Today relied on the data from national cancer registries, but also publicly available information where the national registry was not available.

The *CI5 Plus* database was retrieved for the current decade of cancer incidence in 108 countries to decide the temporal trends of incidence of HNC [65]. It includes cancer incidence yearly from more than 100 cancer registries by multiple population groups and contains contemporary and historical data. The superb data from *CI5 Plus* was applied for time trend examination and time alteration interpretation.

The Global Burden of Disease (GBD) database 2021 was retrieved to serve as the source of data for lifestyle risk factors in various countries, encompassing information on the prevalence of smoking, alcohol consumption, unhealthy diet, and other relevant factors [66]. The definition of smoking adopted in this study referred to the current use of a tobacco product on a daily or occasional basis [27, 67]. For alcohol consumption, it has been defined as deaths assigned to alcohol use disorders or accidental poisoning by alcohol codes, cases of alcohol dependence, a substance‐related disorder involving a dysfunctional pattern of alcohol use, and fatal alcohol syndrome [68]. Unhealthy diet referred to intake of any processed meat [69], red meat [70], high sodium intake [71], high sugar‐sweetened beverages intake [72], diet high in trans fatty acids [73], diet low in calcium [74], fiber [75], low fruit intake [76], diet low in legumes [77], milk [78], nuts and seeds [79], omega‐6 polyunsaturated fatty acids [80], seafood omega‐3 fatty acids [81], vegetables [82], and low whole grain intake [83]. Physical inactivity was quantified in total metabolic equivalent (MET) minutes per week and defined as the objectively measured average weekly physical activity (including work, home, transport‐related, and recreational activities) totaling less than 3600–4400 MET [84]. Human development index (HDI) and gross domestic product (GDP) per capita data of each single country were provided by the United Nations (UN) and the World Bank [85, 86], respectively. The majority of data collected by GBD were gathered by the GBD collaborator Network. For high‐income countries, most of the datasets they incorporated were extracted from the local government databases, including the United States, United Kingdom, Canada, and so forth. Most of the GBD results were accessed via the GBD Results Tool.

### 5.2 Statistical Analysis

For global incidence of HNC, only GLOBOCAN database was used. And for the trend analysis, only C15 plus database was used. These two analyses do not involve the mixed use of different database. As for risk factor analysis, we used dataset form GLOBOCAN and GBD. To deal with the differences in the results, we added log transformed results on risk factor of the HNC.

A multivariable linear regression analysis by sex and age was adopted to evaluate the relationships between HNC incidence and risk factors (including HDI, GDP per capita, different lifestyles, and other risk factors) for each targeted geographical location. We employed various strategies to control for key confounding variables, namely age and sex, in our analysis. First, the outcome variable of incidence was age‐standardized using the WHO standard population, ensuring comparability across different countries. Second, we performed separate analyses for both sexes (male and female) and age categories (young population: 15–49 years, old population: 50–74 years). The regression produced *β* and matching 95% CI. *β* estimates represent the proportional change in the outcome variable (ASR of incidence) for each unit increase in the predictor variable. For risk factors’ associations with HNC, log transformation results were performed to minimize the issue that the beta coefficient can be interpreted as the change in incidence associated with one percent increase of a certain risk factor. All CIs are shown at the 95% level; the result was considered statistically significant when the *p* value was less than 0.05. Variance inflation factor (VIF) values were included in another multivariable linear regression analysis to rule out multicollinearity among variables. The interpretation of the VIF was as follows, VIF = 1: No correlation between the independent variable and other variables; 1 < VIF < 5: Moderate correlation, generally acceptable; VIF ≥ 5: High correlation, indicates potential multicollinearity issues; VIF > 10: Serious multicollinearity, likely requires attention.

In the risk factor association analysis, the GLOBOCAN 2022 for outcome data and GBD 2021 for risk factor exposures were utilized, as GBD 2022 risk factor data were unavailable at the time of analysis. While this introduces a 1‐year temporal mismatch, we expect the impact to be minimal. To address potential time‐lag effects (assuming a 10‐year latency period based on prior literature), we performed a sensitivity analysis using GLOBOCAN 2022 outcomes paired with 2012 GBD risk factor data. The sensitive analysis was univariable and the sensitivity analysis were performed through excluding 38 countries without national cancer registries and estimates were based on modelling from neighboring countries.

Implementing trend analysis by utilizing the SEER Program's (National Cancer Institute of the United States) joinpoint regression analysis software. The Average Annual Percentage Change (AAPC) was applied to calculate the temporal trend of HNC incidence [87]. In the cancer epidemiology study, the most recent 10‐year period data were used, following standard practice. Also, the incidence data were transformed logarithmically, the associated standard errors were computed. Then, a variety of demographical groups were employed to calculate the AAPC and the 95% CI. The AAPC indicates the temporal trends of HNC; a positive AAPC reflects a rising trend and vice versa. The 95% CI was used to evaluate the accuracy of trend estimations, for instance, an interval that overlaps with 0 means a consistent pattern with no noticeable rise or drop. Trend analysis on 41 countries were performed to access the trend of HNC during the period of 2003 to 2012. The selection of countries involved the availability, coverage and quality of data during the examined period and the site‐specific proportions of HNC (C76.0) incidence.

## Author Contributions

M.C.S.W., C.C.Z., and J.H. conceptualized and supervised the study. S.C.C. was responsible for data curation and formal analysis. J.H., S.H.C., M.C., and S.C.C. drafted the manuscript. J.Y., L.Z. C.C.Z., W.X., Z.J.Z., Z.C., J.Y.K.C., and M.C.S.W. reviewed and revised the manuscript. All authors have read and approved the final manuscript.

## Funding

This study was supported by internal funding from The Chinese University of Hong Kong.

## Ethics Statement

This study was approved by the Survey and Behavioural Research Ethics Committee, The Chinese University of Hong Kong (No. SBRE‐20‐332).

## Conflicts of Interest

The authors declare no conflicts of interest.

## Supporting information




**Supporting File 1**: mco270467‐sup‐0001‐SuppMat.docx

## Data Availability

The datasets used and/or analyzed during the current study are available from the corresponding author on reasonable request.
