# Supplementary material for: Incidence, Risk Factors, and Epidemiological Trends of Head and Neck Cancer: A Global Analysis
Source: MedComm (2020). 2025 Nov 14;6(11):e70467. doi: 10.1002/mco2.70467 (PMC12616870; doi:10.1002/mco2.70467)
Supplement: Supplementary file 1 — Supporting File 1: mco270467‐sup‐0001‐SuppMat.docx [file MCO2-6-e70467-s001.docx]

**Supplementary Legends**

**Table S1.** Global incidence of head and neck cancer by regions in 2022

**Table S2.** Global incidence of head and neck cancer by countries in 2022

**Table S3.** Result of risk factors associations with head and neck cancer

**Table S4.** Associations of risk factors with head and neck incidence by sex and age group adjusted for multicollinearity

**Table S5.** Sensitivity analysis on associations of risk factors with head and neck incidence by time

**Table S6.** Sensitivity analysis on associations of risk factors with head and neck incidence by data quality

**Table S7.** Results of Joinpoint regression for trend analysis

**Figure S1.** Plots of Joinpoint regression for trend analysis

**Table S1.** Global incidence of head and neck cancer by regions in 2022

|  | **Both sexes** |  | **Females** |  | **Males** |  | **Old** |  | **Young** |  |
| --- | --- | --- | --- | --- | --- | --- | --- | --- | --- | --- |
| **Region** | **Number** | **ASR** | **Number** | **ASR** | **Number** | **ASR** | **Number** | **ASR** | **Number** | **ASR** |
| Total | 947211 | 9.8 | 236750 | 4.6 | 710461 | 15.3 | 606375 | 36.1 | 185837 | 4.6 |
| Northern America | 70677 | 10.7 | 18851 | 5.5 | 51826 | 16.3 | 48459 | 42.8 | 6865 | 3.7 |
| Eastern Asia | 178879 | 6.4 | 44204 | 3.1 | 134675 | 9.8 | 116483 | 22.9 | 28223 | 3.1 |
| Eastern Africa | 13376 | 5.1 | 4610 | 3.2 | 8766 | 7.5 | 6883 | 17.5 | 4313 | 2.3 |
| Middle Africa | 5021 | 5.0 | 1786 | 3.4 | 3235 | 6.8 | 2812 | 18.2 | 1500 | 2.1 |
| Northern Africa | 14522 | 6.3 | 4272 | 3.5 | 10250 | 9.4 | 8636 | 22.3 | 3673 | 3.0 |
| Southern Africa | 4147 | 7.0 | 1209 | 3.6 | 2938 | 11.6 | 2812 | 27.7 | 733 | 2.1 |
| Western Africa | 9259 | 4.0 | 3591 | 2.9 | 5668 | 5.2 | 5075 | 13.9 | 2968 | 1.7 |
| Caribbean | 6547 | 10.9 | 1332 | 3.9 | 5215 | 18.8 | 4419 | 43.9 | 700 | 3.2 |
| Central America | 5741 | 2.9 | 2037 | 1.8 | 3704 | 4.1 | 3164 | 9.9 | 920 | 0.95 |
| South-Eastern Asia | 82857 | 10.8 | 21903 | 5.4 | 60954 | 16.8 | 51364 | 37.6 | 20786 | 5.5 |
| South Central Asia | 326700 | 16.2 | 77356 | 7.7 | 249344 | 24.8 | 202559 | 58.8 | 86668 | 8.0 |
| Western Asia | 17053 | 6.3 | 4250 | 3.0 | 12803 | 9.6 | 10608 | 23.5 | 3839 | 2.4 |
| Eastern Europe | 65004 | 12.9 | 12637 | 4.2 | 52367 | 24.1 | 47744 | 52.3 | 9335 | 5.4 |
| Northern Europe | 20239 | 10.1 | 5992 | 5.6 | 14247 | 14.9 | 13271 | 40.5 | 1982 | 3.6 |
| Southern Europe | 32514 | 9.8 | 8247 | 4.2 | 24267 | 15.9 | 19888 | 37.9 | 3182 | 3.7 |
| Western Europe | 44153 | 10.7 | 11934 | 5.2 | 32219 | 16.6 | 30437 | 45.9 | 2673 | 2.8 |
| Australia-New Zealand | 5804 | 10.9 | 1580 | 5.5 | 4224 | 16.6 | 3695 | 42.2 | 665 | 4.2 |

| Melanesia | 1596 | 18.5 | 561 | 12.2 | 1035 | 25.3 | 1053 | 70.1 | 427 | 8.5 |
| --- | --- | --- | --- | --- | --- | --- | --- | --- | --- | --- |
| South America | 43022 | 7.6 | 10389 | 3.2 | 32633 | 12.6 | 26929 | 28.4 | 6374 | 2.7 |
| Micronesia | 39 | 6.6 | 3 | 0.90 | 36 | 12.7 | 32 | 28.6 | 6 | 2.5 |
| Polynesia | 61 | 8.0 | 6 | 1.5 | 55 | 14.5 | 52 | 37.7 | 5 | 1.5 |

**Table S2.** Global incidence of head and neck cancer by countries in 2022

|  | **Both sexes** | | **Females** | | **Males** |  | **Old** |  | **Young** |  |
| --- | --- | --- | --- | --- | --- | --- | --- | --- | --- | --- |
| **Country** | |  |  |  |  |  |  |  |  |  |
|  | **Number** | **ASR** | **Number** | **ASR** | **Number** | **ASR** | **Number** | **ASR** | **Number** | **ASR** |
| **World** | 946937 | 9.8 | 236714 | 4.6 | 710223 | 15.3 | 606166 | 36.1 | 185819 | 4.6 |
| **Afghanistan** | 1728 | 7.8 | 531 | 4.7 | 1197 | 11.1 | 892 | 26.9 | 662 | 4.2 |
| **Albania** | 367 | 6.9 | 91 | 3.1 | 276 | 11.0 | 227 | 25.8 | 27 | 2.0 |
| **Algeria** | 3231 | 7.1 | 780 | 3.4 | 2451 | 10.9 | 1824 | 24.3 | 990 | 4.1 |
| **Angola** | 1376 | 8.3 | 530 | 6.1 | 846 | 10.8 | 926 | 35.7 | 317 | 2.5 |
| **Azerbaijan** | 849 | 6.9 | 215 | 3.2 | 634 | 11.3 | 560 | 24.1 | 179 | 3.1 |
| **Argentina** | 3486 | 5.7 | 913 | 2.5 | 2573 | 9.6 | 2309 | 23.5 | 373 | 1.6 |
| **Australia** | 5119 | 11.5 | 1402 | 5.9 | 3717 | 17.4 | 3259 | 44.6 | 596 | 4.4 |
| **Austria** | 1656 | 9.0 | 422 | 4.1 | 1234 | 14.2 | 1114 | 37.3 | 135 | 2.8 |
| **Bahamas** | 18 | 3.6 | 1 | 0.36 | 17 | 7.7 | 13 | 14.4 | 1 | 0.45 |
| **Bahrain** | 49 | 3.9 | 8 | 1.7 | 41 | 5.5 | 31 | 15.1 | 13 | 1.0 |
| **Bangladesh** | 36808 | 23.7 | 8510 | 11.0 | 28298 | 35.9 | 22335 | 88.3 | 9648 | 10.6 |
| **Armenia** | 258 | 5.6 | 40 | 1.3 | 218 | 11.5 | 188 | 23.0 | 23 | 1.5 |
| **Barbados** | 39 | 6.4 | 5 | 1.0 | 34 | 12.7 | 23 | 25.2 | 2 | 1.3 |
| **Belgium** | 2558 | 11.2 | 715 | 5.9 | 1843 | 16.8 | 1776 | 47.0 | 193 | 3.2 |
| **Bhutan** | 72 | 10.3 | 19 | 6.4 | 53 | 13.6 | 49 | 43.5 | 11 | 2.7 |
| **Bolivia** | 392 | 3.3 | 127 | 2.1 | 265 | 4.6 | 234 | 12.3 | 84 | 1.5 |
| **Bosnia Herzegovina** | 557 | 8.8 | 103 | 2.8 | 454 | 15.4 | 411 | 35.8 | 57 | 3.2 |

| **Botswana** | 144 | 8.0 | 23 | 2.1 | 121 | 16.2 | 93 | 31.2 | 33 | 2.8 |
| --- | --- | --- | --- | --- | --- | --- | --- | --- | --- | --- |
| **Brazil** | 29669 | 10.3 | 6418 | 3.9 | 23251 | 17.8 | 18825 | 38.4 | 4415 | 3.6 |
| **Belize** | 7 | 1.8 | 0 | 0.00 | 7 | 3.6 | 4 | 5.9 | 0 | 0.00 |
| **Solomon Islands** | 29 | 5.5 | 11 | 3.7 | 18 | 7.4 | 14 | 16.8 | 12 | 3.9 |
| **Brunei Darussalam** | 38 | 7.5 | 13 | 5.3 | 25 | 9.6 | 25 | 28.5 | 11 | 3.8 |
| **Bulgaria** | 1215 | 8.9 | 254 | 3.4 | 961 | 15.2 | 843 | 36.0 | 144 | 3.5 |
| **Myanmar** | 8180 | 14.0 | 1983 | 6.2 | 6197 | 24.2 | 4945 | 47.2 | 2440 | 8.0 |
| **Burundi** | 301 | 5.2 | 88 | 2.8 | 213 | 8.1 | 170 | 18.3 | 78 | 1.7 |
| **Belarus** | 2622 | 16.4 | 294 | 3.0 | 2328 | 34.4 | 1976 | 65.9 | 400 | 7.4 |
| **Cambodia** | 1304 | 9.3 | 444 | 5.6 | 860 | 14.1 | 799 | 31.9 | 324 | 4.1 |
| **Cameroon** | 1174 | 7.6 | 359 | 4.7 | 815 | 10.6 | 661 | 28.0 | 395 | 3.6 |
| **Canada** | 7220 | 9.6 | 2085 | 5.4 | 5135 | 14.1 | 4692 | 37.4 | 687 | 3.5 |
| **Cape Verde** | 30 | 6.2 | 8 | 2.8 | 22 | 10.6 | 22 | 27.4 | 7 | 2.2 |
| **Central African Republic** | 113 | 4.2 | 42 | 2.8 | 71 | 5.8 | 57 | 13.9 | 41 | 2.4 |
| **Sri Lanka** | 4522 | 14.3 | 893 | 5.0 | 3629 | 25.5 | 3103 | 55.4 | 534 | 4.6 |
| **Chad** | 340 | 3.5 | 203 | 4.2 | 137 | 2.7 | 113 | 9.7 | 191 | 2.9 |
| **Chile** | 927 | 2.9 | 272 | 1.6 | 655 | 4.5 | 535 | 10.5 | 80 | 0.77 |
| **China** | 145603 | 6.2 | 36145 | 3.1 | 109458 | 9.4 | 98126 | 22.3 | 25731 | 3.2 |
| **Colombia** | 3172 | 4.7 | 1076 | 2.9 | 2096 | 6.9 | 1765 | 15.9 | 511 | 1.8 |
| **Comoros** | 12 | 2.4 | 0 | 0.00 | 12 | 5.2 | 10 | 11.2 | 0 | 0.00 |
| **Congo, Republic of** | 63 | 1.9 | 26 | 1.6 | 37 | 2.3 | 43 | 8.1 | 16 | 0.68 |

| **Congo, Democratic Republic of** | 1783 | 3.5 | 585 | 2.1 | 1198 | 5.1 | 907 | 11.5 | 491 | 1.4 |
| --- | --- | --- | --- | --- | --- | --- | --- | --- | --- | --- |
| **Costa Rica** | 301 | 3.9 | 94 | 2.2 | 207 | 5.8 | 171 | 13.9 | 33 | 1.2 |
| **Croatia** | 914 | 10.8 | 167 | 3.2 | 747 | 19.4 | 672 | 47.0 | 66 | 2.9 |
| **Cuba** | 4131 | 18.9 | 726 | 5.8 | 3405 | 33.2 | 2946 | 79.0 | 327 | 5.4 |
| **Cyprus** | 112 | 5.6 | 24 | 2.3 | 88 | 9.1 | 79 | 23.5 | 12 | 1.6 |
| **Czechia** | 2354 | 11.1 | 682 | 5.8 | 1672 | 16.8 | 1597 | 45.6 | 260 | 3.7 |
| **Benin** | 297 | 4.1 | 134 | 3.3 | 163 | 5.0 | 186 | 16.2 | 76 | 1.4 |
| **Denmark** | 1357 | 11.7 | 387 | 6.4 | 970 | 17.2 | 938 | 49.8 | 92 | 3.2 |
| **Dominican Republic** | 807 | 6.8 | 238 | 3.7 | 569 | 10.1 | 513 | 25.7 | 139 | 2.5 |
| **Ecuador** | 612 | 3.0 | 233 | 2.1 | 379 | 4.0 | 325 | 10.2 | 96 | 1.0 |
| **El Salvador** | 212 | 2.6 | 96 | 2.0 | 116 | 3.4 | 80 | 6.9 | 42 | 1.3 |
| **Equatorial Guinea** | 45 | 6.0 | 8 | 2.4 | 37 | 9.0 | 25 | 23.4 | 17 | 2.8 |
| **Ethiopia** | 3052 | 4.0 | 1250 | 3.1 | 1802 | 4.9 | 1376 | 12.6 | 1211 | 2.3 |
| **Eritrea** | 101 | 4.2 | 41 | 3.0 | 60 | 5.4 | 50 | 13.9 | 31 | 2.2 |
| **Estonia** | 260 | 10.7 | 63 | 4.6 | 197 | 18.4 | 194 | 46.5 | 26 | 3.5 |
| **Fiji** | 60 | 6.3 | 27 | 5.6 | 33 | 7.2 | 34 | 19.6 | 22 | 4.8 |
| **Finland** | 893 | 7.2 | 314 | 4.4 | 579 | 10.2 | 545 | 28.1 | 67 | 2.5 |
| **France (metropolitan)** | 17837 | 14.3 | 4570 | 6.7 | 13267 | 22.7 | 12810 | 61.8 | 1353 | 4.1 |
| **French Guyana** | 17 | 5.8 | 0 | 0.00 | 17 | 11.8 | 13 | 25.0 | 3 | 2.0 |
| **French Polynesia** | 41 | 10.8 | 6 | 3.0 | 35 | 18.3 | 34 | 49.5 | 4 | 2.5 |
| **Djibouti** | 26 | 3.1 | 7 | 1.6 | 19 | 4.5 | 18 | 12.7 | 7 | 1.4 |

| **Gabon** | 125 | 8.5 | 33 | 4.6 | 92 | 12.3 | 78 | 33.1 | 32 | 3.0 |
| --- | --- | --- | --- | --- | --- | --- | --- | --- | --- | --- |
| **Georgia** | 688 | 10.0 | 93 | 1.9 | 595 | 20.6 | 489 | 41.1 | 62 | 3.0 |
| **The Republic of the Gambia** | 13 | 0.99 | 2 | 0.28 | 11 | 1.8 | 6 | 3.0 | 6 | 0.75 |
| **Gaza Strip and West Bank** | 158 | 5.0 | 28 | 1.7 | 130 | 8.5 | 102 | 19.9 | 36 | 1.7 |
| **Germany** | 17256 | 8.9 | 4652 | 4.2 | 12604 | 14.0 | 11518 | 38.9 | 692 | 1.8 |
| **Ghana** | 884 | 3.9 | 300 | 2.5 | 584 | 5.5 | 467 | 12.6 | 307 | 2.1 |
| **Greece** | 1948 | 8.1 | 431 | 2.9 | 1517 | 13.9 | 1088 | 30.8 | 179 | 3.0 |
| **France, Guadeloupe** | 74 | 10.1 | 6 | 1.2 | 68 | 21.6 | 57 | 42.1 | 8 | 4.2 |
| **Guam** | 14 | 6.3 | 1 | 0.82 | 13 | 12.1 | 11 | 26.4 | 2 | 2.5 |
| **Guatemala** | 313 | 2.1 | 145 | 1.7 | 168 | 2.6 | 140 | 6.6 | 63 | 0.71 |
| **Guinea** | 184 | 2.5 | 73 | 1.7 | 111 | 3.6 | 84 | 7.3 | 69 | 1.3 |
| **Guyana** | 27 | 3.2 | 5 | 0.97 | 22 | 5.5 | 21 | 14.2 | 2 | 0.55 |
| **Haiti** | 492 | 5.1 | 166 | 3.0 | 326 | 7.5 | 221 | 14.5 | 132 | 2.4 |
| **Honduras** | 438 | 5.4 | 107 | 2.3 | 331 | 9.1 | 257 | 20.3 | 60 | 1.2 |
| **Hungary** | 3425 | 19.4 | 761 | 7.5 | 2664 | 33.6 | 2528 | 82.6 | 439 | 7.0 |
| **Iceland** | 32 | 4.7 | 8 | 1.7 | 24 | 7.7 | 19 | 18.0 | 2 | 1.1 |
| **India** | 247924 | 17.2 | 56996 | 8.0 | 190928 | 26.4 | 155122 | 62.2 | 65133 | 8.6 |
| **Indonesia** | 33603 | 11.1 | 8538 | 5.5 | 25065 | 17.1 | 20497 | 38.4 | 9409 | 6.0 |
| **Iran (Islamic Republic of)** | 4851 | 5.3 | 1392 | 3.1 | 3459 | 7.6 | 3187 | 20.2 | 925 | 1.8 |
| **Iraq** | 1195 | 4.8 | 407 | 3.0 | 788 | 7.0 | 720 | 17.9 | 290 | 1.5 |
| **Ireland** | 813 | 9.7 | 191 | 4.5 | 622 | 15.2 | 542 | 39.5 | 86 | 2.9 |

| **Israel** | 621 | 5.0 | 213 | 3.0 | 408 | 7.2 | 353 | 18.3 | 83 | 1.9 |
| --- | --- | --- | --- | --- | --- | --- | --- | --- | --- | --- |
| **Italy** | 13157 | 9.3 | 3943 | 4.7 | 9214 | 14.3 | 7381 | 33.7 | 1302 | 4.0 |
| **Côte d'Ivoire** | 615 | 3.8 | 242 | 3.1 | 373 | 4.4 | 257 | 10.6 | 279 | 2.5 |
| **Jamaica** | 158 | 4.2 | 45 | 2.2 | 113 | 6.3 | 95 | 15.1 | 24 | 1.5 |
| **Japan** | 26319 | 7.8 | 6561 | 3.2 | 19758 | 12.7 | 13661 | 29.8 | 1657 | 2.6 |
| **Kazakhstan** | 1597 | 7.1 | 391 | 3.1 | 1206 | 12.8 | 1194 | 29.8 | 213 | 2.2 |
| **Jordan** | 497 | 6.2 | 115 | 2.8 | 382 | 9.8 | 281 | 22.0 | 156 | 3.1 |
| **Kenya** | 2446 | 7.8 | 932 | 5.6 | 1514 | 10.5 | 1421 | 29.6 | 801 | 3.2 |
| **Korea (Democratic People Republic of)** | 1552 | 4.2 | 392 | 1.9 | 1160 | 7.1 | 1079 | 16.3 | 223 | 1.6 |
| **Republic of Korea** | 5269 | 5.1 | 1058 | 2.1 | 4211 | 8.5 | 3528 | 19.5 | 588 | 2.0 |
| **Kuwait** | 189 | 4.5 | 48 | 3.2 | 141 | 5.3 | 130 | 17.4 | 48 | 1.3 |
| **Kyrgyzstan** | 331 | 5.8 | 100 | 3.0 | 231 | 10.0 | 220 | 21.2 | 60 | 1.9 |
| **Lao People's Democratic Republic** | 649 | 11.0 | 226 | 7.5 | 423 | 14.7 | 358 | 36.9 | 210 | 5.7 |
| **Lebanon** | 339 | 4.4 | 109 | 2.9 | 230 | 5.9 | 219 | 16.2 | 67 | 1.9 |
| **Lesotho** | 47 | 3.0 | 9 | 0.94 | 38 | 5.8 | 34 | 12.5 | 8 | 0.86 |
| **Latvia** | 433 | 12.2 | 78 | 3.6 | 355 | 23.8 | 311 | 51.1 | 46 | 4.6 |
| **Liberia** | 87 | 2.6 | 29 | 1.7 | 58 | 3.6 | 44 | 8.5 | 34 | 1.5 |
| **Libya** | 417 | 6.7 | 123 | 3.6 | 294 | 10.2 | 253 | 24.9 | 106 | 2.5 |
| **Lithuania** | 733 | 14.4 | 98 | 3.0 | 635 | 29.2 | 551 | 60.4 | 75 | 5.4 |
| **Luxembourg** | 67 | 5.9 | 20 | 3.5 | 47 | 8.2 | 49 | 26.1 | 4 | 1.0 |
| **Madagascar** | 1016 | 5.9 | 224 | 2.3 | 792 | 10.0 | 518 | 18.8 | 293 | 2.4 |

| **Malawi** | 417 | 3.6 | 168 | 2.8 | 249 | 4.8 | 192 | 12.5 | 160 | 1.9 |
| --- | --- | --- | --- | --- | --- | --- | --- | --- | --- | --- |
| **Malaysia** | 4127 | 11.3 | 1100 | 6.0 | 3027 | 16.5 | 2445 | 39.0 | 1092 | 5.8 |
| **Maldives** | 48 | 10.3 | 7 | 3.5 | 41 | 16.0 | 25 | 38.8 | 22 | 6.3 |
| **Mali** | 481 | 4.6 | 253 | 4.5 | 228 | 4.8 | 242 | 15.9 | 171 | 2.2 |
| **Malta** | 73 | 7.6 | 24 | 4.7 | 49 | 10.5 | 44 | 28.9 | 7 | 2.8 |
| **France, Martinique** | 45 | 5.2 | 3 | 0.45 | 42 | 11.3 | 34 | 23.5 | 2 | 1.1 |
| **Mauritania** | 112 | 3.7 | 45 | 2.8 | 67 | 4.7 | 64 | 13.5 | 37 | 1.7 |
| **Mauritius** | 137 | 6.6 | 36 | 3.2 | 101 | 10.5 | 99 | 26.4 | 18 | 2.4 |
| **Mexico** | 4076 | 2.7 | 1474 | 1.8 | 2602 | 3.8 | 2305 | 9.5 | 647 | 0.92 |
| **Mongolia** | 136 | 4.7 | 48 | 3.0 | 88 | 6.7 | 89 | 17.1 | 24 | 1.3 |
| **Moldova** | 992 | 16.1 | 61 | 1.5 | 931 | 34.5 | 751 | 65.2 | 178 | 7.3 |
| **Montenegro** | 100 | 8.4 | 36 | 5.0 | 64 | 12.2 | 65 | 31.2 | 11 | 3.2 |
| **Morocco** | 3293 | 7.7 | 908 | 4.1 | 2385 | 11.7 | 2080 | 27.2 | 739 | 3.8 |
| **Mozambique** | 541 | 2.6 | 177 | 1.3 | 364 | 4.6 | 200 | 7.7 | 248 | 1.9 |
| **Oman** | 179 | 4.3 | 25 | 1.8 | 154 | 5.8 | 84 | 15.4 | 87 | 2.5 |
| **Namibia** | 196 | 12.0 | 64 | 6.6 | 132 | 20.1 | 124 | 46.4 | 45 | 4.0 |
| **Nepal** | 1768 | 6.7 | 478 | 3.2 | 1290 | 10.9 | 1123 | 24.9 | 317 | 2.2 |
| **The Netherlands** | 3163 | 8.5 | 1129 | 5.8 | 2034 | 11.3 | 2071 | 34.4 | 200 | 2.4 |
| **New Caledonia** | 43 | 10.7 | 6 | 2.9 | 37 | 19.0 | 33 | 48.2 | 3 | 1.8 |
| **Vanuatu** | 10 | 4.0 | 6 | 4.9 | 4 | 3.2 | 4 | 9.3 | 6 | 4.8 |
| **New Zealand** | 685 | 7.8 | 178 | 3.6 | 507 | 12.3 | 436 | 29.9 | 69 | 2.9 |

| **Nicaragua** | 207 | 3.3 | 71 | 2.0 | 136 | 5.0 | 111 | 11.2 | 54 | 1.5 |
| --- | --- | --- | --- | --- | --- | --- | --- | --- | --- | --- |
| **Niger** | 409 | 3.3 | 170 | 2.4 | 239 | 4.3 | 194 | 10.2 | 138 | 1.6 |
| **Nigeria** | 5035 | 4.4 | 1867 | 3.2 | 3168 | 5.7 | 2839 | 15.4 | 1484 | 1.6 |
| **Norway** | 873 | 8.0 | 318 | 5.6 | 555 | 10.5 | 528 | 31.2 | 69 | 2.4 |
| **Pakistan** | 24652 | 14.3 | 7283 | 8.6 | 17369 | 19.8 | 13855 | 49.7 | 8469 | 8.2 |
| **Panama** | 187 | 3.3 | 50 | 1.7 | 137 | 5.1 | 96 | 11.4 | 21 | 0.89 |
| **Papua New Guinea** | 1454 | 22.2 | 511 | 14.6 | 943 | 30.4 | 968 | 84.4 | 384 | 9.7 |
| **Paraguay** | 475 | 7.1 | 46 | 1.2 | 429 | 12.9 | 314 | 27.8 | 92 | 2.7 |
| **Peru** | 1596 | 3.8 | 700 | 3.4 | 896 | 4.4 | 815 | 12.2 | 361 | 2.0 |
| **Philippines** | 9008 | 8.9 | 2727 | 4.9 | 6281 | 13.5 | 5694 | 32.6 | 2052 | 3.7 |
| **Poland** | 10605 | 15.2 | 2413 | 5.7 | 8192 | 26.0 | 7544 | 61.9 | 1311 | 5.8 |
| **Portugal** | 3194 | 16.0 | 544 | 4.0 | 2650 | 29.6 | 1986 | 57.1 | 545 | 9.3 |
| **Guinea-Bissau** | 18 | 1.8 | 7 | 1.3 | 11 | 2.4 | 15 | 8.7 | 3 | 0.42 |
| **Timor-Leste** | 67 | 6.9 | 20 | 4.0 | 47 | 10.0 | 41 | 26.0 | 20 | 3.6 |
| **Puerto Rico** | 475 | 7.7 | 77 | 1.8 | 398 | 15.1 | 301 | 31.1 | 36 | 2.3 |
| **Qatar** | 80 | 3.5 | 10 | 2.4 | 70 | 3.6 | 46 | 13.1 | 31 | 1.4 |
| **France, La Réunion** | 186 | 13.4 | 17 | 2.2 | 169 | 26.0 | 140 | 54.8 | 26 | 5.6 |
| **Romania** | 6665 | 19.7 | 845 | 4.3 | 5820 | 37.0 | 4783 | 78.0 | 1049 | 9.1 |
| **Russian Federation** | 26149 | 10.7 | 5748 | 4.0 | 20401 | 20.0 | 19508 | 43.5 | 3890 | 4.5 |
| **Rwanda** | 257 | 3.1 | 49 | 1.0 | 208 | 5.5 | 166 | 12.5 | 67 | 1.1 |
| **Saint Lucia** | 16 | 6.3 | 2 | 1.5 | 14 | 11.5 | 13 | 28.4 | 2 | 1.9 |

| **Sao Tome and Principe** | 2 | 1.6 | 0 | 0.00 | 2 | 3.3 | 2 | 8.7 | 0 | 0.00 |
| --- | --- | --- | --- | --- | --- | --- | --- | --- | --- | --- |
| **Saudi Arabia** | 1409 | 4.3 | 399 | 3.2 | 1010 | 5.0 | 748 | 14.7 | 547 | 2.2 |
| **Senegal** | 348 | 3.5 | 159 | 2.8 | 189 | 4.4 | 185 | 12.1 | 115 | 1.6 |
| **Serbia** | 1817 | 10.9 | 353 | 3.4 | 1464 | 19.3 | 1280 | 45.2 | 155 | 3.2 |
| **Sierra Leone** | 2 | 0.04 | 1 | 0.03 | 1 | 0.04 | 1 | 0.11 | 1 | 0.03 |
| **Singapore** | 1157 | 10.9 | 264 | 5.4 | 893 | 16.1 | 790 | 37.8 | 230 | 6.2 |
| **Slovakia** | 1717 | 18.2 | 278 | 5.1 | 1439 | 32.9 | 1227 | 72.2 | 303 | 8.6 |
| **Viet Nam** | 13739 | 11.4 | 3166 | 4.8 | 10573 | 18.9 | 8908 | 41.6 | 3271 | 5.7 |
| **Slovenia** | 462 | 11.0 | 90 | 3.9 | 372 | 18.2 | 339 | 46.9 | 43 | 3.6 |
| **Somalia** | 529 | 6.1 | 199 | 4.4 | 330 | 7.9 | 294 | 21.7 | 160 | 2.7 |
| **South Africa** | 3748 | 7.0 | 1110 | 3.7 | 2638 | 11.5 | 2555 | 27.7 | 643 | 2.1 |
| **Zimbabwe** | 335 | 4.1 | 119 | 2.3 | 216 | 6.7 | 200 | 16.0 | 91 | 1.4 |
| **Spain** | 9743 | 9.7 | 2428 | 4.3 | 7315 | 15.6 | 6246 | 40.3 | 775 | 2.7 |
| **South Sudan** | 358 | 5.2 | 129 | 3.5 | 229 | 7.1 | 195 | 18.4 | 112 | 2.5 |
| **Sudan** | 1643 | 5.6 | 588 | 3.7 | 1055 | 7.7 | 822 | 17.5 | 529 | 3.0 |
| **Suriname** | 25 | 3.7 | 4 | 1.1 | 21 | 6.7 | 14 | 12.0 | 4 | 1.4 |
| **Eswatini** | 12 | 1.7 | 3 | 0.69 | 9 | 3.0 | 6 | 5.8 | 4 | 0.80 |
| **Sweden** | 1408 | 6.7 | 516 | 4.6 | 892 | 8.8 | 817 | 25.9 | 111 | 2.2 |
| **Switzerland** | 1606 | 8.8 | 426 | 4.0 | 1180 | 13.8 | 1090 | 37.9 | 96 | 2.0 |
| **Syrian Arab Republic** | 854 | 5.6 | 279 | 3.3 | 575 | 8.1 | 516 | 20.4 | 181 | 1.8 |
| **Tajikistan** | 165 | 2.6 | 80 | 2.2 | 85 | 2.9 | 85 | 7.7 | 50 | 1.1 |

| **Thailand** | 10985 | 9.2 | 3422 | 5.2 | 7563 | 13.8 | 6862 | 31.6 | 1727 | 4.3 |
| --- | --- | --- | --- | --- | --- | --- | --- | --- | --- | --- |
| **Togo** | 278 | 5.5 | 92 | 3.3 | 186 | 7.9 | 162 | 20.7 | 94 | 2.5 |
| **Trinidad and Tobago** | 107 | 5.1 | 31 | 3.1 | 76 | 7.5 | 69 | 19.1 | 14 | 1.7 |
| **United Arab Emirates** | 211 | 4.1 | 56 | 3.6 | 155 | 4.4 | 154 | 16.3 | 39 | 0.50 |
| **Tunisia** | 1319 | 8.7 | 320 | 4.1 | 999 | 13.9 | 836 | 30.8 | 293 | 4.4 |
| **Türkiye** | 8144 | 7.8 | 1727 | 3.0 | 6417 | 13.4 | 5396 | 29.9 | 1469 | 3.1 |
| **Turkmenistan** | 364 | 6.6 | 100 | 3.3 | 264 | 10.8 | 232 | 24.1 | 92 | 2.9 |
| **Uganda** | 1511 | 7.2 | 475 | 3.9 | 1036 | 11.6 | 746 | 24.1 | 510 | 3.0 |
| **Ukraine** | 9260 | 12.5 | 1301 | 2.8 | 7959 | 25.6 | 6987 | 51.8 | 1361 | 5.2 |
| **North Macedonia** | 230 | 6.3 | 48 | 2.3 | 182 | 10.5 | 179 | 27.6 | 18 | 1.5 |
| **Egypt** | 4598 | 5.2 | 1553 | 3.3 | 3045 | 7.4 | 2805 | 18.6 | 1011 | 2.0 |
| **United Kingdom** | 13385 | 10.7 | 4009 | 6.1 | 9376 | 15.5 | 8787 | 42.5 | 1406 | 4.0 |
| **Tanzania, United Republic of** | 1782 | 6.1 | 567 | 3.3 | 1215 | 9.8 | 921 | 18.6 | 369 | 1.4 |
| **United States of America** | 63440 | 10.8 | 16765 | 5.5 | 46675 | 16.6 | 43751 | 43.5 | 6178 | 3.7 |
| **Burkina Faso** | 466 | 4.1 | 209 | 3.6 | 257 | 4.6 | 307 | 17.7 | 147 | 1.8 |
| **Uruguay** | 496 | 8.8 | 109 | 3.1 | 387 | 15.6 | 332 | 37.2 | 42 | 2.3 |
| **Uzbekistan** | 1870 | 5.9 | 576 | 3.4 | 1294 | 8.8 | 1137 | 20.5 | 532 | 2.9 |
| **Venezuela** | 2128 | 6.3 | 486 | 2.7 | 1642 | 10.6 | 1427 | 24.4 | 311 | 2.1 |
| **Samoa** | 10 | 5.8 | 0 | 0.00 | 10 | 11.8 | 8 | 26.0 | 1 | 1.3 |
| **Yemen** | 1219 | 6.5 | 452 | 4.7 | 767 | 8.7 | 518 | 19.4 | 519 | 3.7 |
| **Zambia** | 369 | 4.1 | 132 | 2.4 | 237 | 6.3 | 167 | 13.4 | 131 | 1.8 |

**Table S3.** Result of risk factors associations with head and neck cancer

| **Outcome** | **Risk factor** | **Overall** | | | |
| --- | --- | --- | --- | --- | --- |
|  |  | ***β*** | ***95% CI*** | | ***P*** |
|  | HDI | 0.048 | -0.018 | 0.114 | 0.155 |
|  | GDP per capita | -0.008 | -0.060 | 0.044 | 0.759 |
|  | Smoking | 0.037 | 0.020 | 0.054 | **<0.001*** |
|  | Alcohol drinking | 0.033 | 0.014 | 0.052 | **<0.001*** |
| **All Sexes and ages** | Dietary | 0.012 | 0.003 | 0.021 | **0.010** |
|  | Physical inactivity | 0.007 | -0.026 | 0.039 | 0.688 |
|  | Obesity | 0.003 | -0.006 | 0.012 | 0.510 |
|  | Hypertension | 0.006 | -0.006 | 0.017 | 0.345 |
|  | Diabetes | 0.020 | 0.002 | 0.039 | **0.032** |
|  | Lipid | 0.016 | 0.008 | 0.024 | **<0.001*** |
|  |  |  |  |  |  |
|  | HDI | 0.063 | -0.010 | 0.136 | 0.088 |
|  | GDP per capita | -0.011 | -0.068 | 0.046 | 0.706 |
|  | Smoking | 0.043 | 0.024 | 0.062 | **<0.001*** |
|  | Alcohol drinking | 0.044 | 0.023 | 0.064 | **<0.001*** |
| **Male** | Dietary | 0.012 | 0.003 | 0.022 | 0.014 |
|  | Physical inactivity | 0.002 | -0.034 | 0.038 | 0.915 |
|  | Obesity | 0.005 | -0.005 | 0.015 | 0.324 |
|  | Hypertension | 0.010 | -0.003 | 0.023 | 0.126 |
|  | Diabetes | 0.023 | 0.003 | 0.044 | **0.027** |
|  | Lipid | 0.018 | 0.009 | 0.027 | **<0.001*** |
|  |  |  |  |  |  |
|  | HDI | 0.007 | -0.058 | 0.073 | 0.826 |
|  | GDP per capita | -0.010 | -0.060 | 0.041 | 0.705 |
|  | Smoking | 0.018 | 0.000 | 0.035 | **0.049** |
|  | Alcohol drinking | 0.006 | -0.012 | 0.025 | 0.502 |
| **Female** | Dietary | 0.008 | -0.001 | 0.017 | 0.077 |
|  | Physical inactivity | 0.008 | -0.024 | 0.040 | 0.619 |
|  | Obesity | -0.004 | -0.013 | 0.005 | 0.440 |
|  | Hypertension | -0.005 | -0.016 | 0.007 | 0.432 |
|  | Diabetes | 0.007 | -0.012 | 0.025 | 0.480 |
|  | Lipid | 0.009 | 0.001 | 0.017 | **0.038** |
|  |  |  |  |  |  |
|  | HDI | 0.017 | -0.051 | 0.086 | 0.622 |
|  | GDP per capita | -0.027 | -0.081 | 0.026 | 0.315 |
|  | Smoking | 0.025 | 0.007 | 0.044 | **0.008** |
|  | Alcohol drinking | 0.018 | -0.002 | 0.038 | 0.082 |
| **Young** | Dietary | 0.012 | 0.002 | 0.021 | **0.015** |
|  | Physical inactivity | -0.011 | -0.045 | 0.023 | 0.531 |
|  | Obesity | -0.004 | -0.014 | 0.006 | 0.400 |
|  | Hypertension | 0.002 | -0.010 | 0.014 | 0.757 |
|  | Diabetes | 0.010 | -0.010 | 0.030 | 0.308 |
|  | Lipid | 0.011 | 0.002 | 0.020 | **0.017** |
|  |  |  |  |  |  |
|  | HDI | 0.058 | -0.012 | 0.129 | 0.106 |
|  | GDP per capita | -0.003 | -0.058 | 0.053 | 0.928 |
|  | Smoking | 0.043 | 0.024 | 0.061 | **<0.001*** |
|  | Alcohol drinking | 0.040 | 0.020 | 0.060 | **<0.001*** |
| **Old** | Dietary | 0.012 | 0.003 | 0.022 | **0.013** |
|  | Physical inactivity | 0.007 | -0.028 | 0.042 | 0.685 |
|  | Obesity | 0.005 | -0.005 | 0.015 | 0.360 |
|  | Hypertension | 0.008 | -0.005 | 0.021 | 0.225 |
|  | Diabetes | 0.021 | 0.001 | 0.041 | **0.043** |
|  | Lipid | 0.017 | 0.008 | 0.026 | **<0.001*** |

The analysis was conducted using univariable linear regression model at a country level.

*β*, beta coefficient. The beta coefficient can be interpreted as the change in incidence or mortality associated

with one percent increase of a certain risk factor.

CI, confidence interval; ASR, age-standardized rate (log transformed); HDI, human development index;

GDP, gross domestic products.

* *p* values less than 0.05.

**Table S4.** **Associations of risk factors with head and neck incidence by sex and age group adjusted for multicollinearity**

| **Outcome** | **Risk factor** | **Overall** | | | | **VIF** |
| --- | --- | --- | --- | --- | --- | --- |
|  |  | ***β*** | ***95% CI*** | | ***p-value*** |  |
|  | (Intercept) | 1.459 | 0.660 | 2.257 | **<0.001** | NA |
|  | HDI | -0.016 | -0.130 | 0.098 | 0.786 | 3.11 |
|  | GDP per capita | -0.083 | -0.157 | -0.010 | **0.026** | 2.31 |
|  | Smoking | 0.029 | 0.001 | 0.056 | **0.041** | 2.52 |
|  | Alcohol drinking | 0.026 | -0.004 | 0.055 | 0.086 | 2.43 |
| **All Sexes and ages** | Dietary | 0.003 | -0.008 | 0.014 | 0.608 | 1.62 |
|  | Physical inactivity | 0.014 | -0.031 | 0.059 | 0.543 | 2.04 |
|  | Obesity | -0.007 | -0.020 | 0.006 | 0.264 | 2.06 |
|  | Hypertension | -0.011 | -0.025 | 0.003 | 0.116 | 1.43 |
|  | Diabetes | 0.008 | -0.015 | 0.030 | 0.497 | 1.50 |
|  | Lipid | 0.011 | -0.004 | 0.025 | 0.158 | 3.06 |
|  | (Intercept) | 1.786 | 0.984 | 2.589 | **<0.001** | NA |
|  | HDI | -0.012 | -0.138 | 0.115 | 0.856 | 3.21 |
|  | GDP per capita | -0.083 | -0.163 | -0.002 | **0.045** | 2.33 |
|  | Smoking | 0.020 | 0.0010 | 0.038 | **0.036** | 1.83 |
|  | Alcohol drinking | 0.029 | 0.005 | 0.052 | **0.017** | 2.52 |
| **Male** | Dietary | -0.002 | -0.012 | 0.008 | 0.675 | 1.76 |
|  | Physical inactivity | -0.003 | -0.050 | 0.045 | 0.917 | 1.93 |
|  | Obesity | -0.006 | -0.020 | 0.008 | 0.396 | 2.10 |
|  | Hypertension | -0.008 | -0.022 | 0.007 | 0.294 | 1.49 |
|  | Diabetes | 0.009 | -0.015 | 0.033 | 0.453 | 1.57 |
|  | Lipid | 0.012 | -0.004 | 0.027 | 0.146 | 3.19 |
|  | (Intercept) | 0.962 | 0.082 | 1.842 | **0.032** | NA |
|  | HDI | -0.005 | -0.120 | 0.110 | 0.933 | 3.00 |
|  | GDP per capita | -0.070 | -0.149 | 0.009 | 0.081 | 2.54 |
|  | Smoking | 0.014 | -0.020 | 0.048 | 0.419 | 2.99 |
|  | Alcohol drinking | 0.012 | -0.031 | 0.055 | 0.588 | 2.46 |
| **Female** | Dietary | 0.006 | -0.007 | 0.020 | 0.360 | 1.56 |
|  | Physical inactivity | 0.030 | -0.011 | 0.072 | 0.150 | 1.87 |
|  | Obesity | -0.010 | -0.023 | 0.002 | 0.104 | 1.93 |
|  | Hypertension | -0.013 | -0.027 | <0.001 | 0.053 | 1.31 |
|  | Diabetes | 0.001 | -0.024 | 0.025 | 0.959 | 1.56 |
|  | Lipid | 0.014 | -0.001 | 0.028 | 0.059 | 2.63 |
|  | (Intercept) | 0.645 | -0.224 | 1.514 | 0.144 | NA |
|  | HDI | -0.005 | -0.127 | 0.116 | 0.932 | 3.15 |
|  | GDP per capita | -0.075 | -0.152 | 0.003 | 0.059 | 2.31 |
|  | Smoking | 0.026 | -0.005 | 0.057 | 0.101 | 2.31 |
|  | Alcohol drinking | 0.006 | -0.022 | 0.034 | 0.666 | 2.08 |
| **Young** | Dietary | 0.002 | -0.010 | 0.014 | 0.756 | 1.59 |
|  | Physical inactivity | -0.006 | -0.054 | 0.041 | 0.792 | 1.94 |
|  | Obesity | -0.013 | -0.028 | 0.001 | 0.062 | 2.19 |
|  | Hypertension | -0.015 | -0.035 | 0.004 | 0.113 | 1.11 |
|  | Diabetes | 0.046 | -0.008 | 0.100 | 0.093 | 1.36 |
|  | Lipid | 0.015 | -0.001 | 0.030 | 0.072 | 2.45 |
|  | (Intercept) | 2.651 | 1.733 | 3.569 | **<0.001** | NA |
|  | HDI | -0.012 | -0.133 | 0.109 | 0.843 | 3.03 |
|  | GDP per capita | -0.090 | -0.171 | -0.008 | **0.032** | 2.47 |
|  | Smoking | 0.028 | 0.005 | 0.052 | **0.018** | 2.13 |
|  | Alcohol drinking | 0.030 | <0.001 | 0.061 | 0.053 | 2.09 |
| **Old** | Dietary | 0.005 | -0.007 | 0.016 | 0.413 | 1.83 |
|  | Physical inactivity | 0.022 | -0.026 | 0.070 | 0.362 | 2.23 |
|  | Obesity | -0.004 | -0.015 | 0.007 | 0.479 | 2.11 |
|  | Hypertension | -0.009 | -0.019 | 0.002 | 0.098 | 1.07 |
|  | Diabetes | <0.001 | -0.013 | 0.013 | 0.960 | 1.49 |
|  | Lipid | 0.008 | -0.006 | 0.023 | 0.253 | 2.54 |

The analysis was conducted using multivariable linear regression model at a country level.

β (beta coefficient): Indicates the change in incidence or mortality associated with a one-unit (or one percent) increase in a risk factor. ASR, age-standardized rate (log transformed); CI, confidence interval; VIF, variance inflation factor; Young, age between15-49 years; Old, age between50-74 years; HDI, human development index; GDP, gross domestic products

**Table S5. Sensitivity analysis on associations of risk factors with head and neck incidence by time**

| **Outcome** | **Risk factor** | **Overall** | | | |
| --- | --- | --- | --- | --- | --- |
|  |  | ***β*** | ***95% CI*** | | ***p-value*** |
|  | HDI | 0.149 | 0.087 | 0.211 | **<0.001** |
|  | GDP per capita | 0.072 | 0.020 | 0.123 | **0.007** |
|  | Smoking | 0.028 | 0.017 | 0.039 | **<0.001** |
|  | Alcohol drinking | 0.016 | 0.006 | 0.026 | **0.001** |
| **All Sexes and ages** | Dietary | -0.007 | -0.022 | 0.009 | 0.387 |
|  | Physical inactivity | 0.008 | -0.002 | 0.018 | 0.126 |
|  | Obesity | 0.006 | -0.004 | 0.016 | 0.225 |
|  | Hypertension | 0.000 | -0.011 | 0.010 | 0.969 |
|  | Diabetes | -0.003 | -0.019 | 0.014 | 0.754 |
|  | Lipid | 0.018 | 0.009 | 0.026 | **<0.001** |
|  | HDI | 0.173 | 0.104 | 0.241 | **<0.001** |
|  | GDP per capita | 0.075 | 0.017 | 0.132 | **0.011** |
|  | Smoking | 0.021 | 0.012 | 0.029 | **<0.001** |
|  | Alcohol drinking | 0.025 | 0.012 | 0.039 | **<0.001** |
| **Male** | Dietary | -0.011 | -0.028 | 0.007 | **0.234** |
|  | Physical inactivity | 0.009 | -0.005 | 0.023 | 0.220 |
|  | Obesity | 0.011 | 0.001 | 0.022 | **0.038** |
|  | Hypertension | 0.004 | -0.007 | 0.014 | **0.473** |
|  | Diabetes | 0.000 | -0.019 | 0.018 | 0.959 |
|  | Lipid | 0.019 | 0.010 | 0.027 | **<0.001** |
|  | HDI | 0.073 | 0.011 | 0.136 | **0.022** |
|  | GDP per capita | 0.042 | -0.009 | 0.092 | 0.104 |
|  | Smoking | 0.014 | 0.001 | 0.026 | **0.032** |
|  | Alcohol drinking | 0.006 | -0.002 | 0.014 | 0.123 |
| **Female** | Dietary | 0.004 | -0.010 | 0.018 | 0.603 |
|  | Physical inactivity | 0.000 | -0.008 | 0.009 | 0.933 |
|  | Obesity | -0.006 | -0.016 | 0.004 | 0.220 |
|  | Hypertension | -0.004 | -0.014 | 0.006 | 0.432 |
|  | Diabetes | -0.017 | -0.034 | -0.001 | **0.040** |
|  | Lipid | 0.010 | 0.001 | 0.019 | **0.027** |
|  | HDI | 0.078 | 0.010 | 0.145 | **0.025** |
|  | GDP per capita | 0.028 | -0.027 | 0.083 | 0.313 |
|  | Smoking | 0.015 | 0.006 | 0.025 | **0.002** |
|  | Alcohol drinking | 0.006 | -0.004 | 0.015 | 0.241 |
|  | Dietary | -0.007 | -0.024 | 0.009 | **0.377** |
| **Young** | Physical inactivity | -0.005 | -0.017 | 0.007 | 0.403 |
|  | Obesity | -0.005 | -0.016 | 0.006 | 0.359 |
|  | Hypertension | -0.002 | -0.013 | 0.009 | 0.687 |
|  | Diabetes | -0.009 | -0.025 | 0.008 | 0.289 |
|  | Lipid | 0.011 | 0.002 | 0.020 | **0.019** |
|  | HDI | 0.173 | 0.107 | 0.239 | **<0.001** |
|  | GDP per capita | 0.084 | 0.028 | 0.139 | **0.003** |
|  | Smoking | 0.034 | 0.021 | 0.046 | **<0.001** |
|  | Alcohol drinking | 0.024 | 0.011 | 0.037 | **<0.001** |
|  | Dietary | -0.002 | -0.017 | 0.012 | **0.744** |
| **Old** | Physical inactivity | 0.007 | -0.003 | 0.016 | 0.189 |
|  | Obesity | 0.005 | -0.004 | 0.013 | 0.280 |
|  | Hypertension | -0.002 | -0.013 | 0.009 | 0.665 |
|  | Diabetes | -0.004 | -0.021 | 0.013 | 0.642 |
|  | Lipid | 0.019 | 0.010 | 0.028 | **<0.001** |

This analysis is univariable linear regression model with independent variables in 2012 at a country level. β (beta coefficient): Indicates the change in incidence or mortality associated with a one-unit (or one percent) increase in a risk factor. ASR, age-standardized rate (log transformed); CI, confidence interval; Young, age between 15-49 years; Old, age between 50-74 years; HDI, human development index; GDP, gross domestic product

**Table S6. Sensitivity analysis on associations of risk factors with head and neck incidence by data quality**

| **Outcome** | **Risk factor** | **Overall** | | | |
| --- | --- | --- | --- | --- | --- |
|  |  | ***β*** | ***95% CI*** | | ***p-value*** |
|  | HDI | 0.099 | 0.038 | 0.159 | **0.002** |
|  | GDP per capita | 0.04 | -0.005 | 0.085 | 0.078 |
|  | Smoking | 0.035 | 0.02 | 0.05 | **<0.001** |
|  | Alcohol drinking | 0.033 | 0.016 | 0.05 | **<0.001** |
| **All Sexes and ages** | Dietary | 0.011 | 0.003 | 0.019 | **0.005** |
|  | Physical inactivity | 0.001 | -0.028 | 0.03 | 0.953 |
|  | Obesity | 0.002 | -0.007 | 0.011 | 0.695 |
|  | Hypertension | 0.015 | 0.005 | 0.026 | **0.005** |
|  | Diabetes | 0.01 | -0.008 | 0.028 | 0.268 |
|  | Lipid | 0.017 | 0.01 | 0.025 | **<0.001** |
|  | HDI | 0.121 | 0.053 | 0.188 | **<0.001** |
|  | GDP per capita | 0.04 | -0.011 | 0.09 | 0.122 |
|  | Smoking | 0.031 | 0.018 | 0.043 | **<0.001** |
|  | Alcohol drinking | 0.034 | 0.02 | 0.047 | **<0.001** |
| **Male** | Dietary | 0.011 | 0.004 | 0.017 | **0.002** |
|  | Physical inactivity | -0.015 | -0.048 | 0.017 | 0.358 |
|  | Obesity | 0.004 | -0.007 | 0.014 | 0.482 |
|  | Hypertension | 0.02 | 0.009 | 0.031 | **<0.001** |
|  | Diabetes | 0.014 | -0.005 | 0.033 | 0.154 |
|  | Lipid | 0.019 | 0.01 | 0.027 | **<0.001** |
|  | HDI | 0.045 | -0.018 | 0.109 | 0.16 |
|  | GDP per capita | 0.038 | -0.007 | 0.083 | 0.099 |
|  | Smoking | 0.018 | 0.001 | 0.036 | **0.036** |
|  | Alcohol drinking | 0.022 | -0.003 | 0.046 | 0.084 |
| **Female** | Dietary | 0.007 | -0.002 | 0.017 | 0.136 |
|  | Physical inactivity | 0.013 | -0.014 | 0.041 | 0.326 |
|  | Obesity | -0.005 | -0.014 | 0.004 | 0.254 |
|  | Hypertension | 0.001 | -0.01 | 0.012 | 0.889 |
|  | Diabetes | -0.006 | -0.024 | 0.012 | 0.515 |
|  | Lipid | 0.01 | 0.002 | 0.018 | **0.021** |
|  | HDI | 0.055 | -0.013 | 0.124 | 0.114 |
|  | GDP per capita | 0.014 | -0.037 | 0.064 | 0.589 |
|  | Smoking | 0.028 | 0.009 | 0.048 | **0.005** |
|  | Alcohol drinking | 0.017 | -0.002 | 0.036 | 0.082 |
| **Young** | Dietary | 0.011 | 0.002 | 0.02 | **0.013** |
|  | Physical inactivity | -0.016 | -0.049 | 0.017 | 0.35 |
|  | Obesity | -0.007 | -0.018 | 0.003 | 0.148 |
|  | Hypertension | 0.003 | -0.015 | 0.021 | 0.757 |
|  | Diabetes | 0.005 | -0.046 | 0.055 | 0.856 |
|  | Lipid | 0.014 | 0.004 | 0.025 | **0.008** |
|  | HDI | 0.114 | 0.048 | 0.181 | **<0.001** |
|  | GDP per capita | 0.05 | 0.001 | 0.098 | **0.047** |
|  | Smoking | 0.035 | 0.021 | 0.05 | **<0.001** |
|  | Alcohol drinking | 0.038 | 0.018 | 0.057 | **<0.001** |
| **Old** | Dietary | 0.011 | 0.003 | 0.019 | **0.007** |
|  | Physical inactivity | -0.003 | -0.033 | 0.027 | 0.835 |
|  | Obesity | 0.001 | -0.007 | 0.009 | 0.836 |
|  | Hypertension | 0.006 | -0.004 | 0.015 | 0.255 |
|  | Diabetes | -0.007 | -0.017 | 0.004 | 0.209 |
|  | Lipid | 0.019 | 0.01 | 0.028 | **<0.001** |

This analysis is univariable and sensitivity analysis after excluding 38 countries without national cancer registries and estimates were based on modelling from neighboring countries.

β (beta coefficient): Indicates the change in incidence or mortality associated with a one-unit (or one percent) increase in a risk factor. ASR, age-standardized rate (log transformed); CI, confidence interval; Young, age between15-49 years; Old, age between50-74 years; HDI, human development index; GDP, gross domestic products

**Table S7. Results of Joinpoint regression for trend analysis**

1. Both

| **Region** | **AAPC** | **Lower CI** | **Upper CI** | **p-value** | **Significant** |
| --- | --- | --- | --- | --- | --- |
| ***Asia*** |  |  |  |  |  |
| Bahrain | 5.60 | 1.23 | 9.98 | 0.009 | * |
| China | 7.12 | 3.63 | 9.72 | <0.001 | * |
| India | 8.31 | 4.15 | 11.07 | <0.001 | * |
| Israel | -4.10 | -5.13 | -3.68 | <0.001 | * |
| Japan | -1.03 | -1.99 | -0.22 | 0.013 | * |
| South Korea | -0.42 | -0.73 | -0.12 | 0.006 | * |
| Kuwait | -3.90 | -9.12 | 1.77 | 0.116 |  |
| Philippines | -0.29 | -5.69 | 5.25 | 0.827 |  |
| Qatar | 3.75 | -5.69 | 13.67 | 0.488 |  |
| Thailand | 1.38 | -2.90 | 5.77 | 0.446 |  |
| Turkey | -5.94 | -6.42 | -5.45 | <0.001 | * |
| ***Oceania*** |  |  |  |  |  |
| Australia | 0.13 | -1.38 | 1.40 | 0.894 |  |
| New Zealand | 1.53 | 0.97 | 2.05 | <0.001 | * |
| ***Northern America*** |  |  |  |  |  |
| Canada | 1.22 | 0.39 | 2.04 | 0.005 | * |
| United States | -0.19 | -0.32 | -0.07 | 0.001 | * |
| ***Southern America*** |  |  |  |  |  |
| Argentina | -1.38 | -4.93 | 2.75 | 0.315 |  |
| Chile | 5.34 | 1.56 | 9.24 | 0.004 | * |
| Colombia | 3.61 | -3.44 | 8.97 | 0.316 |  |
| Costa Rica | -2.15 | -3.93 | -0.65 | 0.003 | * |
| Ecuador | -1.76 | -6.27 | 2.97 | 0.372 |  |
| Martinique | -2.19 | -7.09 | 2.93 | 0.342 |  |
| Puerto Rico | -0.80 | -1.90 | 0.59 | 0.221 |  |

| ***Northern Europe*** |  |  |  |  |  |
| --- | --- | --- | --- | --- | --- |
| Denmark | -2.73 | -4.46 | -1.00 | 0.001 | * |
| Estonia | 0.51 | -0.60 | 1.55 | 0.401 |  |
| Iceland | -1.28 | -3.64 | 2.82 | 0.459 |  |
| Ireland | 1.58 | 0.31 | 2.46 | 0.012 | * |
| Latvia | 2.09 | 1.21 | 3.02 | <0.001 | * |
| Lithuania | 0.70 | -0.30 | 1.58 | 0.183 |  |
| Norway | 0.55 | -0.16 | 1.07 | 0.124 |  |
| United Kingdom | 8.25 | 4.66 | 11.45 | <0.001 | * |
| ***Western Europe*** |  |  |  |  |  |
| Austria | -0.15 | -1.94 | 1.63 | 0.815 |  |
| France | -2.66 | -4.68 | -0.61 | 0.014 | * |
| Germany | 3.86 | 0.43 | 7.53 | 0.028 | * |
| Netherlands | -1.10 | -1.49 | -0.73 | <0.001 | * |
| Switzerland | -3.17 | -3.97 | -2.38 | <0.001 | * |
| ***Southern Europe*** |  |  |  |  |  |
| Croatia | -1.33 | -1.69 | -0.99 | <0.001 | * |
| Cyprus | -0.35 | -1.79 | 1.42 | 0.600 |  |
| Italy | 8.36 | 2.49 | 12.00 | 0.004 | * |
| Malta | -3.28 | -4.04 | -2.19 | <0.001 | * |
| Slovenia | -1.38 | -2.42 | -0.42 | 0.006 | * |
| Spain | 10.07 | 3.16 | 14.29 | 0.002 | * |
| ***Eastern Europe*** |  |  |  |  |  |
| Belarus | 2.02 | 1.39 | 2.61 | <0.001 | * |
| Czech | 0.80 | 0.31 | 1.26 | 0.002 | * |
| Poland | 0.08 | -0.96 | 1.07 | 0.962 |  |
| ***Africa*** |  |  |  |  |  |
| Uganda | -1.43 | -6.14 | 3.48 | 0.589 |  |

AAPC, annual percentage change; CI, confidence interval; * p values less than 0·05.

1. Male

| **Region** | **AAPC** | **Lower CI** | **Upper CI** | **p-value** | **Significant** |
| --- | --- | --- | --- | --- | --- |
| ***Asia*** |  |  |  |  |  |
| Bahrain | 4.12 | 0.75 | 7.26 | 0.012 | * |
| China | 7.79 | 4.74 | 10.50 | <0.001 | * |
| India | 8.48 | 4.53 | 11.32 | <0.001 | * |
| Israel | -3.68 | -4.90 | -3.19 | <0.001 | * |
| Japan | -1.59 | -2.56 | -0.77 | <0.001 | * |
| South Korea | -0.90 | -1.09 | -0.72 | <0.001 | * |
| Kuwait | -4.59 | -9.01 | 1.85 | 0.088 |  |
| Philippines | -0.59 | -5.55 | 4.49 | 0.706 |  |
| Qatar | -0.14 | -6.54 | 11.01 | 0.909 |  |
| Thailand | 2.32 | -0.92 | 4.96 | 0.148 |  |
| Turkey | -6.40 | -6.92 | -5.81 | <0.001 | * |
| ***Oceania*** |  |  |  |  |  |
| Australia | 1.01 | -0.33 | 2.20 | 0.148 |  |
| New Zealand | 1.06 | -0.05 | 2.12 | 0.058 |  |
| ***Northern America*** |  |  |  |  |  |
| Canada | 1.31 | 0.34 | 2.25 | 0.009 | * |
| United States | -0.30 | -0.63 | 0.06 | 0.069 |  |
| ***Southern America*** |  |  |  |  |  |
| Argentina | -0.50 | -3.81 | 3.23 | 0.666 |  |
| Chile | 5.90 | 3.34 | 8.23 | <0.001 | * |
| Colombia | 4.22 | 1.53 | 5.63 | 0.004 | * |
| Costa Rica | -2.27 | -3.91 | -1.05 | <0.001 | * |
| Ecuador | -3.18 | -8.10 | 1.88 | 0.188 |  |
| Martinique | -3.05 | -6.11 | 0.09 | 0.054 |  |
| Puerto Rico | -0.65 | -1.60 | 0.46 | 0.229 |  |

| ***Northern Europe*** |  |  |  |  |  |
| --- | --- | --- | --- | --- | --- |
| Denmark | -2.28 | -3.74 | -0.81 | 0.003 | * |
| Estonia | 0.15 | -1.10 | 1.36 | 0.880 |  |
| Iceland | 0.25 | -1.90 | 3.49 | 0.845 |  |
| Ireland | 0.66 | 0.19 | 1.14 | 0.008 | * |
| Latvia | 2.22 | 1.43 | 3.18 | <0.001 | * |
| Lithuania | 0.12 | -0.99 | 1.07 | 0.895 |  |
| Norway | -0.06 | -0.82 | 0.48 | 0.776 |  |
| United Kingdom | 8.31 | 4.12 | 11.77 | <0.001 | * |
| ***Western Europe*** |  |  |  |  |  |
| Austria | -0.14 | -1.79 | 1.51 | 0.827 |  |
| France | -3.28 | -5.48 | -1.09 | 0.006 | * |
| Germany | 3.63 | -0.05 | 7.59 | 0.054 |  |
| Netherlands | -1.35 | -1.73 | -0.98 | <0.001 | * |
| Switzerland | -3.61 | -4.59 | -3.02 | <0.001 | * |
| ***Southern Europe*** |  |  |  |  |  |
| Croatia | -1.56 | -2.23 | -1.11 | <0.001 | * |
| Cyprus | -1.17 | -2.50 | 1.08 | 0.392 |  |
| Italy | 8.67 | 3.86 | 11.78 | <0.001 | * |
| Malta | -3.71 | -5.37 | -2.00 | <0.001 | * |
| Slovenia | -2.09 | -3.13 | -1.11 | <0.001 | * |
| Spain | 9.42 | 2.66 | 13.57 | 0.005 | * |
| ***Eastern Europe*** |  |  |  |  |  |
| Belarus | 1.90 | 1.37 | 2.39 | <0.001 | * |
| Czech | 0.33 | -0.09 | 0.73 | 0.139 |  |
| Poland | -0.50 | -1.16 | 0.13 | 0.108 |  |
| ***Africa*** |  |  |  |  |  |
| Uganda | 0.49 | -4.84 | 6.07 | 0.871 |  |

AAPC, annual percentage change; CI, confidence interval; * p values less than 0·05.

1. Female

| **Region** | **AAPC** | **Lower CI** | **Upper CI** | **p-value** | **Significant** |
| --- | --- | --- | --- | --- | --- |
| ***Asia*** |  |  |  |  |  |
| Bahrain | 14.23 | -7.02 | 39.08 | 0.204 |  |
| China | 6.23 | 4.11 | 7.73 | <0.001 | * |
| India | 6.85 | 2.19 | 10.36 | 0.002 | * |
| Israel | -5.07 | -6.40 | -3.80 | <0.001 | * |
| Japan | 1.11 | -0.92 | 3.07 | 0.309 |  |
| South Korea | 2.19 | 1.12 | 3.22 | <0.001 | * |
| Kuwait | -5.94 | -8.86 | -2.98 | <0.001 | * |
| Philippines | 0.44 | -6.25 | 7.32 | 0.960 |  |
| Qatar | 0.03 | -11.60 | 12.84 | 0.940 |  |
| Thailand | -1.61 | -5.33 | 2.20 | 0.344 |  |
| Turkey | -3.57 | -4.34 | -2.90 | <0.001 | * |
| ***Oceania*** |  |  |  |  |  |
| Australia | -0.59 | -2.34 | 0.92 | 0.411 |  |
| New Zealand | 2.82 | 1.53 | 4.04 | <0.001 | * |
| ***Northern America*** |  |  |  |  |  |
| Canada | 1.02 | 0.09 | 1.92 | 0.029 | * |
| United States | -0.04 | -0.68 | 0.57 | 0.850 |  |
| ***Southern America*** |  |  |  |  |  |
| Argentina | -4.58 | -7.92 | -1.37 | 0.006 | * |
| Chile | 4.10 | -7.90 | 18.20 | 0.553 |  |
| Colombia | 2.97 | -2.03 | 8.76 | 0.226 |  |
| Costa Rica | -0.42 | -3.83 | 2.99 | 0.717 |  |
| Ecuador | 0.65 | -3.57 | 4.69 | 0.767 |  |
| Martinique | 1.84 | -7.73 | 12.13 | 0.695 |  |
| Puerto Rico | -0.05 | -3.65 | 3.58 | 0.927 |  |
| ***Northern Europe*** |  |  |  |  |  |

| Denmark | -4.05 | -5.68 | -2.43 | <0.001 | * |
| --- | --- | --- | --- | --- | --- |
| Estonia | 2.22 | -1.16 | 5.20 | 0.155 |  |
| Iceland | -3.36 | -8.38 | 1.38 | 0.164 |  |
| Ireland | 1.62 | 0.31 | 2.49 | 0.020 | * |
| Latvia | 1.33 | -1.52 | 4.08 | 0.360 |  |
| Lithuania | 6.41 | 4.99 | 7.91 | <0.001 | * |
| Norway | 1.88 | 0.80 | 2.87 | <0.001 | * |
| United Kingdom | 8.08 | 4.68 | 10.76 | <0.001 | * |
| ***Western Europe*** |  |  |  |  |  |
| Austria | 0.00 | -1.86 | 1.85 | 0.937 |  |
| France | -0.11 | -1.25 | 0.99 | 0.797 |  |
| Germany | 4.49 | 1.38 | 7.81 | 0.007 | * |
| Netherlands | -0.37 | -0.92 | 0.08 | 0.088 |  |
| Switzerland | -0.77 | -2.83 | 1.28 | 0.359 |  |
| ***Southern Europe*** |  |  |  |  |  |
| Croatia | 1.13 | -0.29 | 2.39 | 0.098 |  |
| Cyprus | 2.06 | -1.55 | 5.76 | 0.274 |  |
| Italy | 6.75 | 0.27 | 11.06 | 0.038 | * |
| Malta | 0.03 | -5.40 | 5.45 | 0.926 |  |
| Slovenia | 1.48 | -0.45 | 3.33 | 0.143 |  |
| Spain | 13.33 | 7.35 | 17.11 | <0.001 | * |
| ***Eastern Europe*** |  |  |  |  |  |
| Belarus | 3.33 | 1.64 | 4.92 | <0.001 | * |
| Czech | 2.29 | 1.54 | 2.92 | <0.001 | * |
| Poland | 3.04 | -0.93 | 6.87 | 0.146 |  |
| ***Africa*** |  |  |  |  |  |
| Uganda | -5.19 | -10.39 | 0.11 | 0.055 |  |

AAPC, annual percentage change; CI, confidence interval; * p values less than 0·05.

1. Young

| **Region** | **AAPC** | **Lower CI** | **Upper CI** | **p-value** | **Significant** |
| --- | --- | --- | --- | --- | --- |
| ***Asia*** |  |  |  |  |  |
| Bahrain | 1.26 | -10.23 | 13.36 | 0.890 |  |
| China | 10.37 | 3.52 | 17.13 | 0.002 | * |
| India | 15.56 | 8.06 | 22.53 | <0.001 | * |
| Israel | -4.07 | -5.23 | -3.08 | <0.001 | * |
| Japan | 5.22 | 0.57 | 9.71 | 0.026 | * |
| South Korea | 1.76 | 1.31 | 2.05 | <0.001 | * |
| Kuwait | -9.17 | -16.28 | -2.08 | 0.015 | * |
| Philippines | -2.82 | -7.41 | 1.85 | 0.193 |  |
| Qatar | -12.57 | -23.09 | -2.34 | 0.021 | * |
| Thailand | 2.52 | -6.04 | 11.87 | 0.569 |  |
| Turkey | 0.75 | -3.50 | 5.23 | 0.695 |  |
| ***Oceania*** |  |  |  |  |  |
| Australia | -1.84 | -11.81 | 8.89 | 0.739 |  |
| New Zealand | 0.30 | -0.79 | 0.96 | 0.694 |  |
| ***Northern America*** |  |  |  |  |  |
| Canada | 0.12 | -1.26 | 1.46 | 0.839 |  |
| United States | -0.38 | -1.27 | 0.27 | 0.240 |  |
| ***Southern America*** |  |  |  |  |  |
| Argentina | 5.06 | -1.46 | 11.52 | 0.144 |  |
| Chile | 6.28 | -2.44 | 12.30 | 0.160 |  |
| Colombia | 15.47 | 3.23 | 28.67 | 0.013 | * |
| Costa Rica | 3.49 | -0.05 | 6.97 | 0.053 |  |
| Ecuador | -4.42 | -8.22 | -0.50 | 0.034 | * |
| Martinique | -4.31 | -16.23 | 9.00 | 0.386 |  |
| Puerto Rico | -0.10 | -3.79 | 3.54 | 0.876 |  |
| ***Northern Europe*** |  |  |  |  |  |

| Denmark | -10.98 | -19.01 | -2.29 | 0.018 | * |
| --- | --- | --- | --- | --- | --- |
| Estonia | 3.11 | -4.15 | 11.01 | 0.417 |  |
| Iceland | 0.50 | -13.82 | 9.42 | 0.801 |  |
| Ireland | 2.37 | 0.18 | 3.57 | 0.040 | * |
| Latvia | 2.02 | -0.07 | 3.93 | 0.062 |  |
| Lithuania | 7.08 | 5.71 | 8.60 | <0.001 | * |
| Norway | 0.46 | -2.81 | 3.68 | 0.812 |  |
| United Kingdom | 14.38 | 4.50 | 24.62 | 0.003 | * |
| ***Western Europe*** |  |  |  |  |  |
| Austria | -4.36 | -6.89 | -1.82 | <0.001 | * |
| France | 0.32 | -2.42 | 3.34 | 0.890 |  |
| Germany | 13.20 | -0.90 | 28.79 | 0.070 |  |
| Netherlands | -4.44 | -6.01 | -2.52 | <0.001 | * |
| Switzerland | -2.79 | -6.56 | 0.56 | 0.092 |  |
| ***Southern Europe*** |  |  |  |  |  |
| Croatia | -3.32 | -5.31 | -1.27 | 0.001 | * |
| Cyprus | 3.24 | 0.47 | 5.80 | 0.017 | * |
| Italy | 12.23 | 3.95 | 20.62 | 0.005 | * |
| Malta | -7.55 | -15.68 | 1.63 | 0.077 |  |
| Slovenia | -2.17 | -6.00 | 0.27 | 0.071 |  |
| Spain | 10.73 | -1.14 | 23.65 | 0.080 |  |
| ***Eastern Europe*** |  |  |  |  |  |
| Belarus | 4.08 | 2.47 | 5.63 | <0.001 | * |
| Czech | 0.06 | -1.46 | 1.56 | 0.998 |  |
| Poland | -5.18 | -7.69 | -2.50 | 0.003 | * |
| ***Africa*** |  |  |  |  |  |
| Uganda | 2.00 | -1.85 | 5.63 | 0.328 |  |

AAPC, annual percentage change; CI, confidence interval; * p values less than 0·05.

1. Old

| **Region** | **AAPC** | **Lower CI** | **Upper CI** | **p-value** | **Significant** |
| --- | --- | --- | --- | --- | --- |
| ***Asia*** |  |  |  |  |  |
| Bahrain | 3.71 | -4.64 | 12.47 | 0.388 |  |
| China | 0.98 | -4.23 | 3.44 | 0.882 |  |
| India | -1.03 | -7.06 | 3.19 | 0.351 |  |
| Israel | -1.12 | -2.00 | -0.32 | 0.008 | * |
| Japan | -1.41 | -2.98 | -0.09 | 0.035 | * |
| South Korea | -0.93 | -1.29 | -0.59 | <0.001 | * |
| Kuwait | -2.55 | -7.86 | 2.85 | 0.280 |  |
| Philippines | -2.26 | -7.83 | 3.52 | 0.351 |  |
| Qatar | 2.36 | -11.87 | 18.53 | 0.800 |  |
| Thailand | 2.10 | -1.36 | 5.39 | 0.218 |  |
| Turkey | -2.23 | -3.40 | -1.08 | 0.000 | * |
| ***Oceania*** |  |  |  |  |  |
| Australia | 0.59 | 0.07 | 1.07 | 0.026 | * |
| New Zealand | 2.68 | 1.51 | 3.64 | <0.001 | * |
| ***Northern America*** |  |  |  |  |  |
| Canada | 1.13 | 0.30 | 1.92 | 0.007 | * |
| United States | 0.00 | -0.18 | 0.17 | 0.905 |  |
| ***Southern America*** |  |  |  |  |  |
| Argentina | -2.01 | -4.94 | 0.90 | 0.158 |  |
| Chile | 3.91 | -1.45 | 9.12 | 0.164 |  |
| Colombia | -2.13 | -7.56 | 0.10 | 0.060 |  |
| Costa Rica | -2.42 | -4.50 | -0.62 | 0.005 | * |
| Ecuador | 1.86 | -3.87 | 7.74 | 0.541 |  |
| Martinique | -2.56 | -11.43 | 7.03 | 0.452 |  |
| Puerto Rico | -0.82 | -2.47 | 1.19 | 0.368 |  |
| ***Northern Europe*** |  |  |  |  |  |

| Denmark | -0.96 | -1.94 | 0.02 | 0.054 |  |
| --- | --- | --- | --- | --- | --- |
| Estonia | -0.44 | -0.99 | 0.26 | 0.216 |  |
| Iceland | 0.21 | -5.50 | 5.91 | 0.988 |  |
| Ireland | 1.94 | 1.52 | 2.21 | <0.001 | * |
| Latvia | 1.07 | 0.14 | 1.91 | 0.025 | * |
| Lithuania | 0.19 | -0.95 | 1.28 | 0.729 |  |
| Norway | 0.18 | -0.92 | 1.38 | 0.640 |  |
| United Kingdom | 1.16 | -3.78 | 4.23 | 0.826 |  |
| ***Western Europe*** |  |  |  |  |  |
| Austria | 0.20 | -1.57 | 1.95 | 0.828 |  |
| France | -0.06 | -0.62 | 0.47 | 0.796 |  |
| Germany | -1.29 | -5.47 | 0.79 | 0.157 |  |
| Netherlands | -0.91 | -1.08 | -0.76 | <0.001 | * |
| Switzerland | -0.34 | -1.58 | 0.86 | 0.500 |  |
| ***Southern Europe*** |  |  |  |  |  |
| Croatia | -1.72 | -2.10 | -1.26 | <0.001 | * |
| Cyprus | 0.30 | -0.73 | 1.90 | 0.601 |  |
| Italy | -0.02 | -8.80 | 5.79 | 0.658 |  |
| Malta | -2.82 | -4.67 | -1.06 | 0.002 | * |
| Slovenia | -1.54 | -2.57 | -0.56 | 0.002 | * |
| Spain | 1.89 | -4.13 | 5.03 | 0.601 |  |
| ***Eastern Europe*** |  |  |  |  |  |
| Belarus | 1.67 | 1.06 | 2.26 | <0.001 | * |
| Czech | 0.45 | 0.07 | 0.81 | 0.024 | * |
| Poland | 0.17 | -1.59 | 1.87 | 0.883 |  |
| ***Africa*** |  |  |  |  |  |
| Uganda | -1.97 | -9.50 | 6.09 | 0.620 |  |

AAPC, annual percentage change; CI, confidence interval; * p values less than 0·05.

**Figure S1.** Plots of Joinpoint regression for trend analysis

1. a) Both

| **Asia** | |
| --- | --- |
| 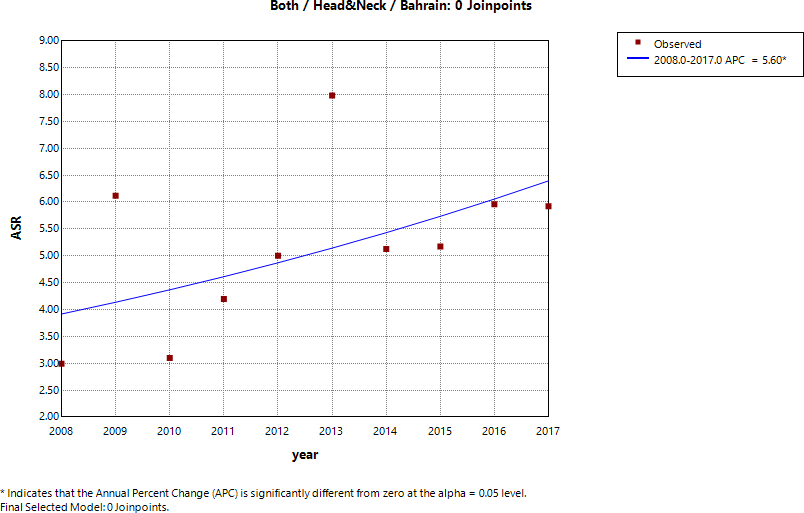 | 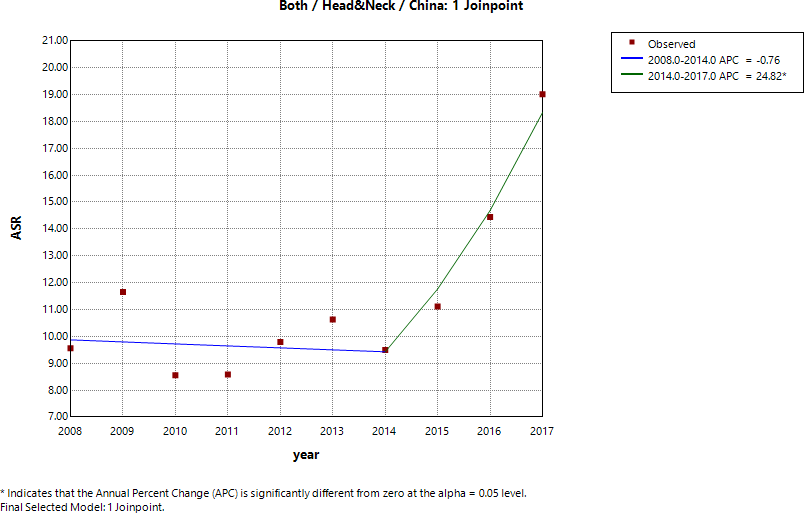 |
| 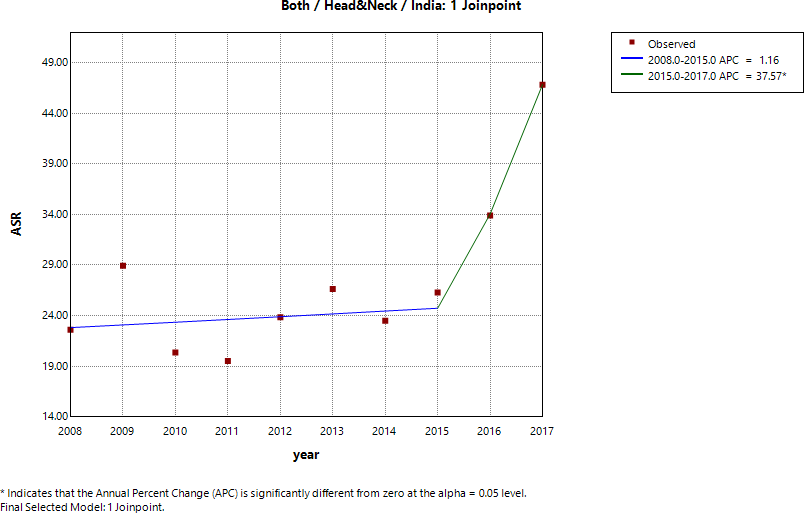 | 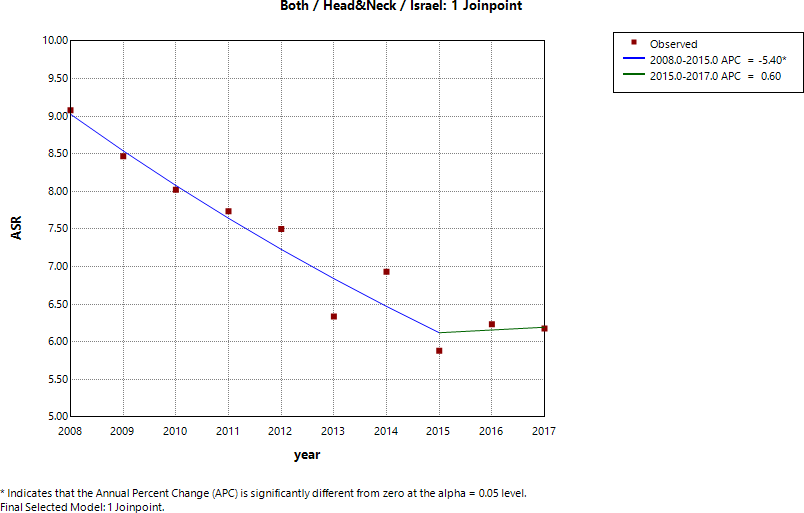 |
| 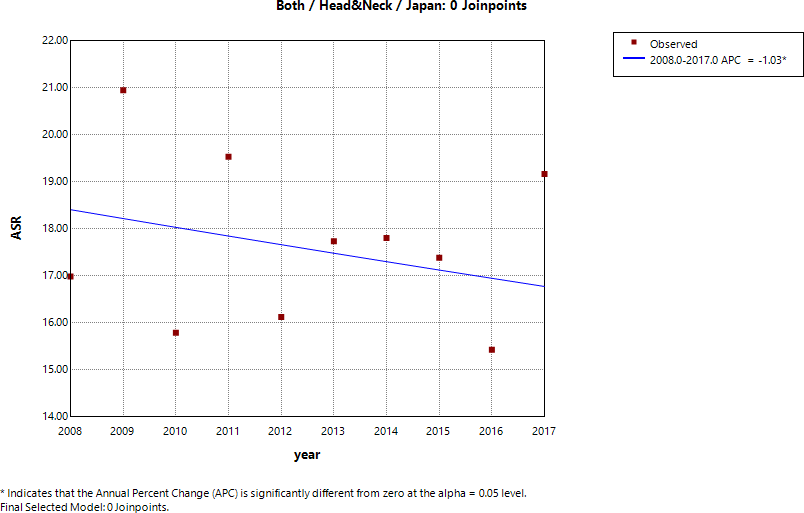 | 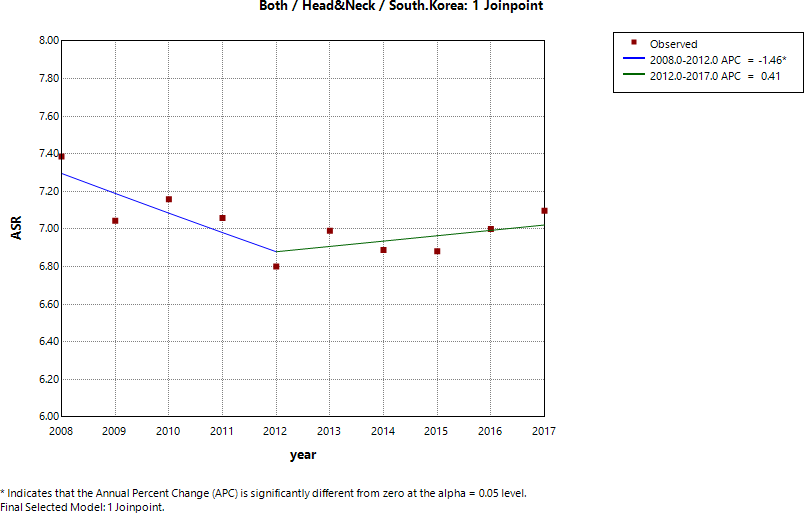 |

| 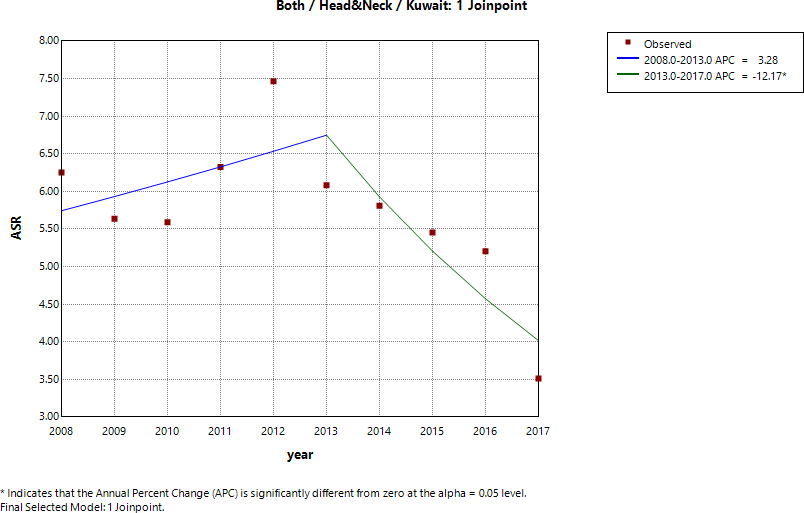 | 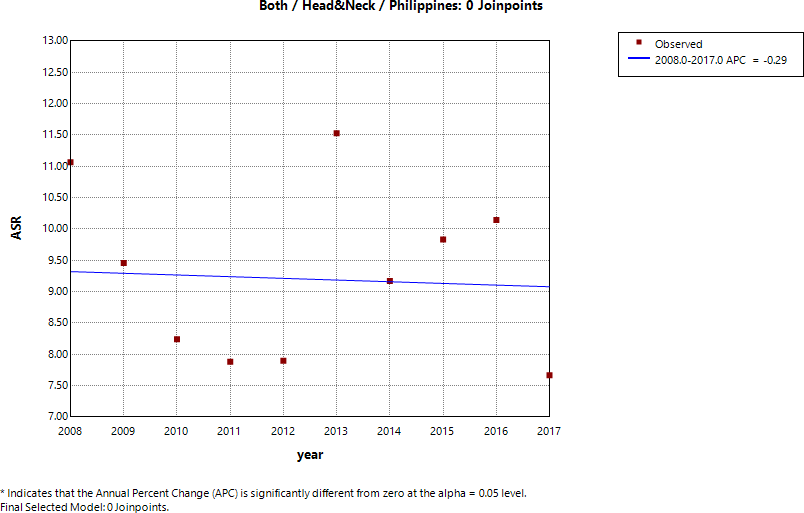 |
| --- | --- |
| 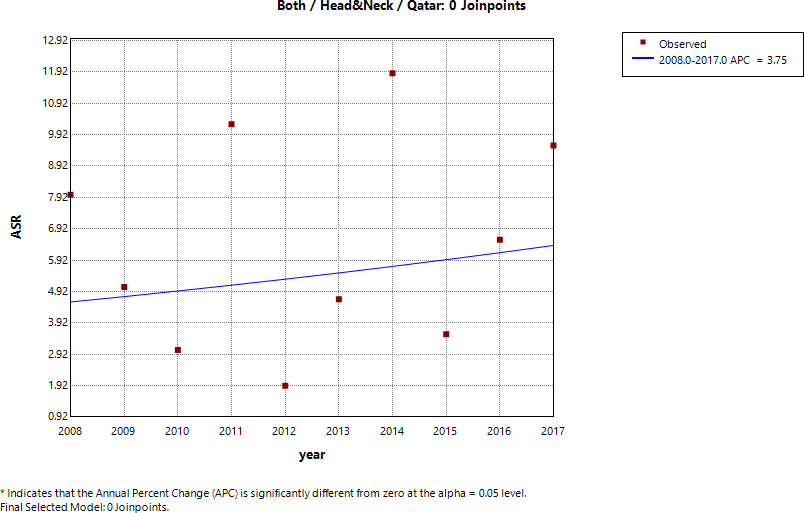 | 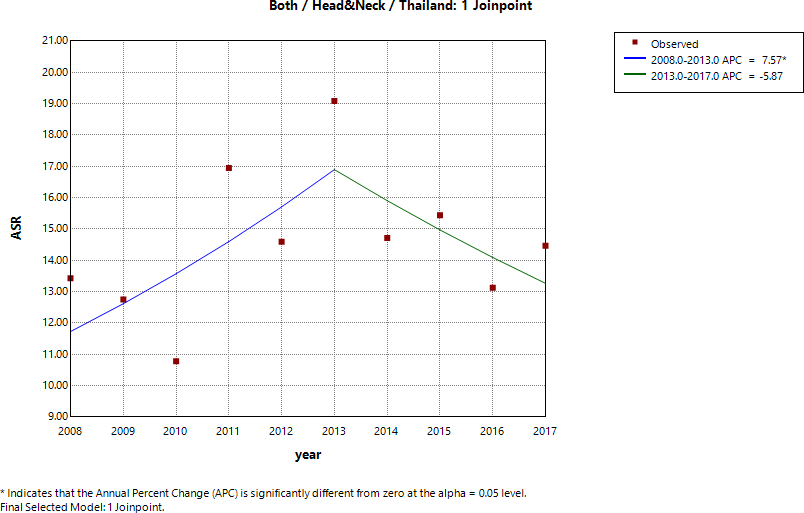 |
| 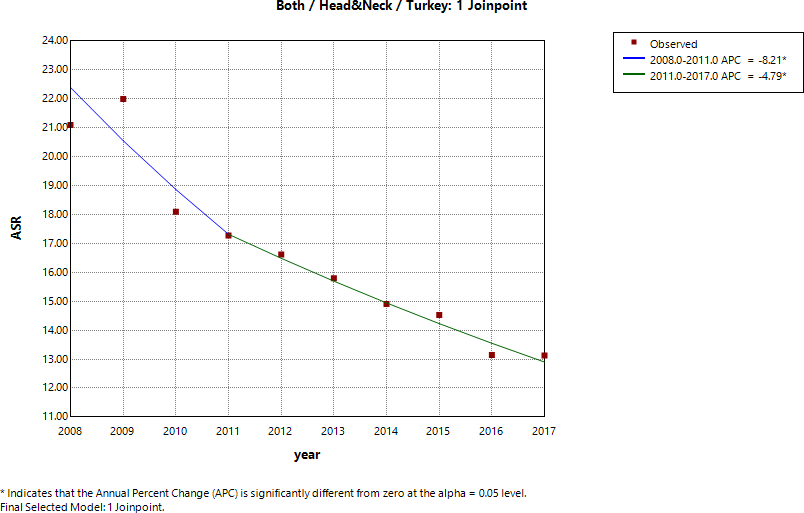 |  |

| **Oceania** | |
| --- | --- |
| 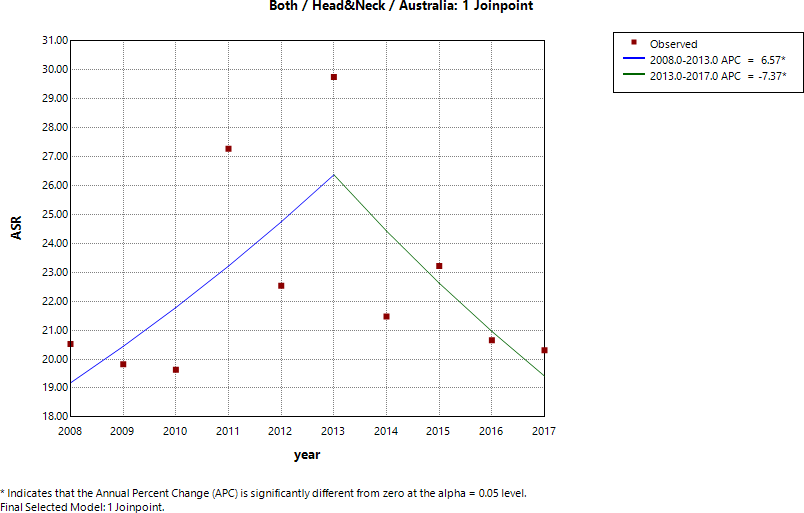 | 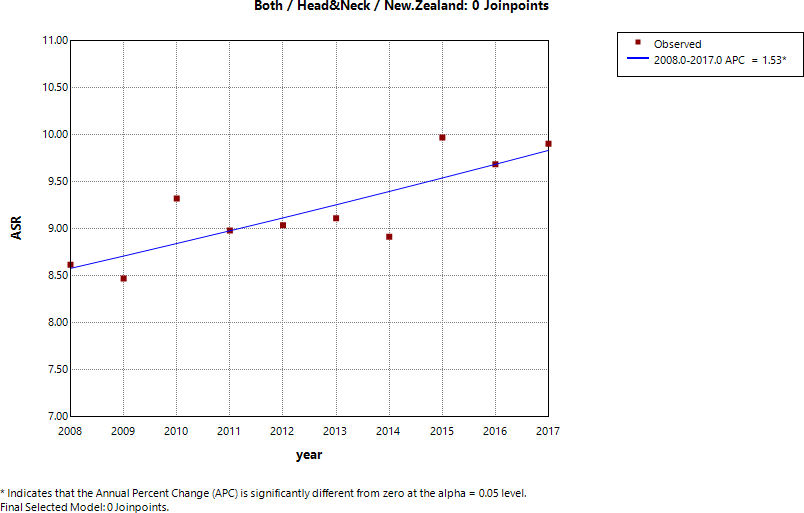 |
| **Northern America** | |
| 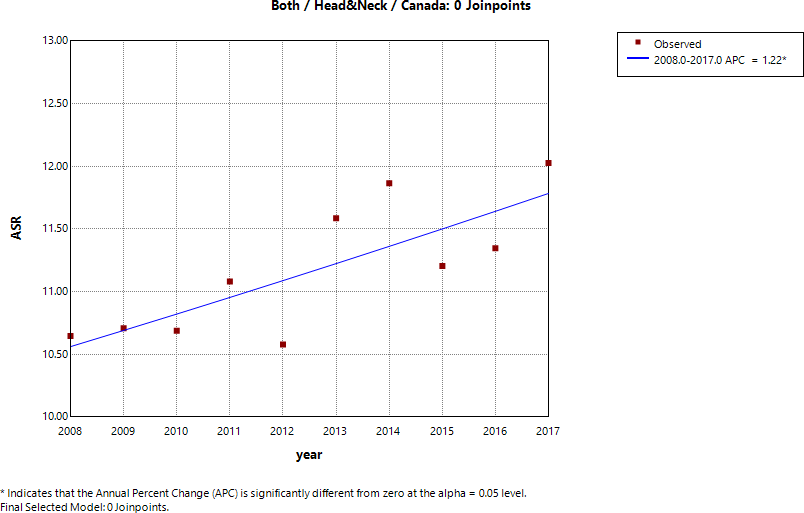 | 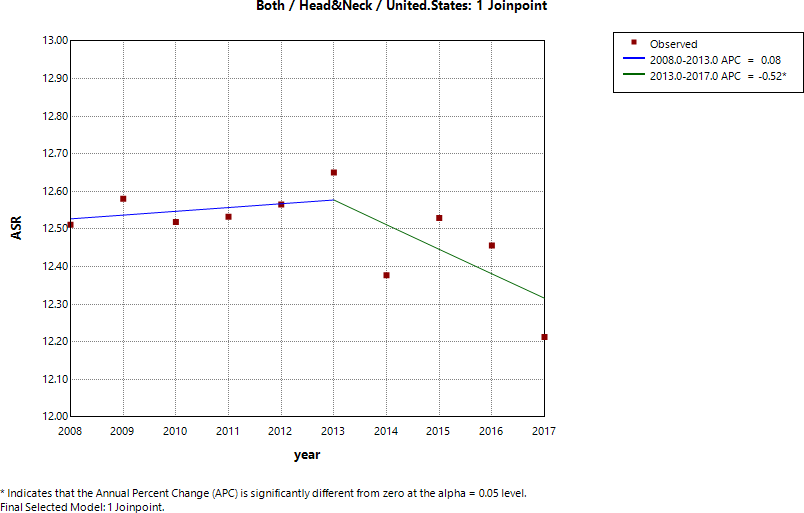 |
| **Southern America** | |
| 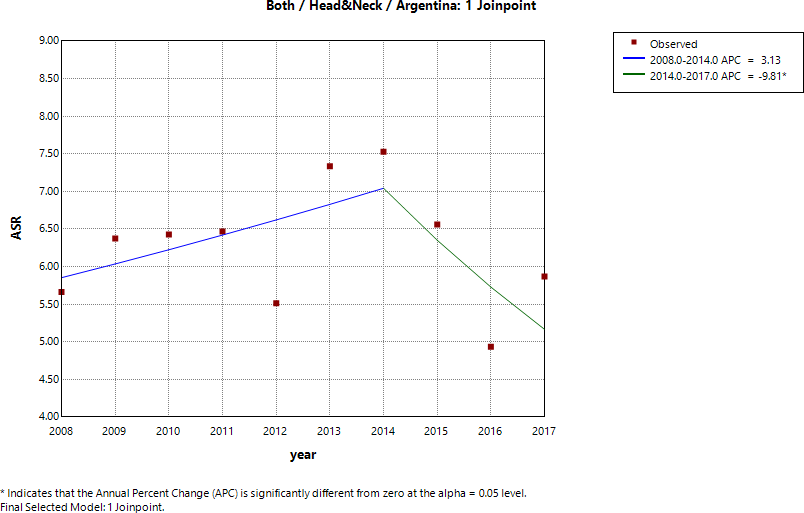 | 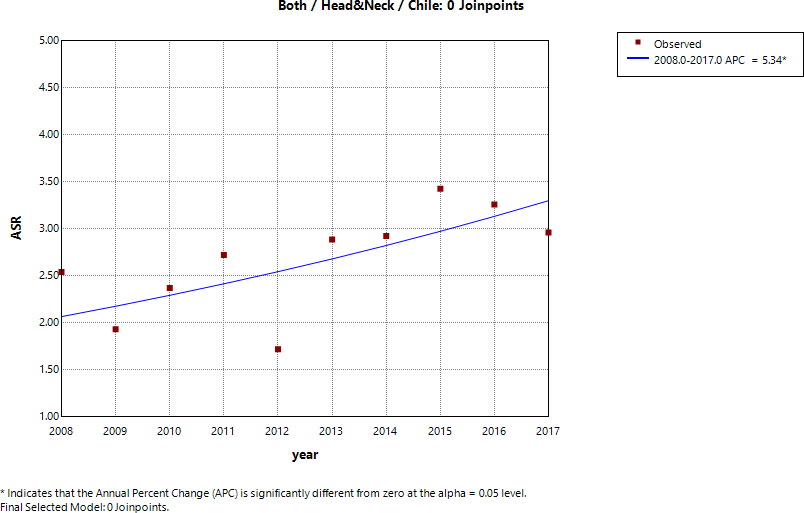 |

| 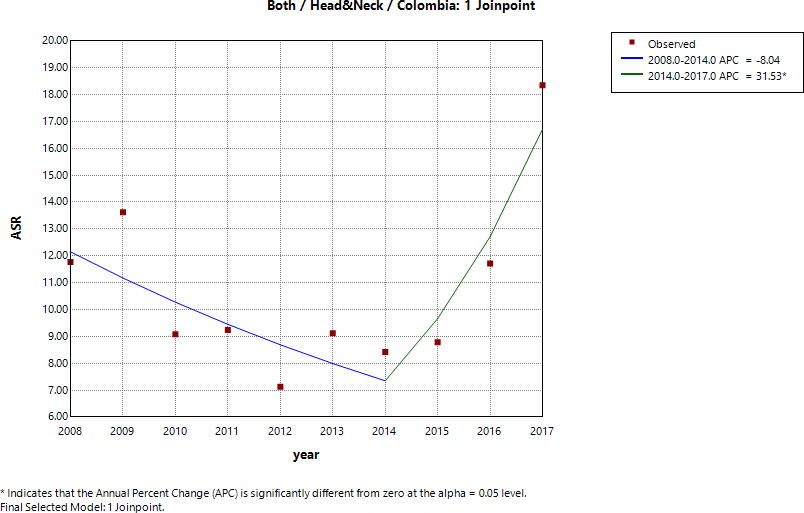 | 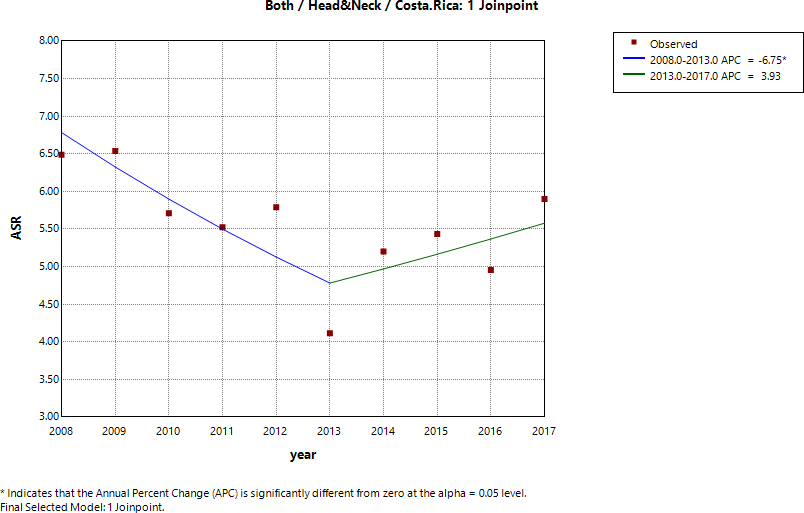 |
| --- | --- |
| 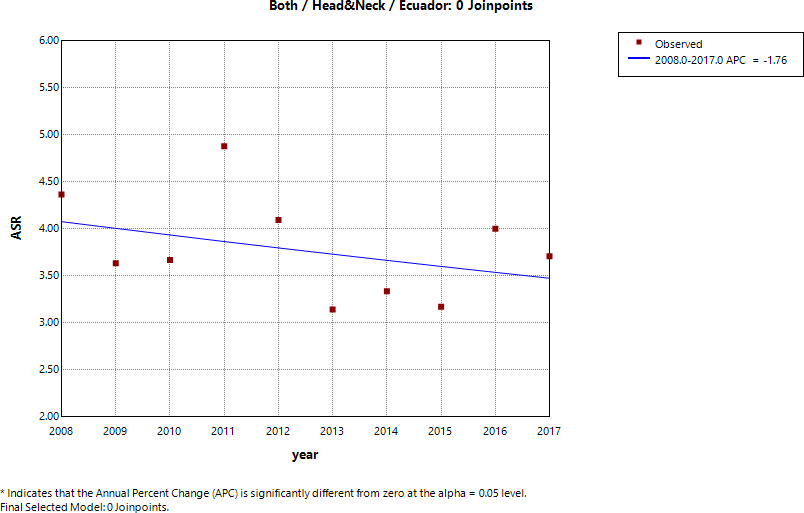 | 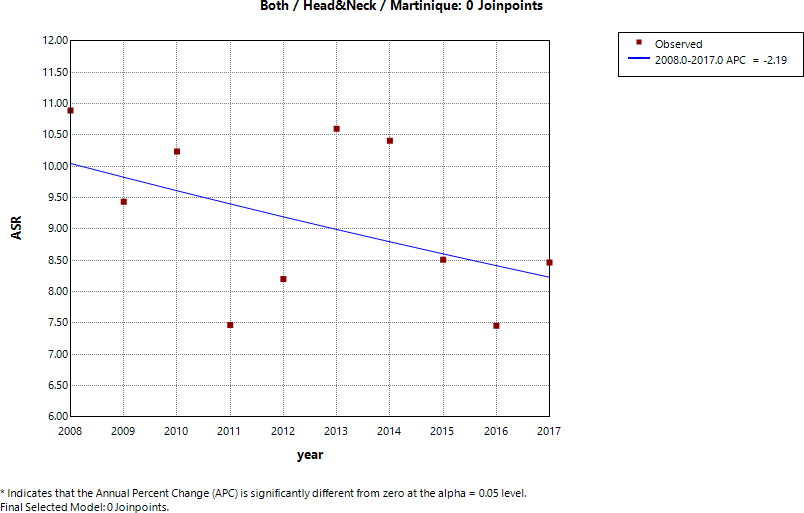 |
| 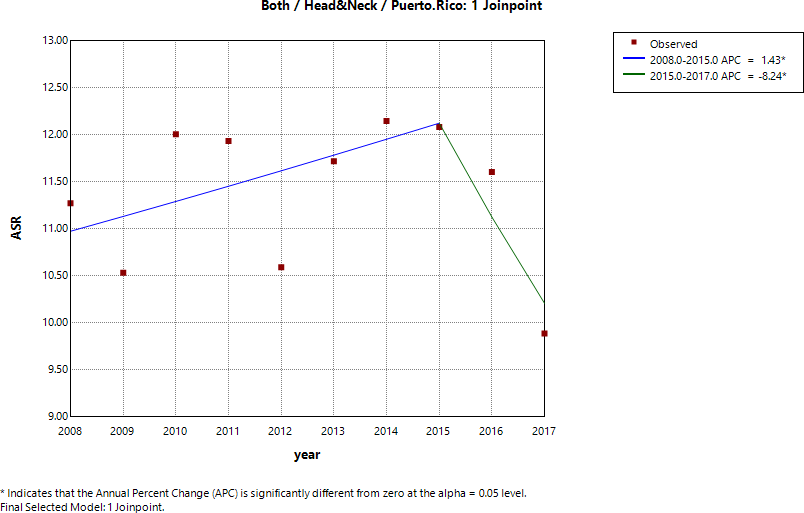 |  |
| **Northern Europe** | |
| 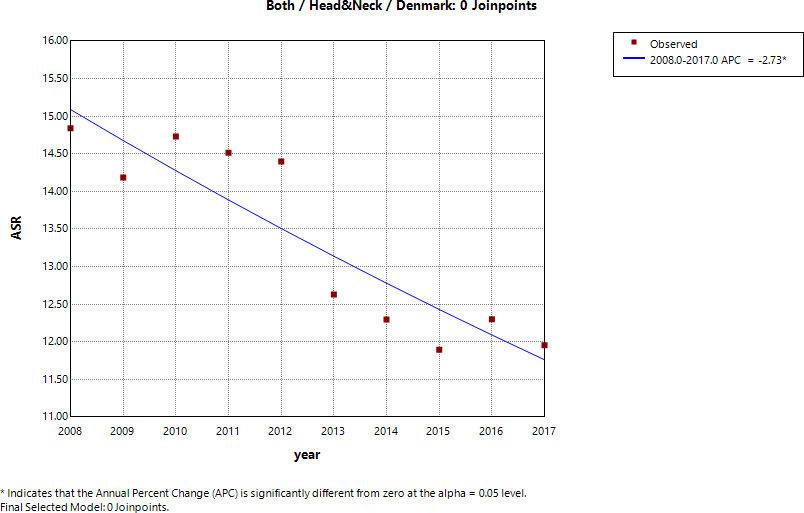 | 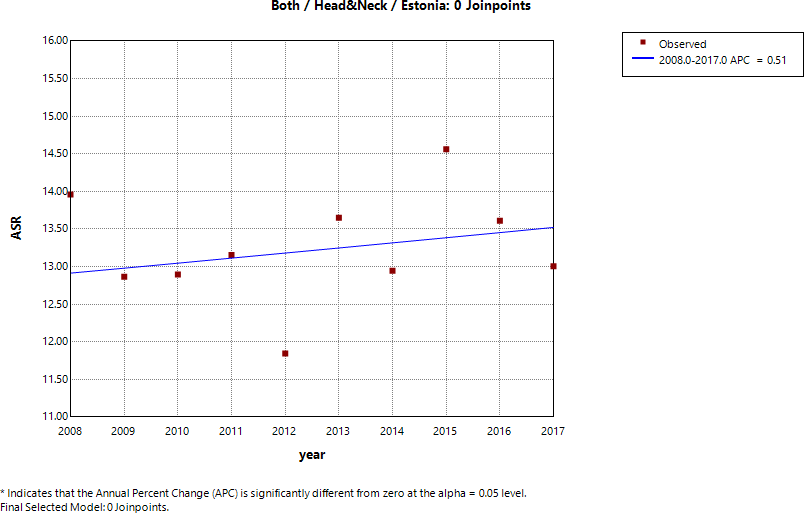 |

| 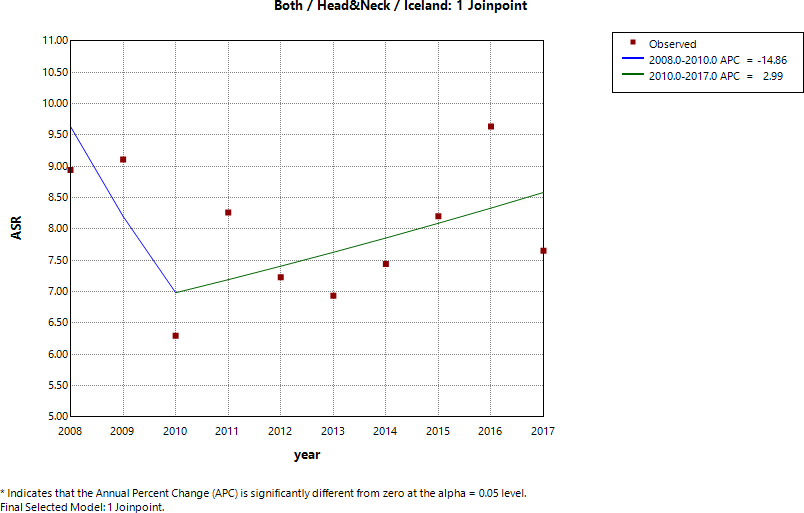 | 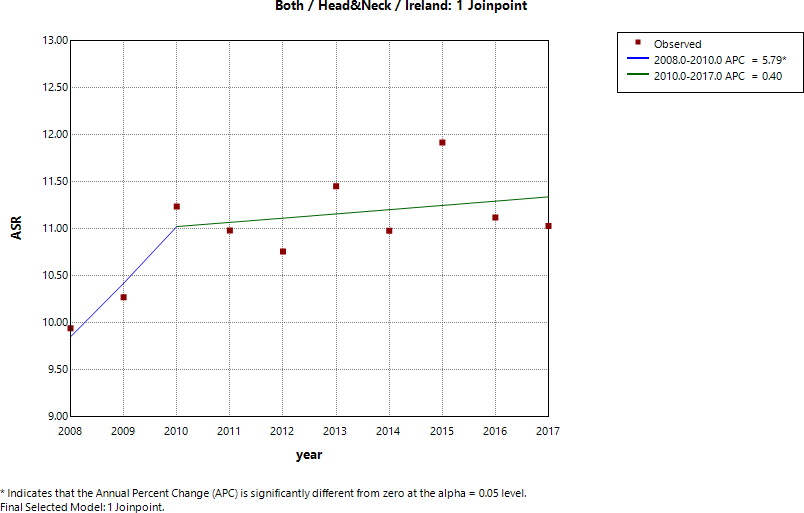 |
| --- | --- |
| 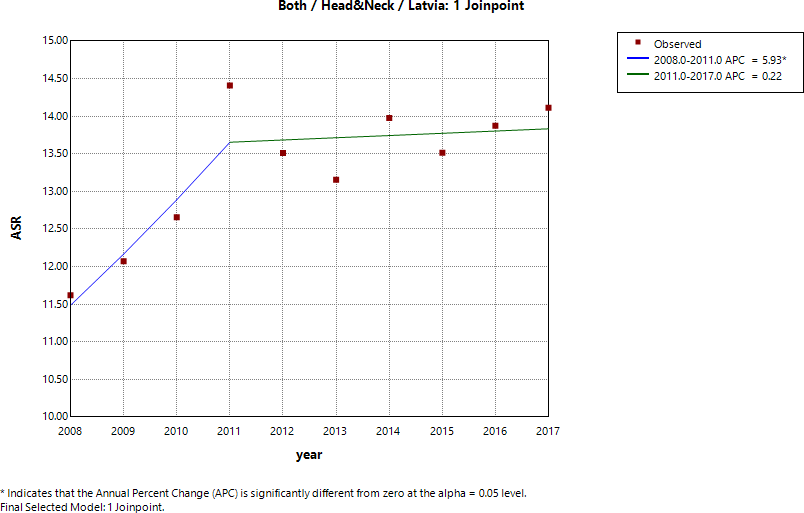 | 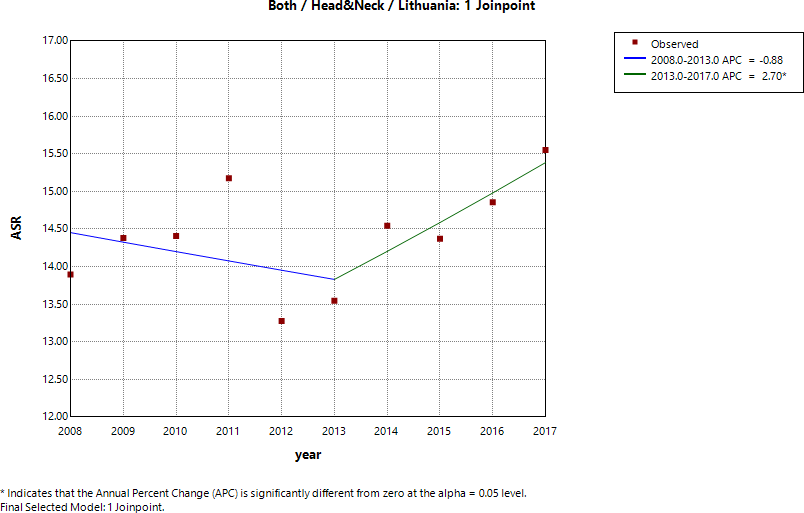 |
| 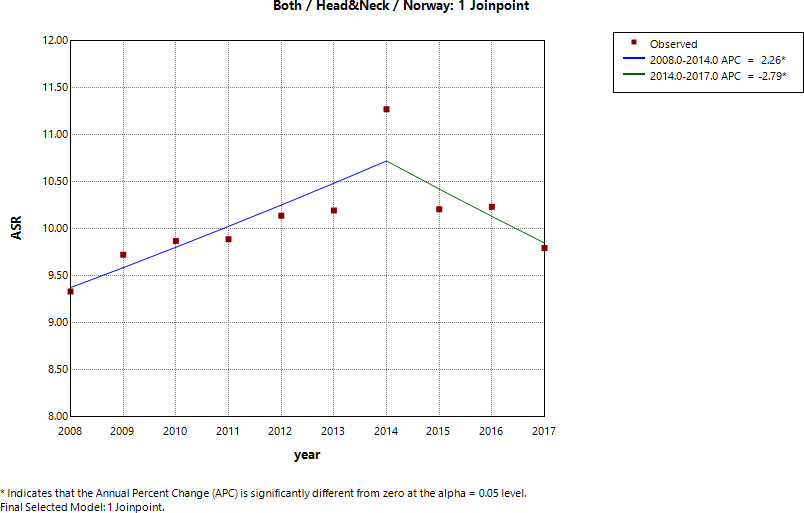 | 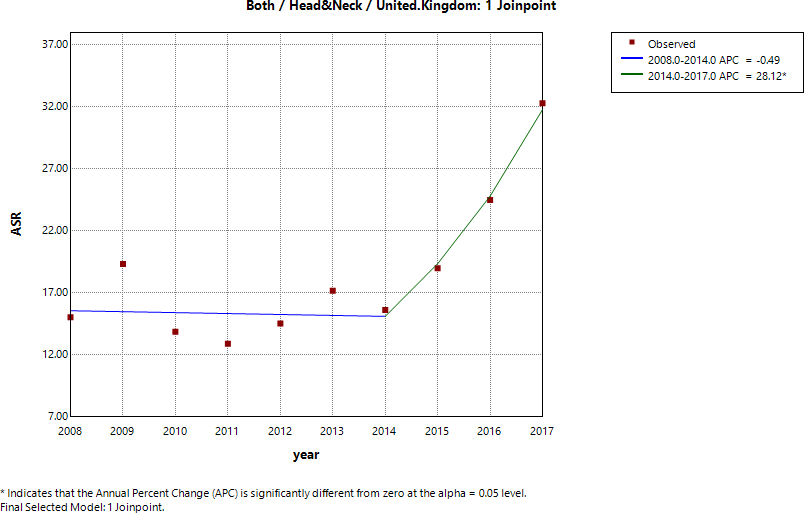 |
| **Western Europe** | |
| 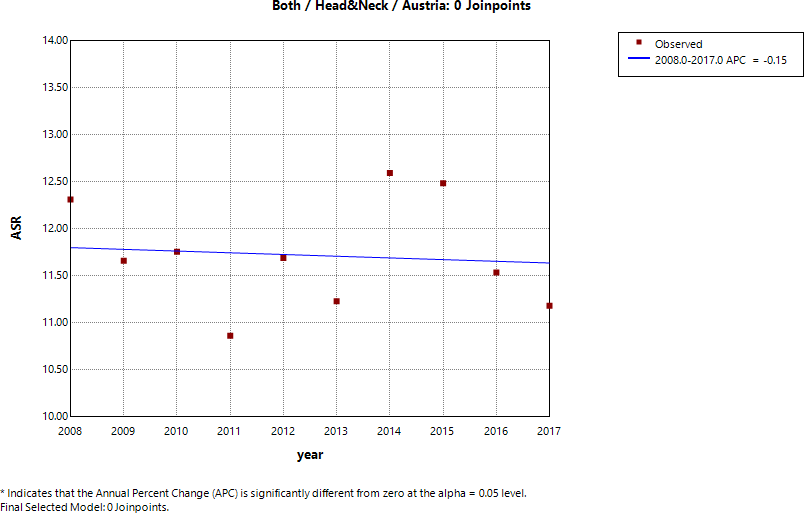 | 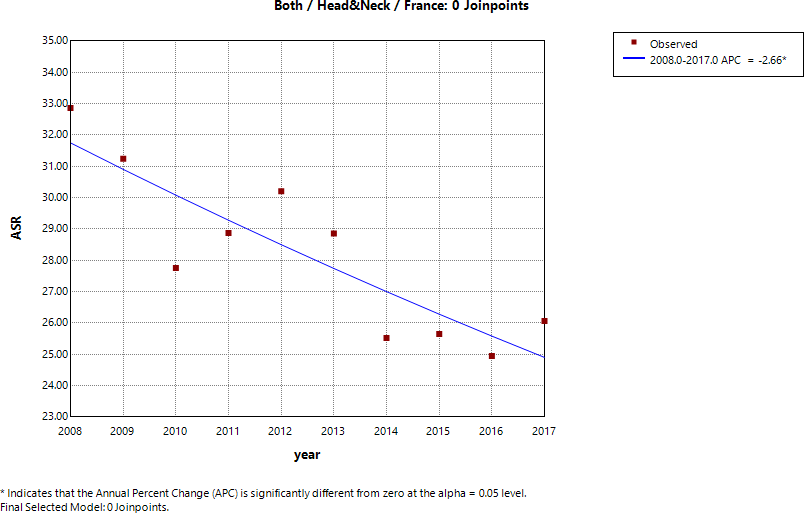 |

| 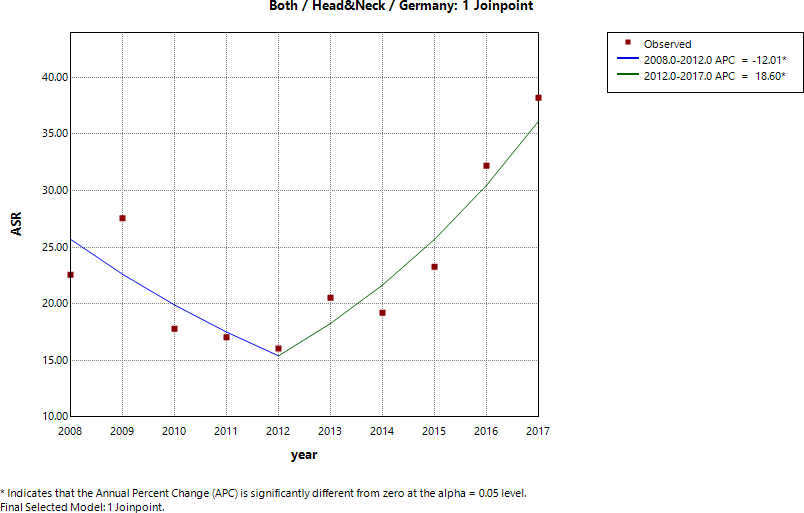 | 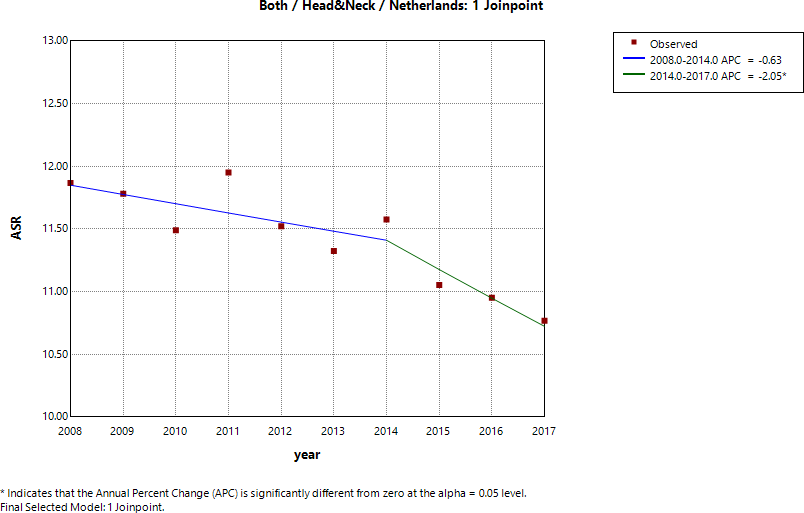 |
| --- | --- |
| 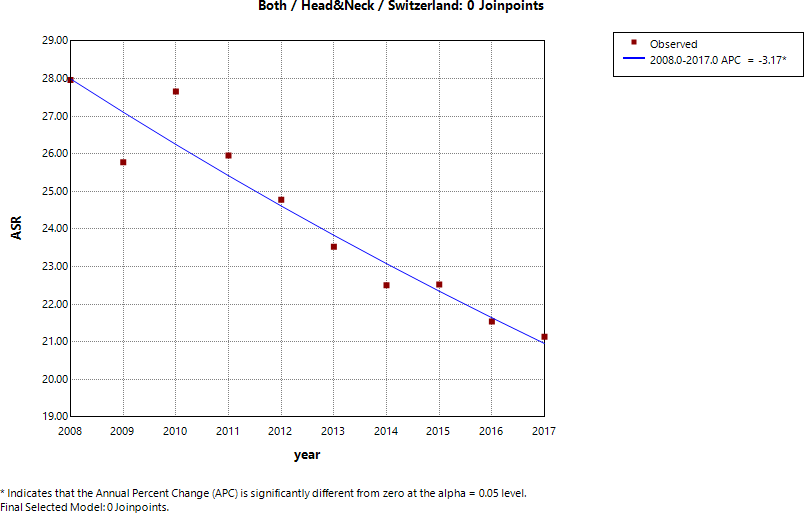 |  |
| **Southern Europe** | |
| 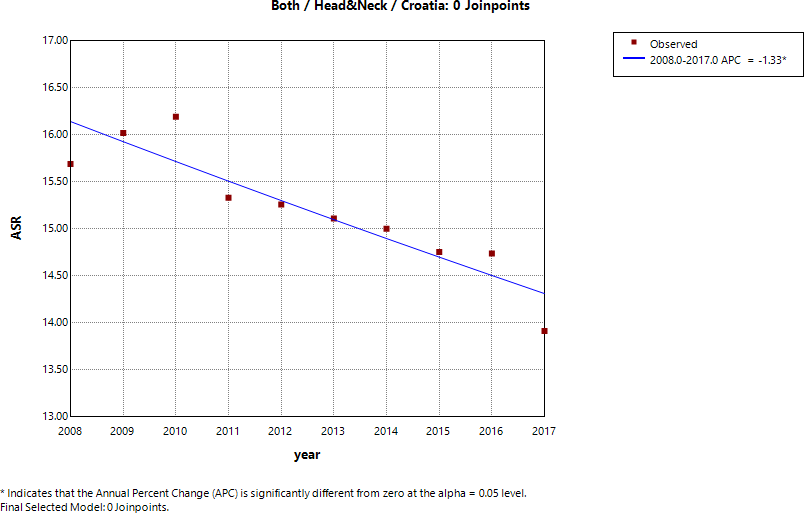 | 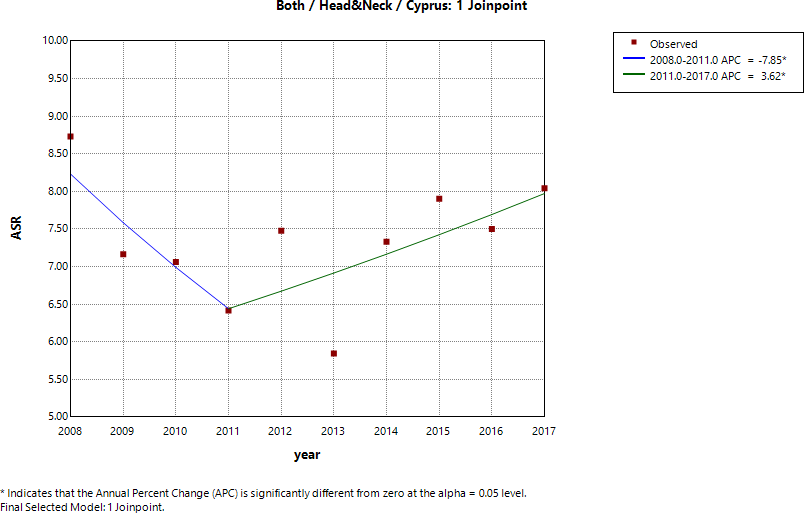 |

| 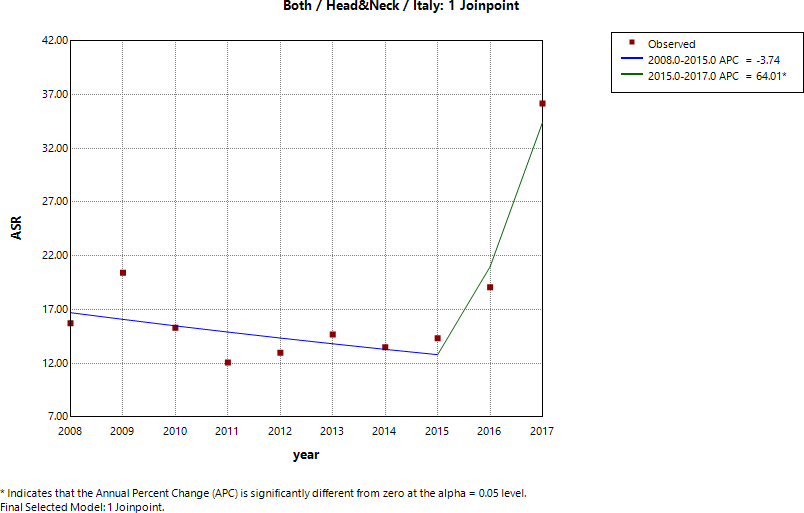 | 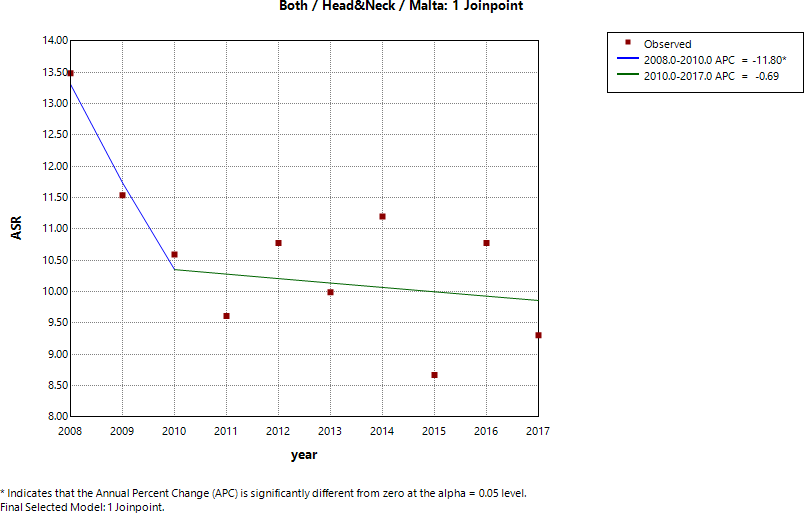 |
| --- | --- |
| 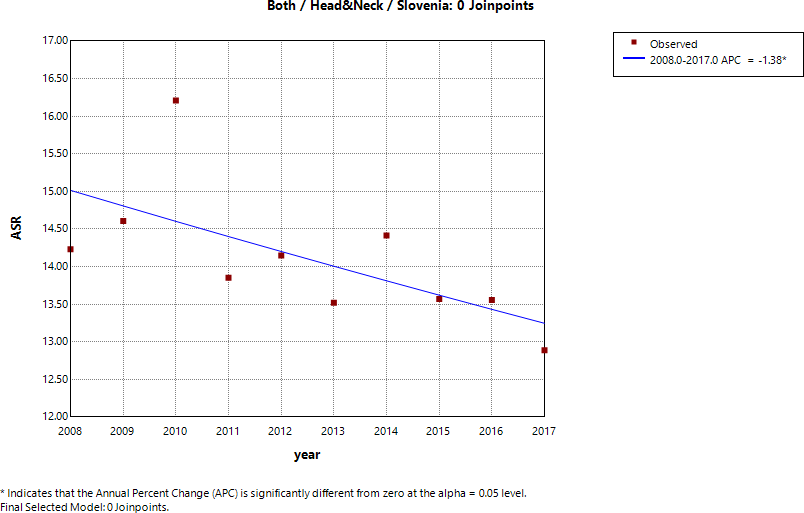 | 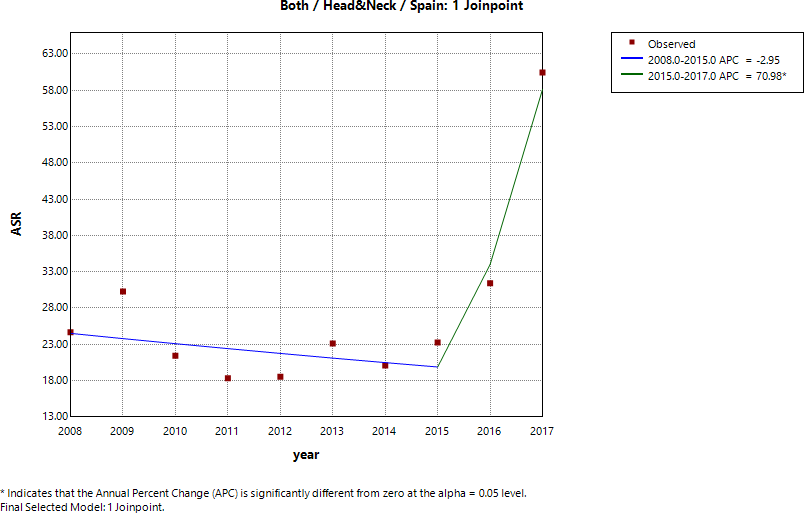 |
| **Eastern Europe** | |
| 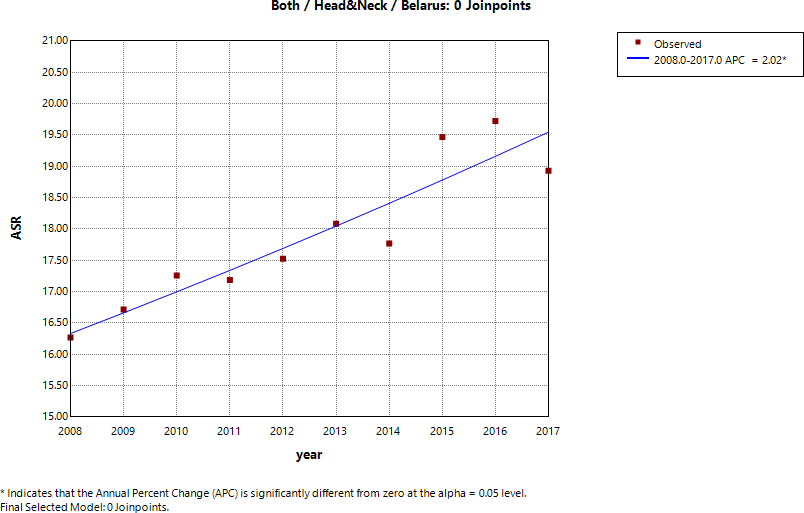 | 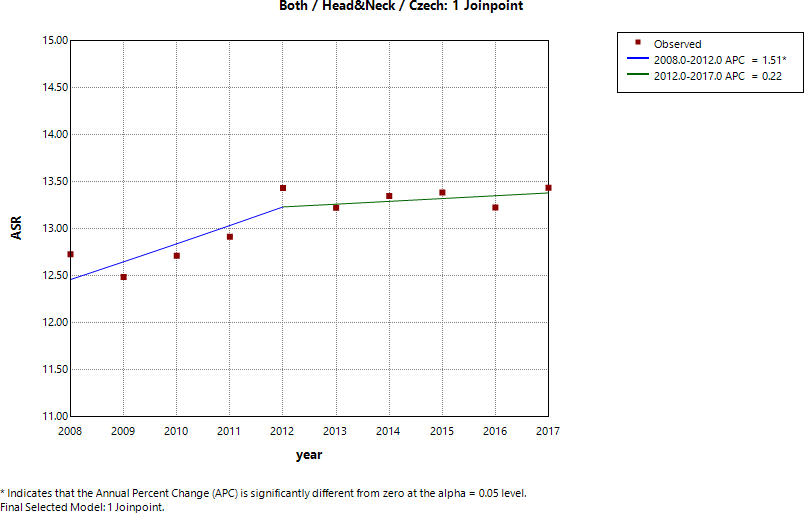 |
| 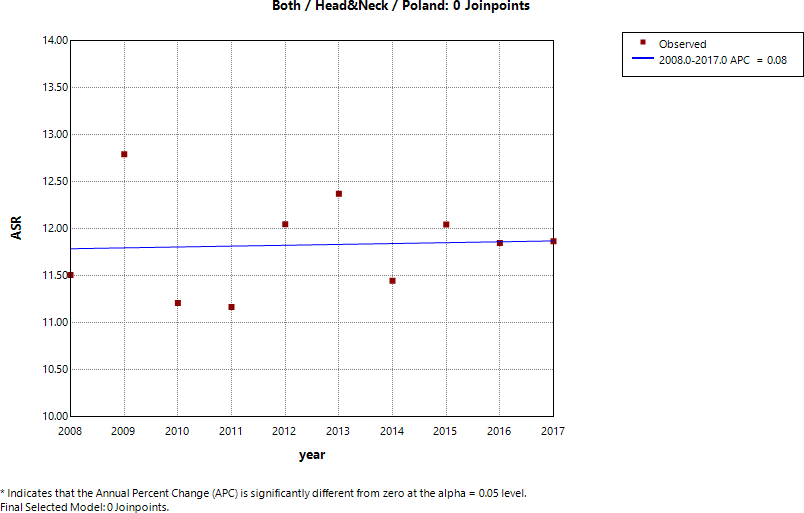 |  |

| **Africa** |
| --- |
| 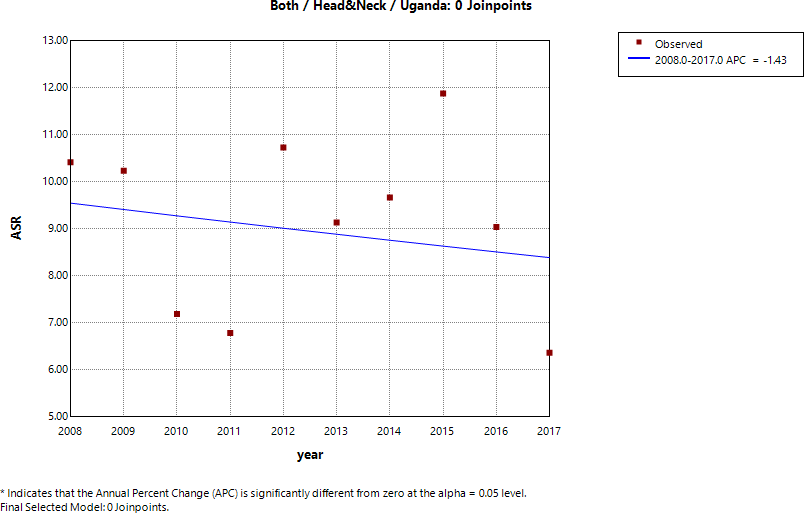 |

1b) Male

| **Asia** | |
| --- | --- |
| 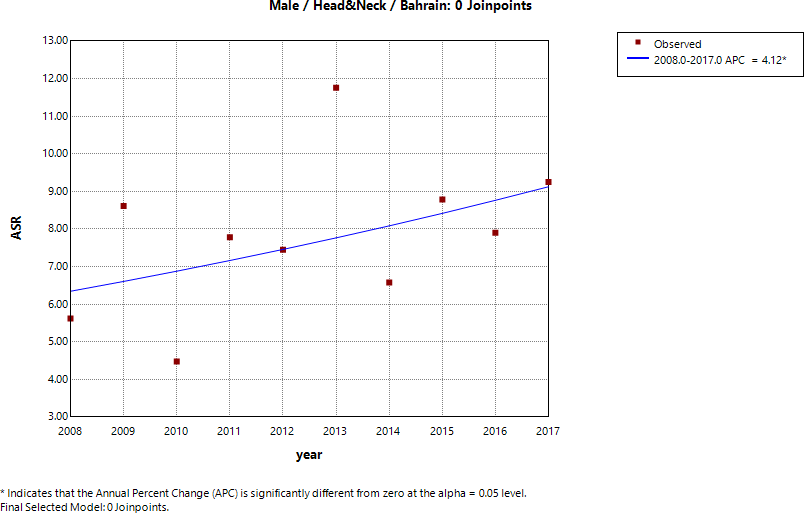 | 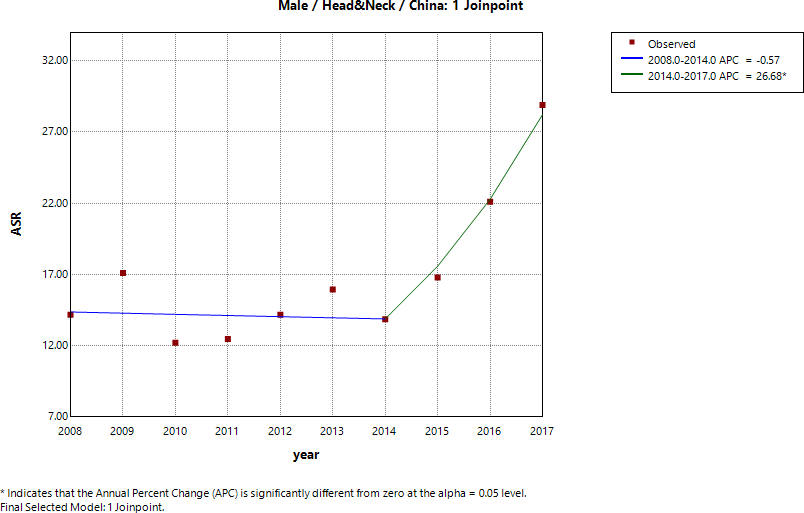 |
| 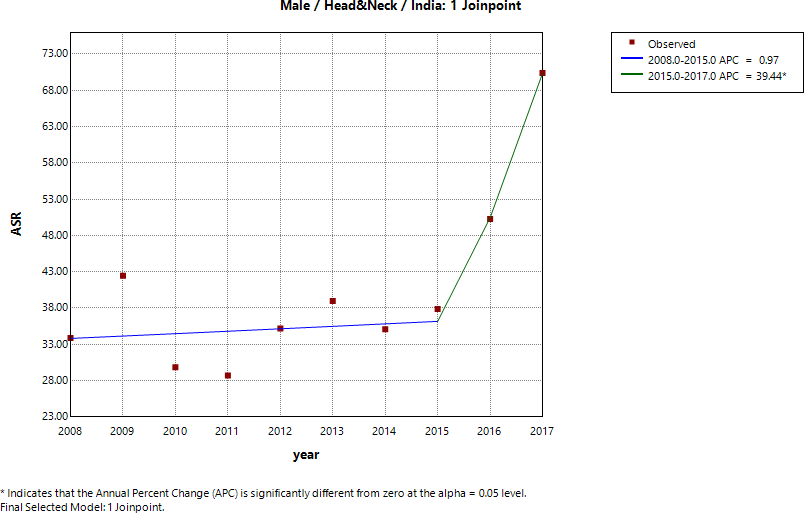 | 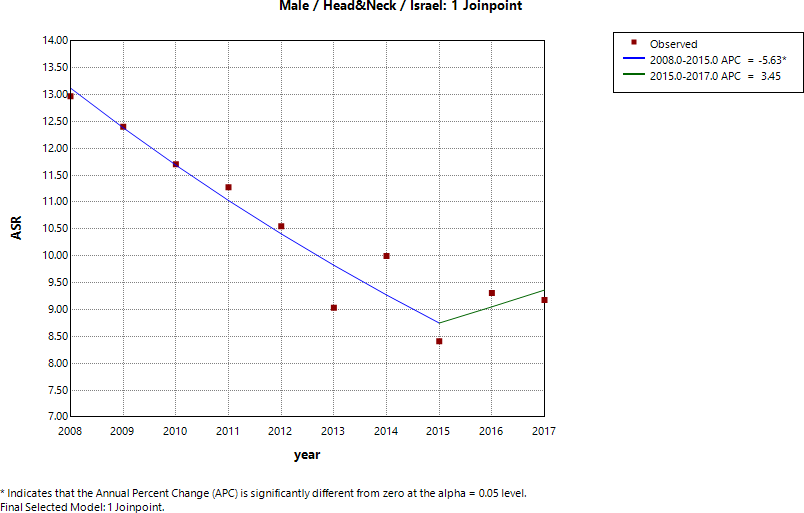 |
| 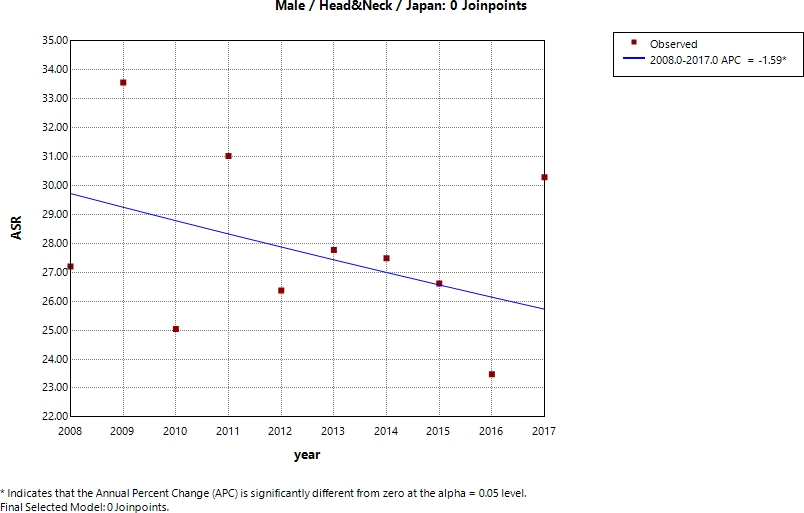 | 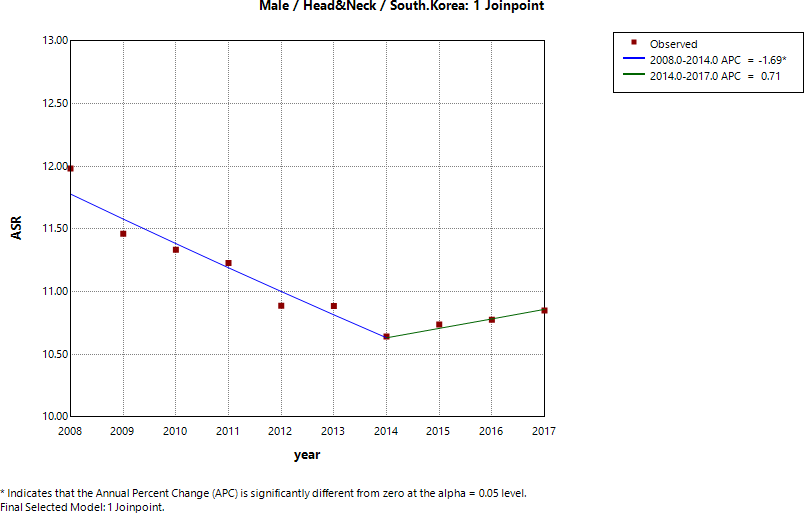 |

| 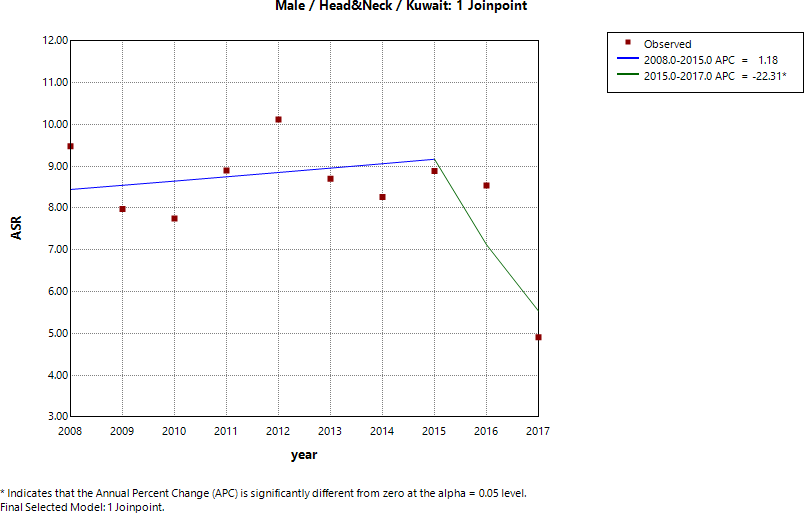 | 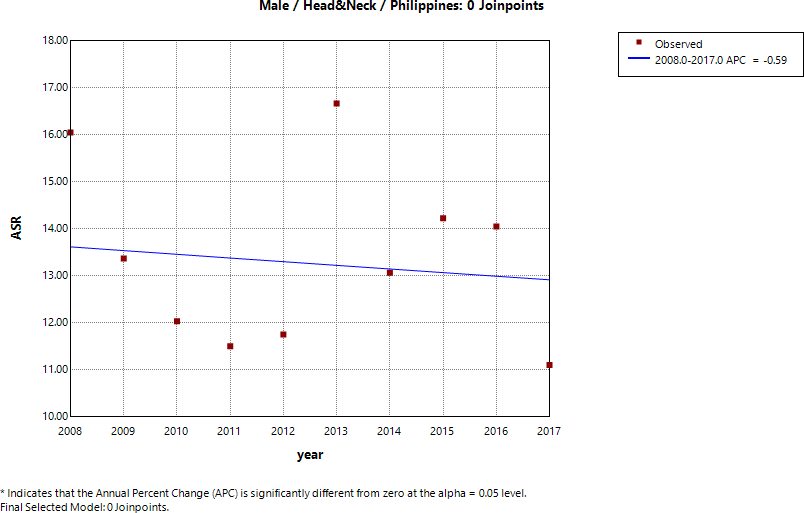 |
| --- | --- |
| 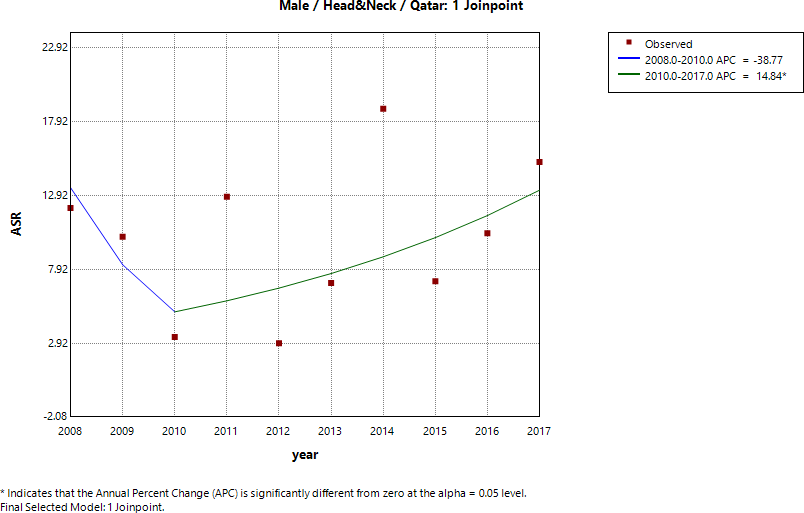 | 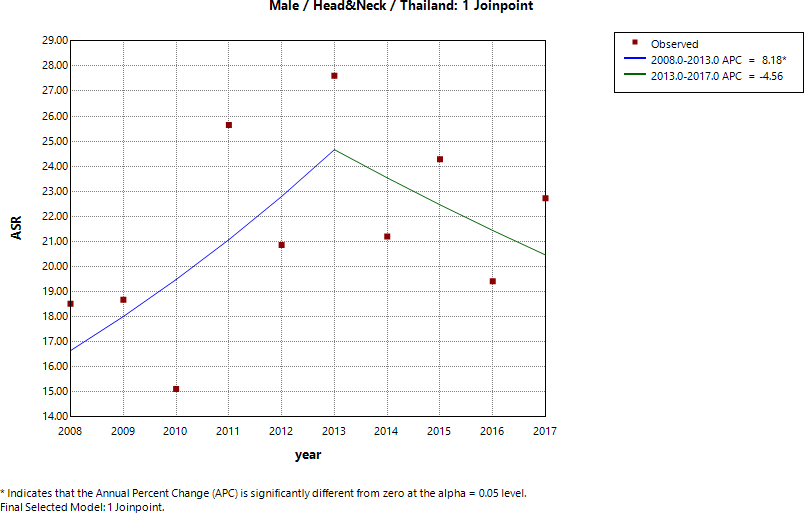 |
| 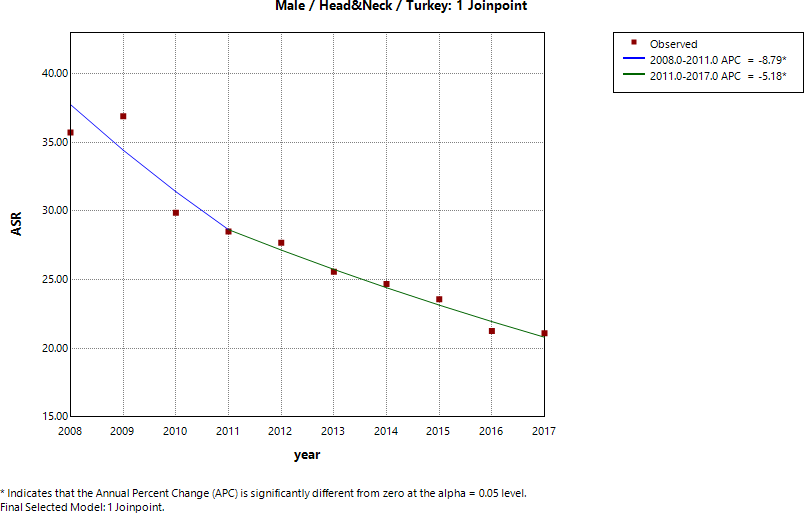 |  |
| **Oceania** | |
| 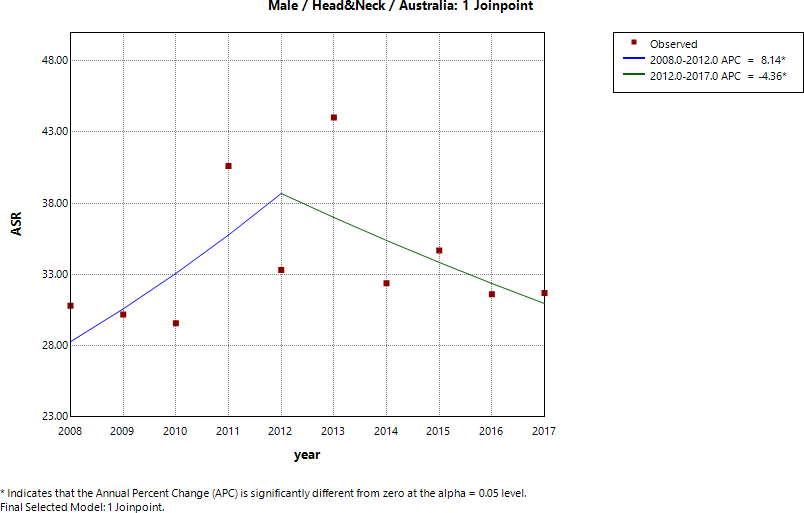 | 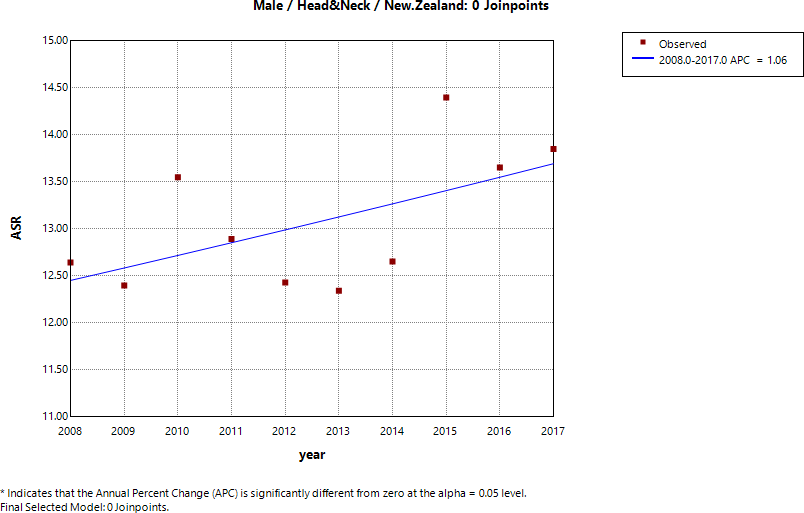 |

| **Northern America** | |
| --- | --- |
| 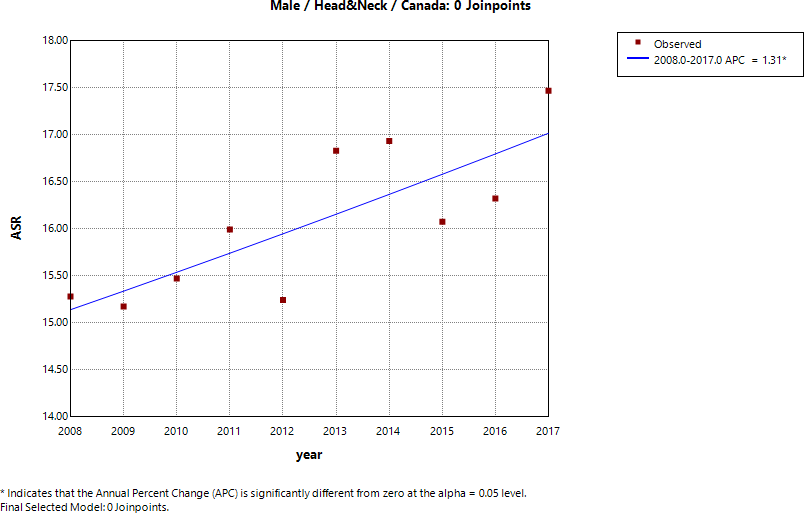 | 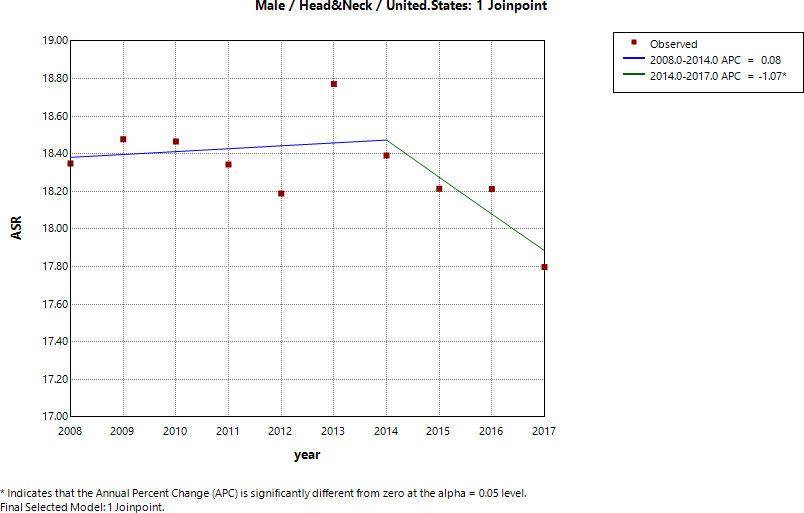 |
| **Southern America** | |
| 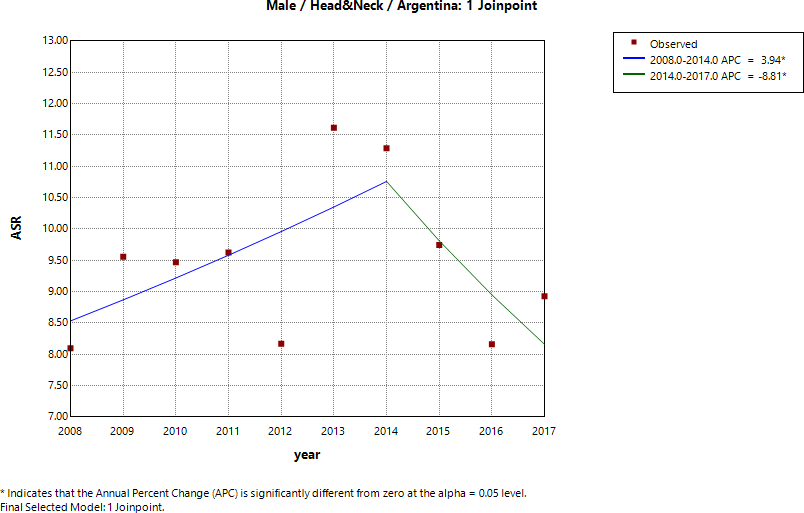 | 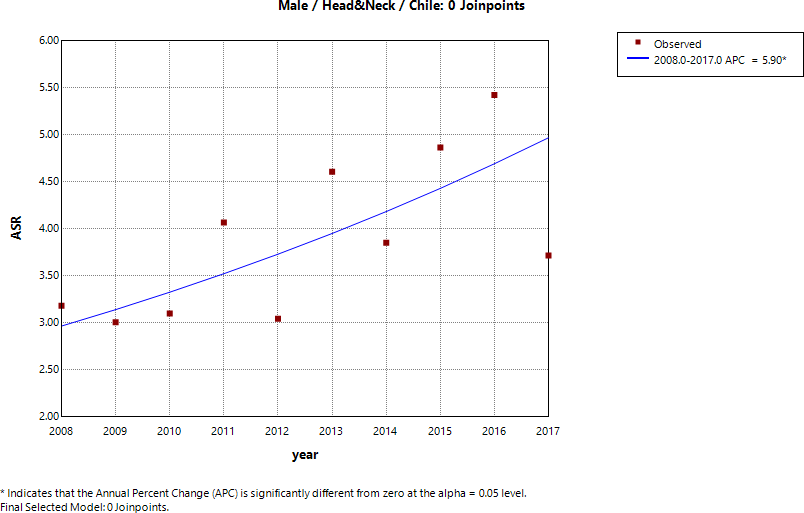 |
| 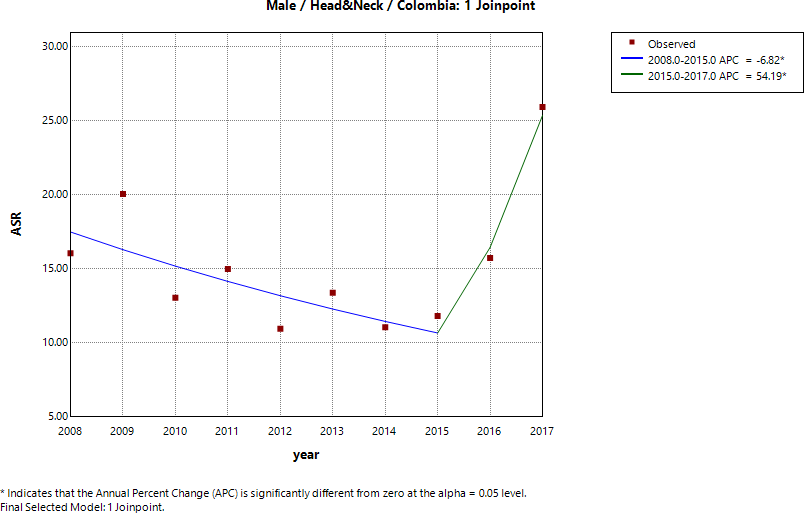 | 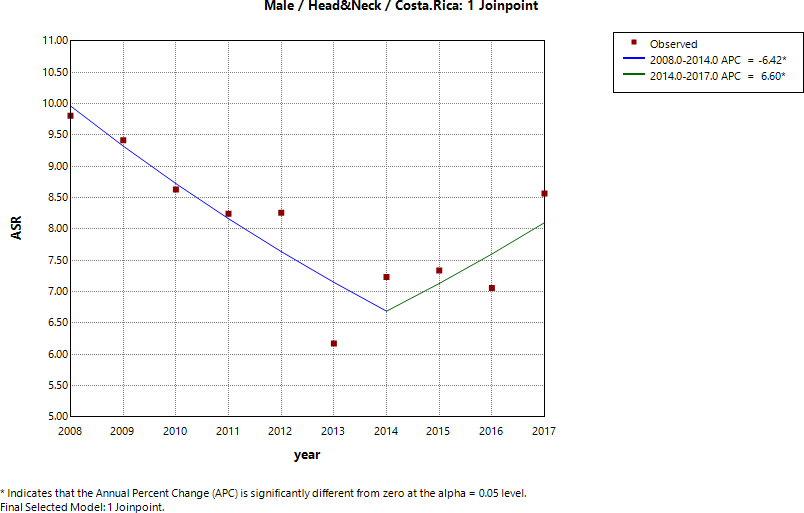 |

| 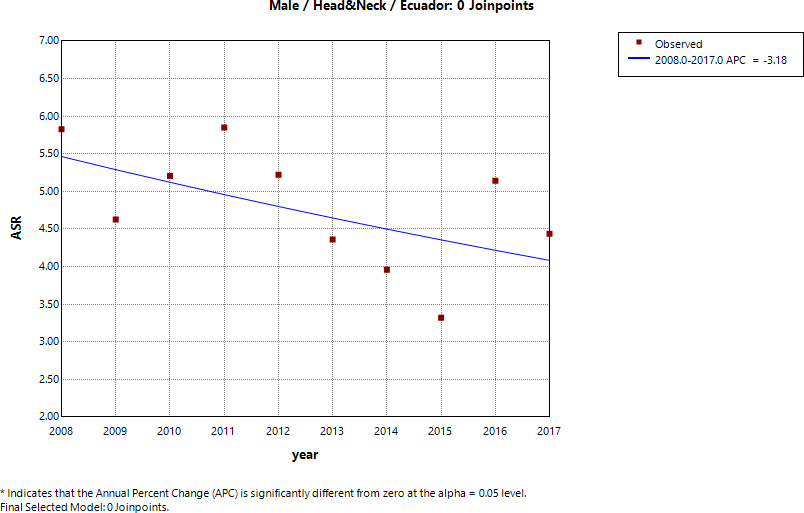 | 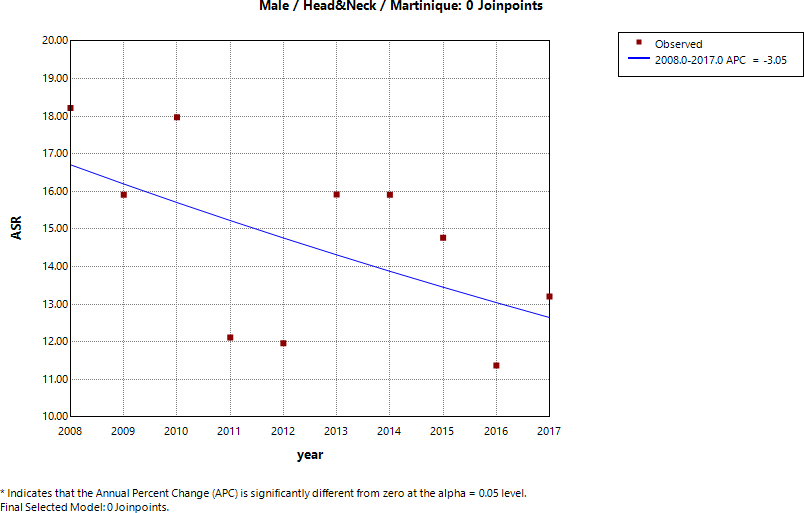 |
| --- | --- |
| 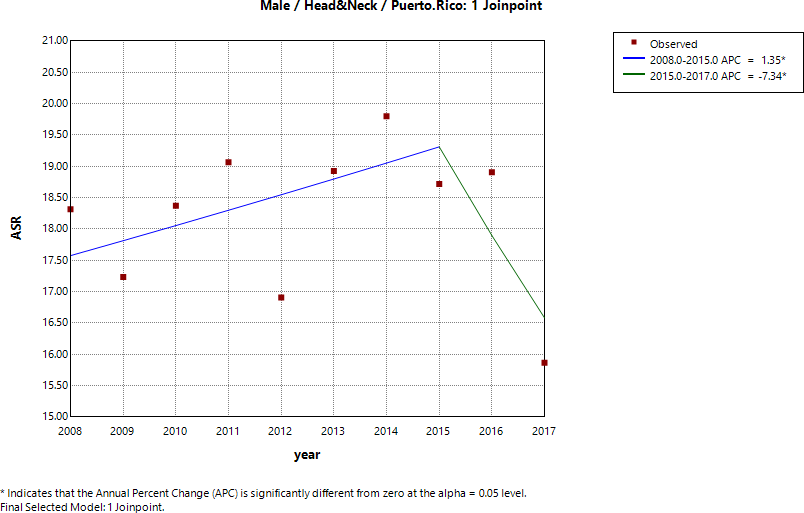 |  |
| **Northern Europe** | |
| 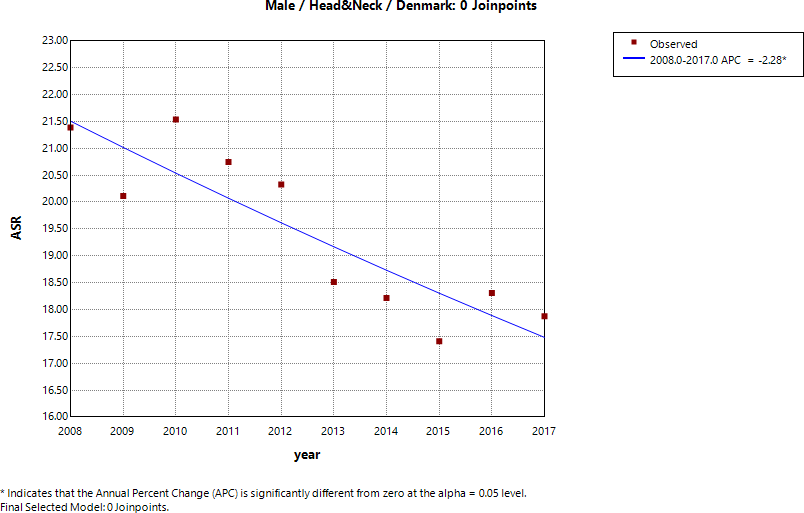 | 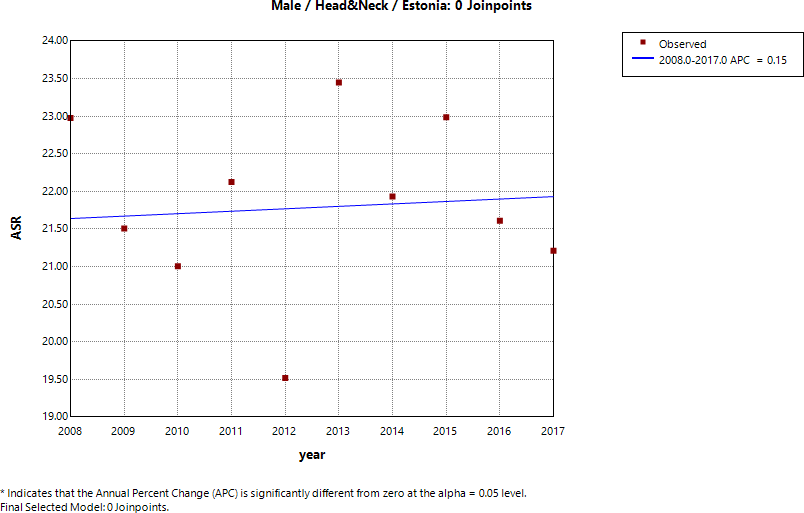 |

| 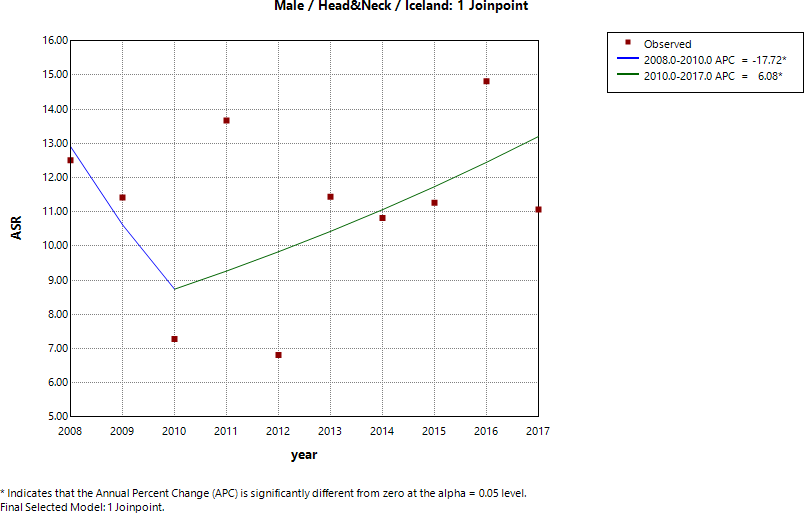 | 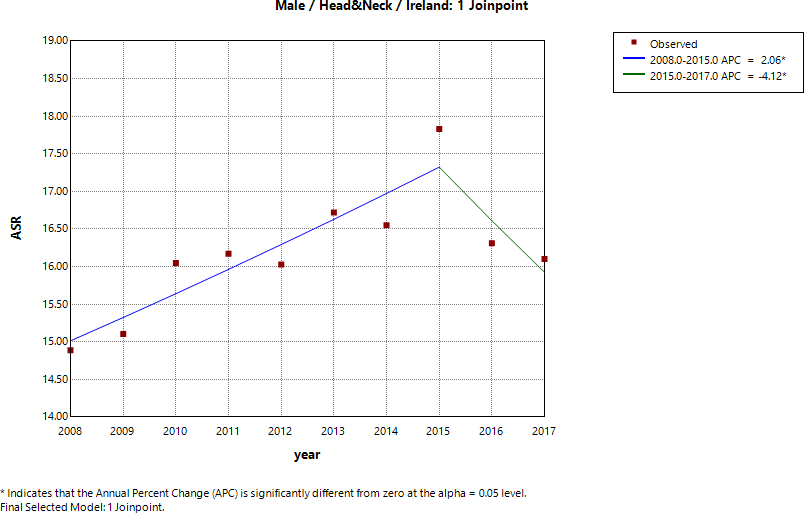 |
| --- | --- |
| 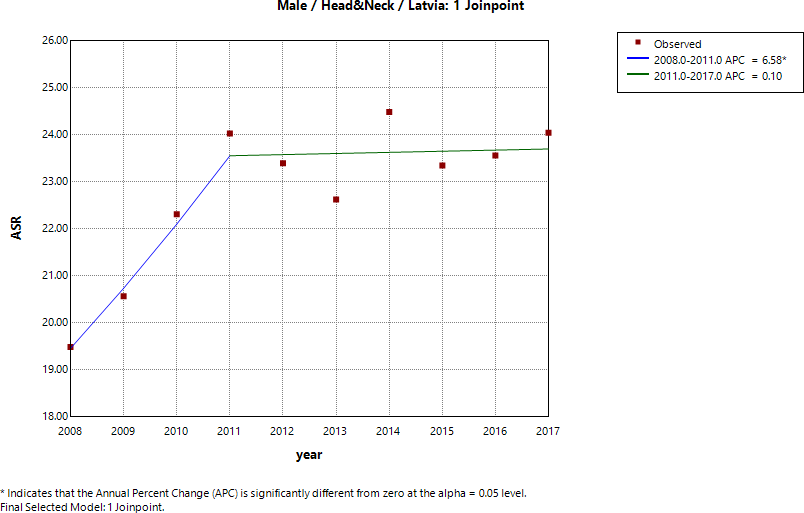 | 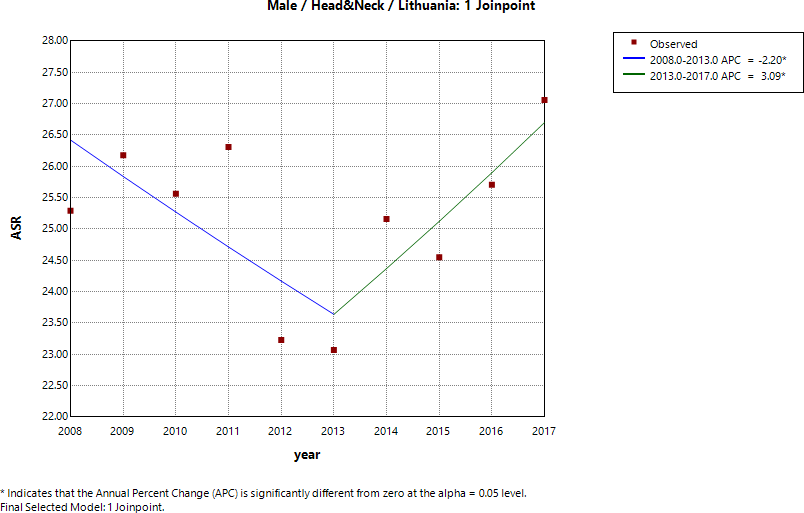 |
| 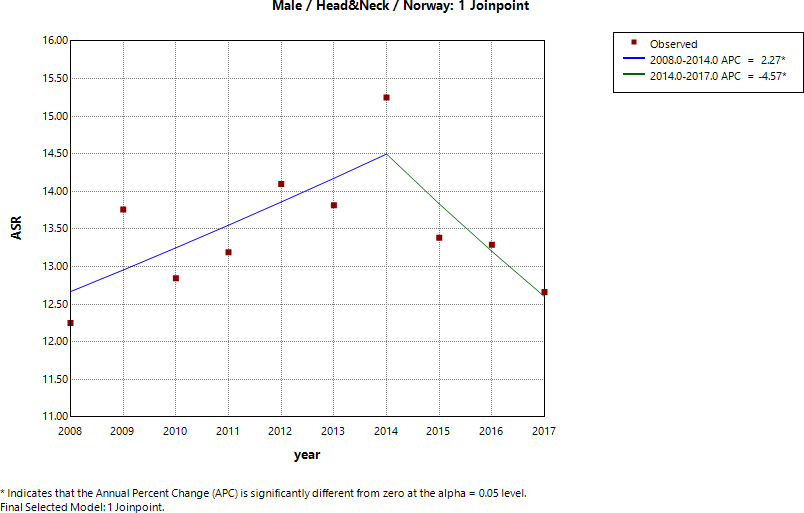 | 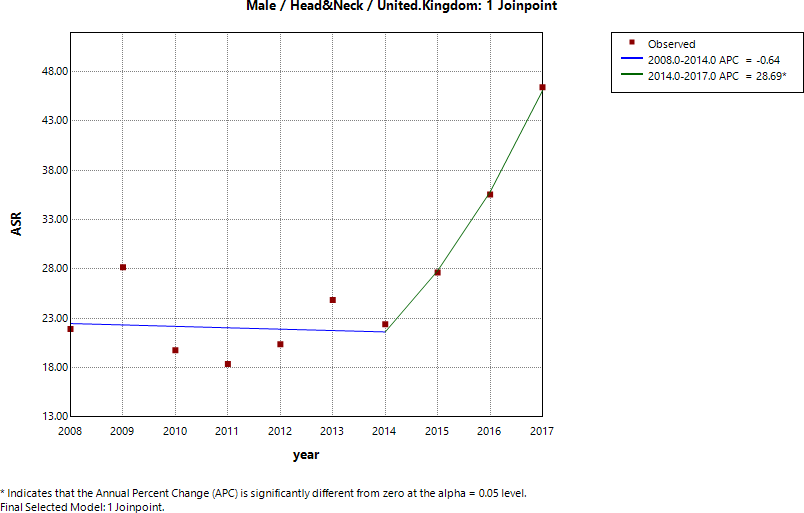 |

| **Western Europe** | |
| --- | --- |
| 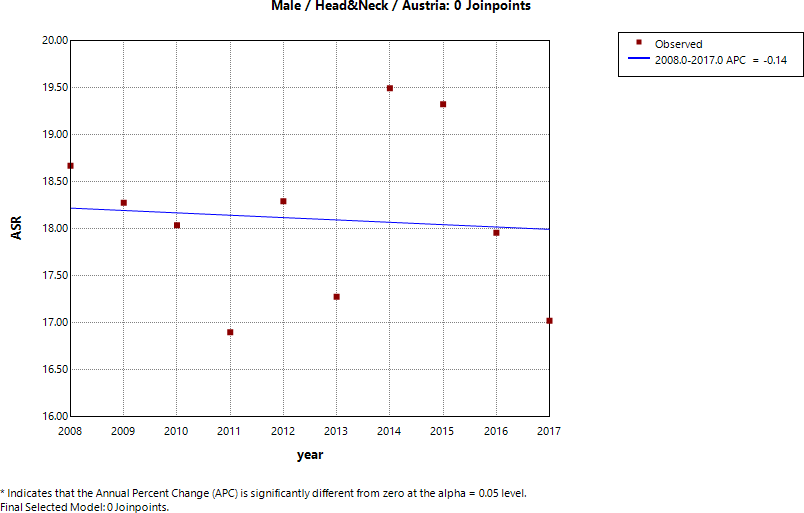 | 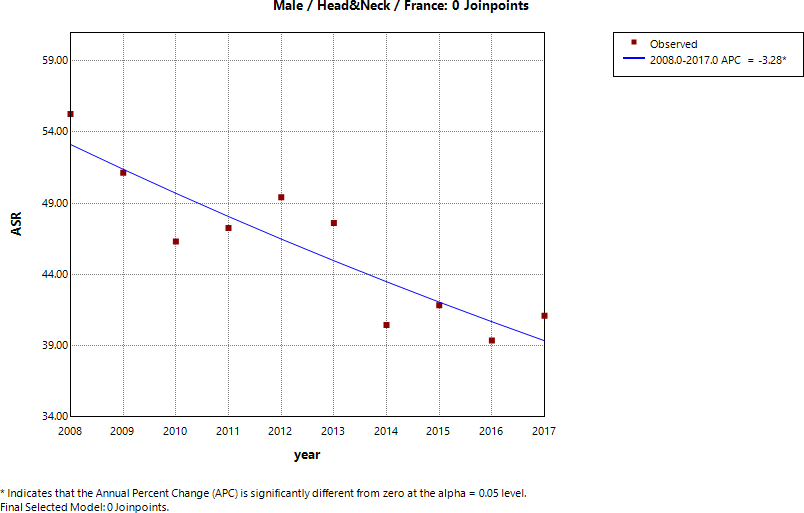 |
| 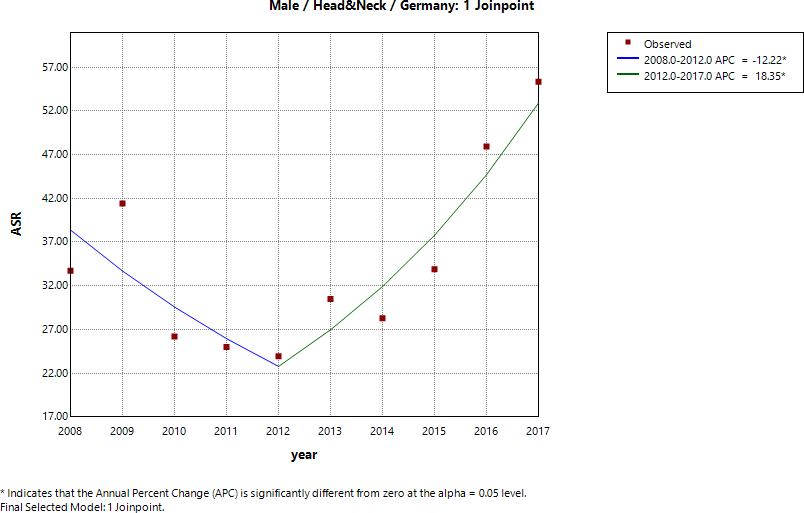 | 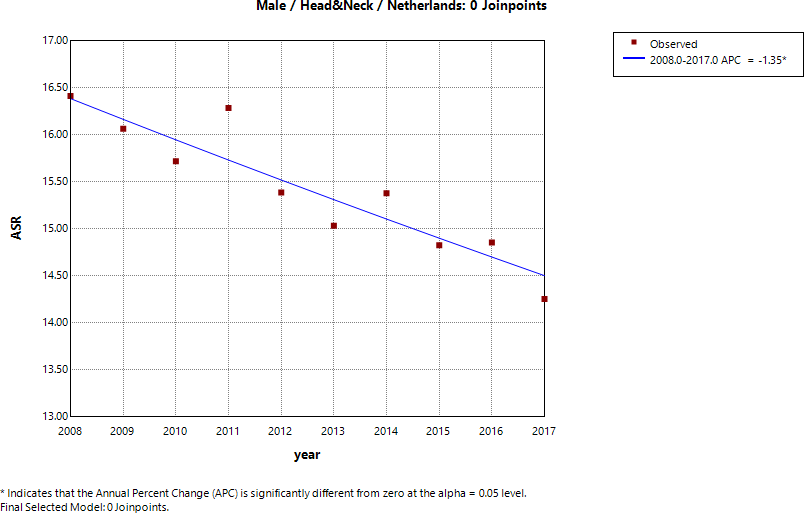 |
| 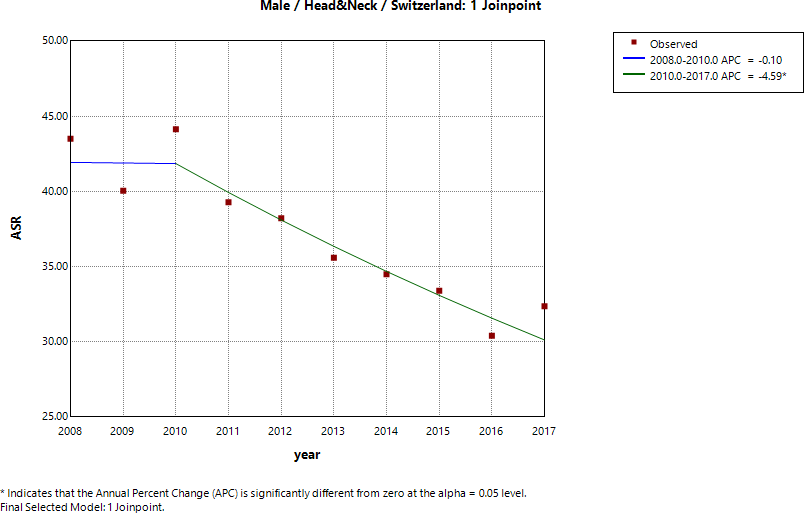 |  |
| **Southern Europe** | |

| **Southern Europe** | |
| --- | --- |
| 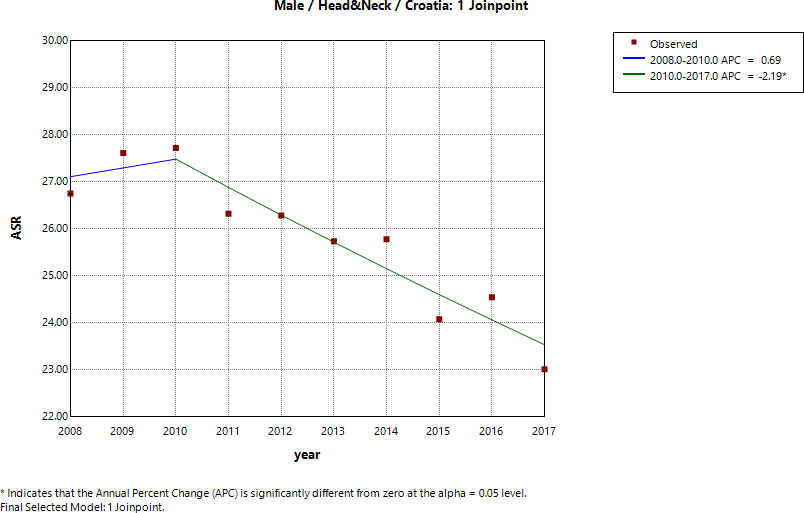 | 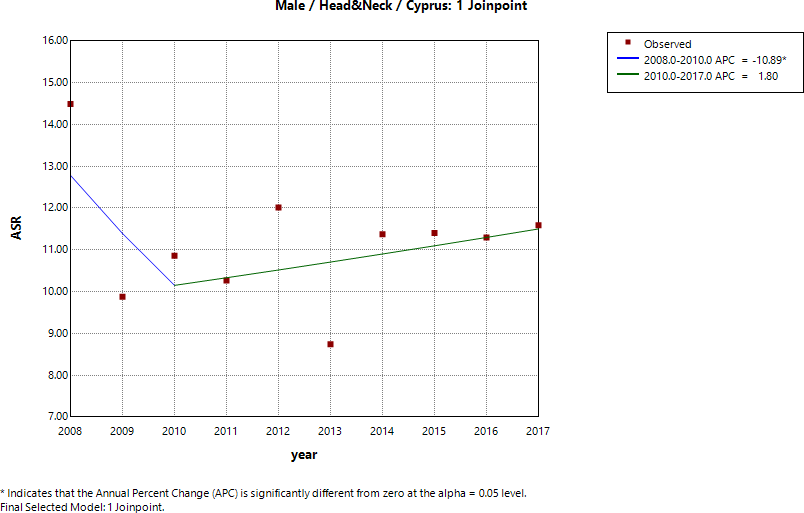 |
| 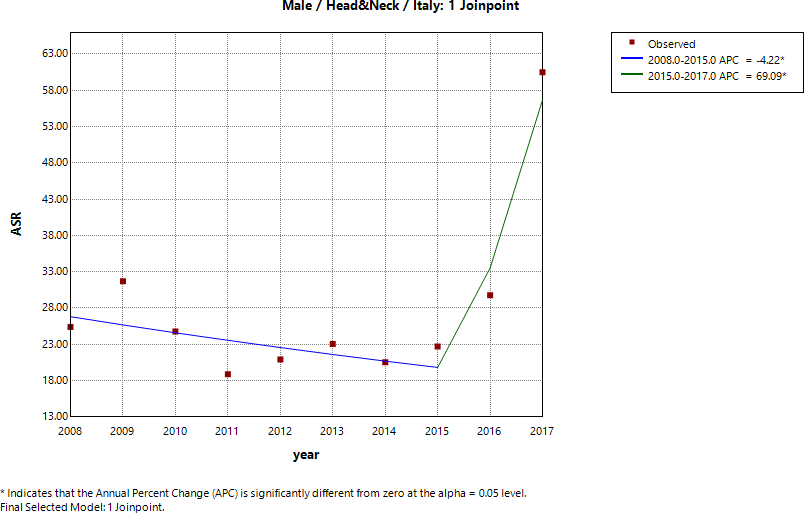 | 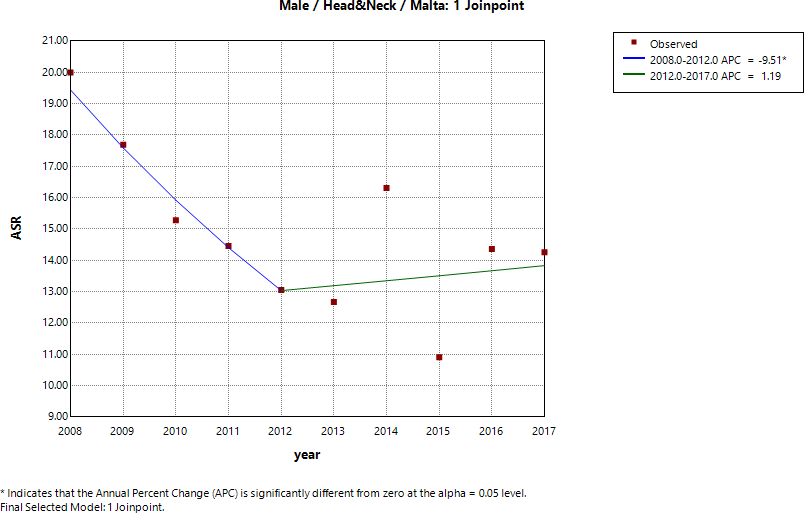 |
| 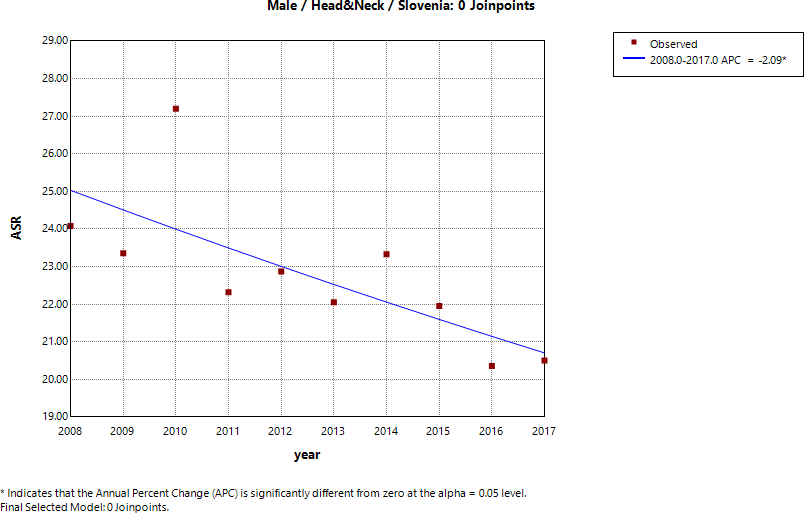 | 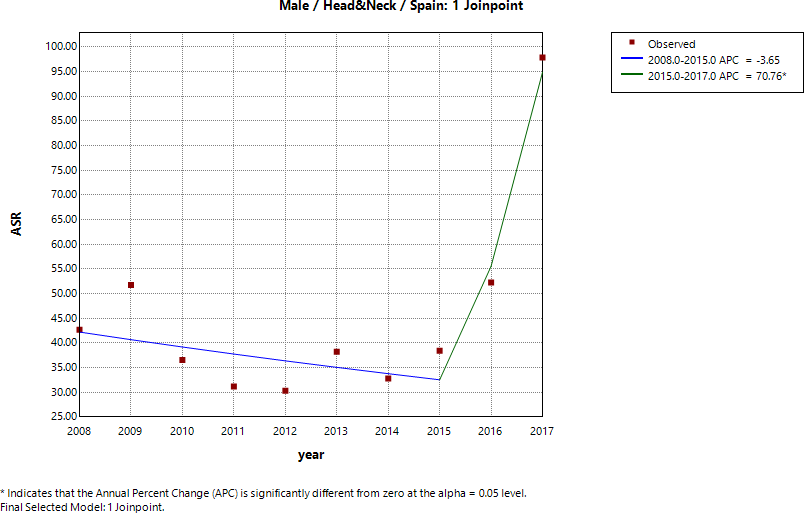 |

| **Eastern Europe** | |
| --- | --- |
| 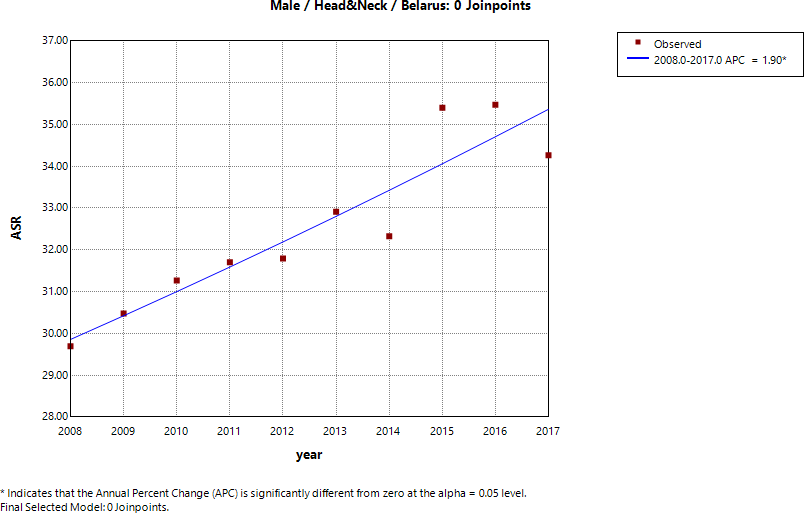 | 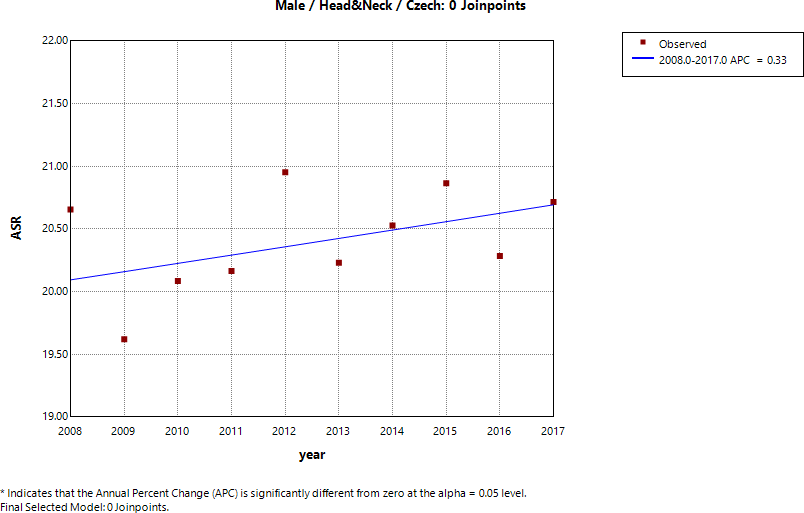 |
| 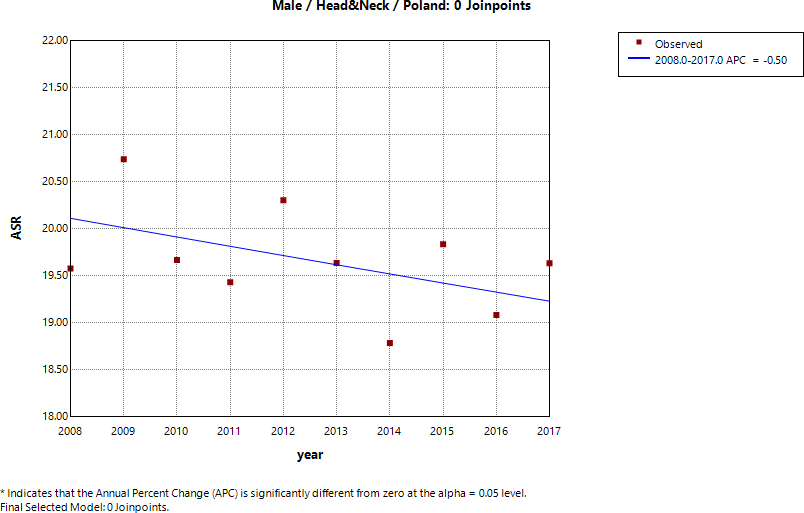 |  |
| **Africa** | |
| 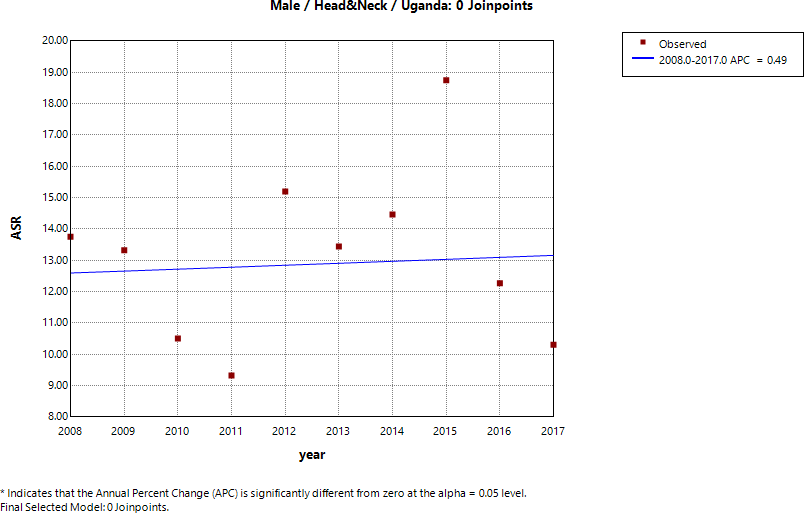 |  |

1c) Female

| **Asia** | |
| --- | --- |
| 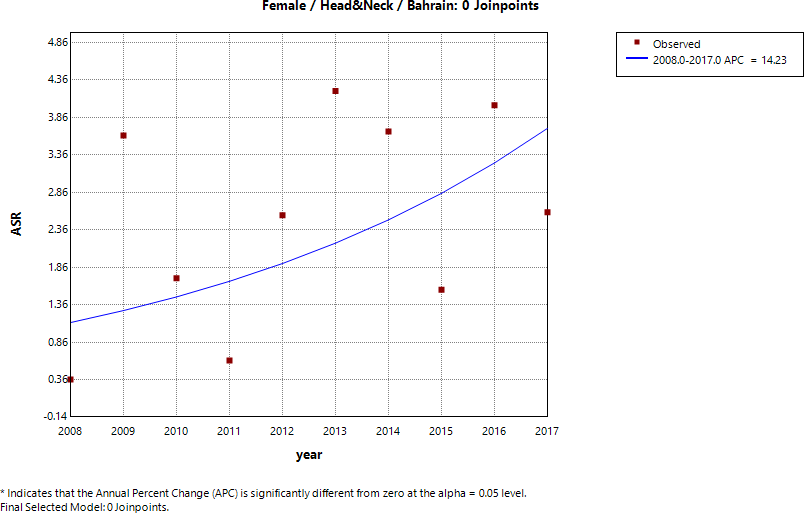 | 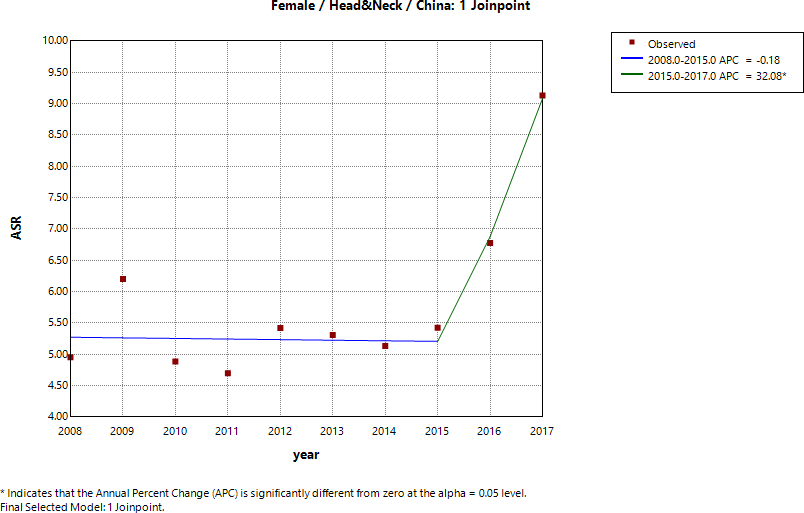 |
| 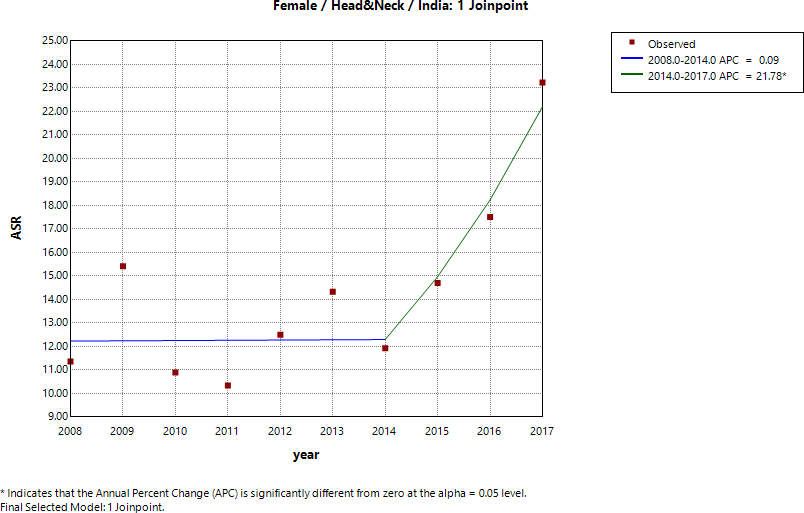 | 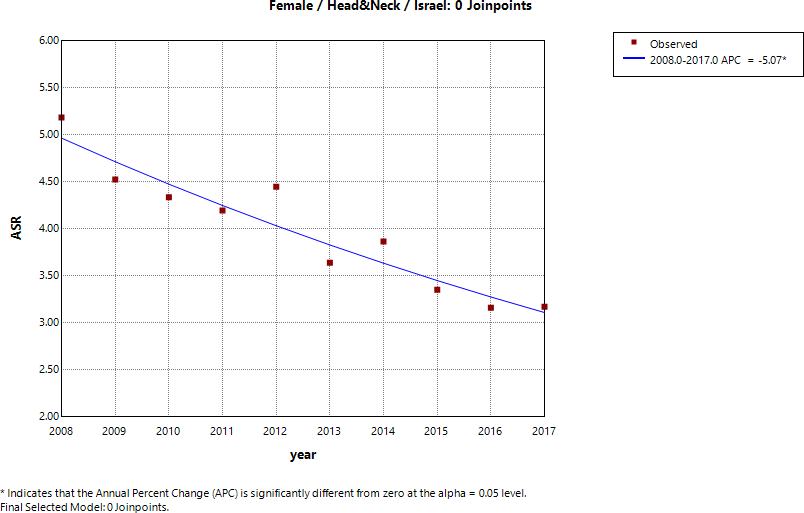 |
| 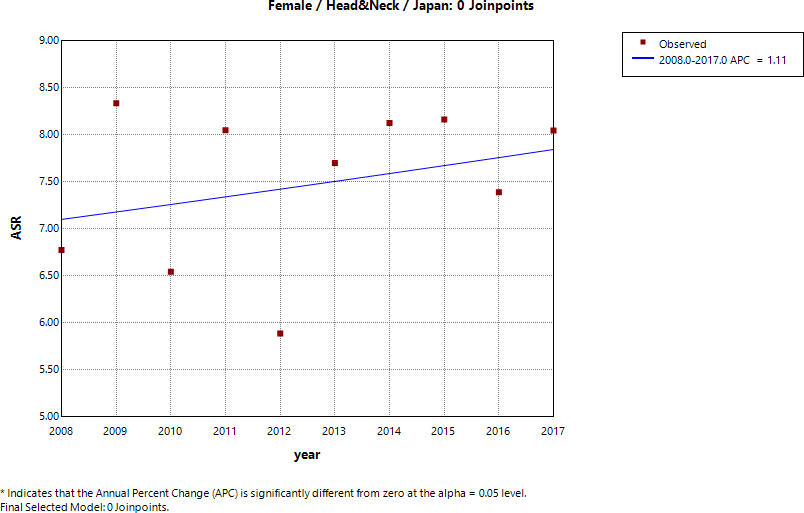 | 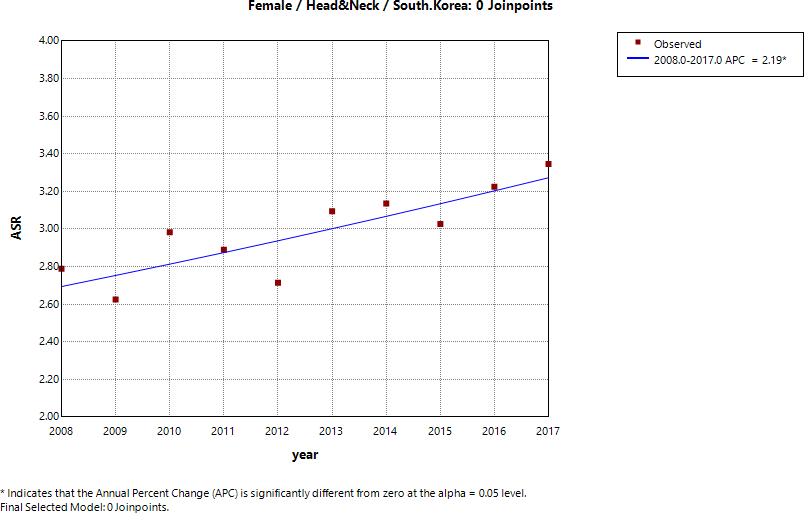 |

| 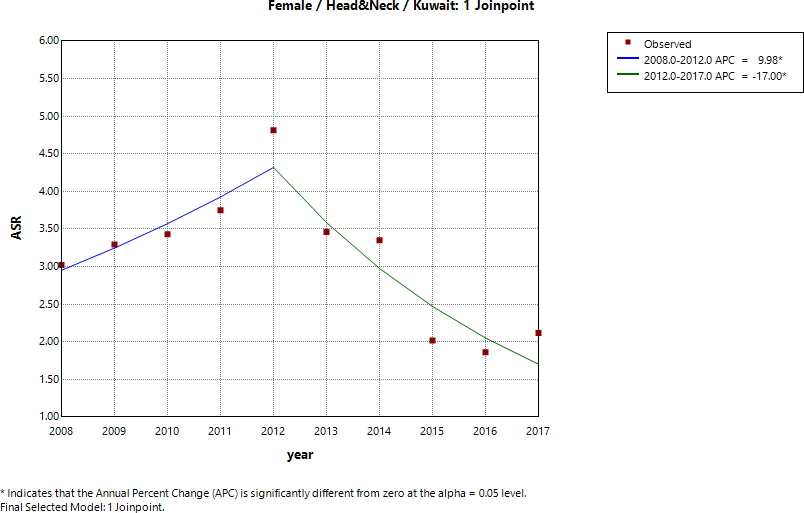 | 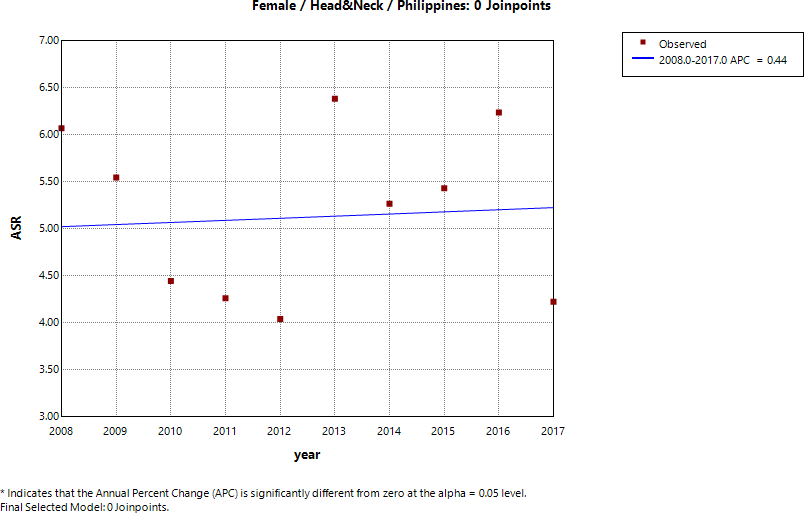 |
| --- | --- |
| 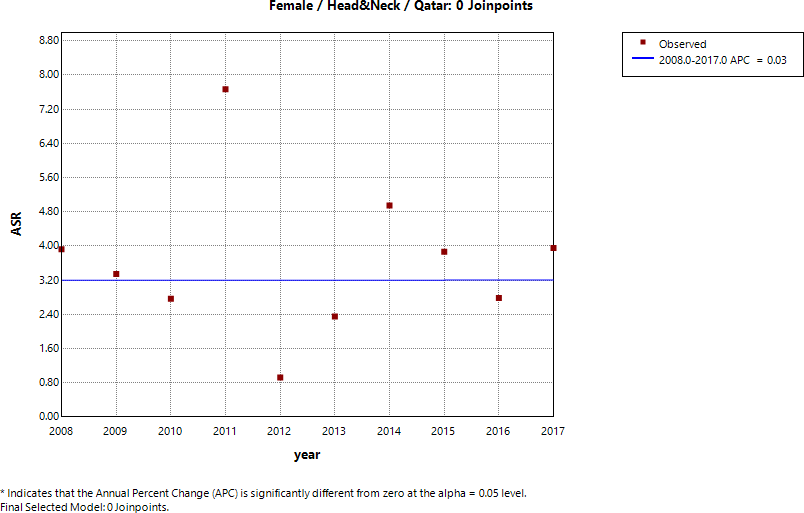 | 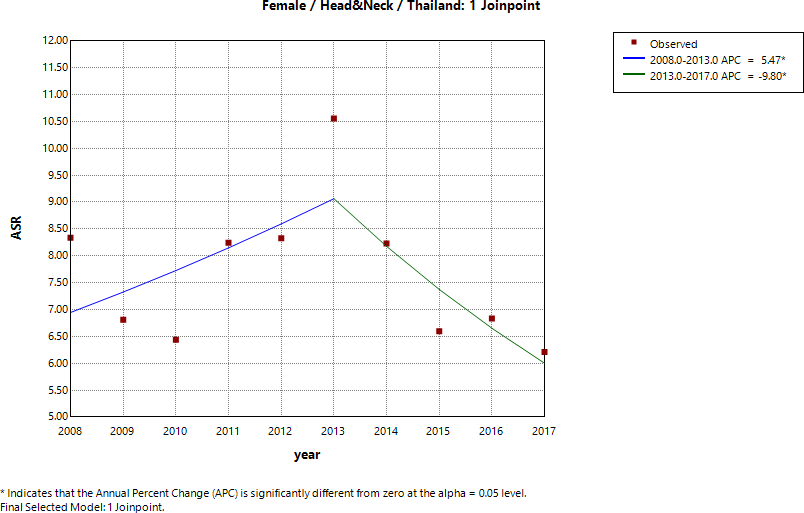 |
|  |  |
| **Oceania** | |
|  |  |

| **Northern America** | |
| --- | --- |
|  |  |
| **Southern America** | |
|  |  |
|  |  |

|  |  |
| --- | --- |
|  |  |
| **Northern Europe** | |
|  |  |
|  |  |

|  |  |
| --- | --- |
|  |  |
| **Western Europe** | |
|  |  |
|  |  |

|  |  |
| --- | --- |
| **Southern Europe** | |
|  |  |
|  |  |
|  |  |

| **Eastern Europe** | |
| --- | --- |
|  |  |
|  |  |
| **Africa** | |
|  |  |

1d) Young

| **Asia** | |
| --- | --- |
|  |  |
|  |  |
|  |  |
|  |  |

|  |  |
| --- | --- |
|  |  |
| **Oceania** | |
|  |  |
| **Northern America** | |
|  |  |

| **Southern America** | |
| --- | --- |
|  |  |
|  |  |
|  |  |
|  |  |

| **Northern Europe** | |
| --- | --- |
|  |  |
|  |  |
|  |  |
|  |  |

| **Western Europe** | |
| --- | --- |
|  |  |
|  |  |
|  |  |

| **Southern Europe** | |
| --- | --- |
|  |  |
|  |  |
|  |  |

| **Eastern Europe** | |
| --- | --- |
|  |  |
|  |  |
| **Africa** | |
|  |  |

1e) Old

| **Asia** | |
| --- | --- |
|  |  |
|  |  |
|  |  |
|  |  |

|  |  |
| --- | --- |
|  |  |
| **Oceania** | |
|  |  |
| **Northern America** | |
|  |  |

| **Southern America** | |
| --- | --- |
|  |  |
|  |  |
|  |  |
|  |  |

| **Northern Europe** | |
| --- | --- |
|  |  |
|  |  |
|  |  |
|  |  |

| **Western Europe** | |
| --- | --- |
|  |  |
|  |  |
|  |  |

| **Southern Europe** | |
| --- | --- |
|  |  |
|  |  |
|  |  |

| **Eastern Europe** | |
| --- | --- |
|  |  |
|  |  |
| **Africa** | |
|  |  |
